# The assessment of executive function abilities in healthy and neurodegenerative aging—A selective literature review

**DOI:** 10.3389/fnagi.2024.1334309

**Published:** 2024-03-26

**Authors:** Mojitola I. Idowu, Andre J. Szameitat, Andrew Parton

**Affiliations:** Centre for Cognitive and Clinical Neuroscience (CCN), College of Health, Medicine and Life Sciences, Division of Psychology, Department of Life Sciences, Brunel University London, Uxbridge, United Kingdom

**Keywords:** executive functions tasks, dual-tasking, inhibition, shifting, working memory updating, cognitive aging, mild cognitive impairment, Alzheimer's disease

## Abstract

Numerous studies have examined executive function (EF) abilities in cognitively healthy older adults and those living with mild cognitive impairment (MCI) and Alzheimer's disease (AD). Currently, there are no standard accepted protocols for testing specific EFs; thus, researchers have used their preferred tool, which leads to variability in assessments of decline in a particular ability across studies. Therefore, there is a need for guidance as to the most sensitive tests for assessing EF decline. A search of the most current literature published between 2000 and 2022 on EF studies assessing cognitively healthy older adults and individuals living with MCI and AD was conducted using PubMed/Medline, PsycINFO, Embase, Web of Science, and Google Scholar. Emphasis was placed on the EF's dual-tasking, inhibition, shifting or switching, and working memory updating. Many tasks and their outcomes were reviewed. Of particular importance was the difference in outcomes for tasks applied to the same group of participants. These various EF assessment tools demonstrate differences in effectively identifying decline in EF ability due to the aging process and neurodegenerative conditions, such as MCI and AD. This review identifies various factors to consider in using particular EF tasks in particular populations, including task demand and stimuli factors, and also when comparing differing results across studies.

## Introduction

A decline in executive functions (EFs) is a prominent feature of cognitive aging and neuropathological cognitive impairment, such as dementia (Deary et al., 2009; Mortamais et al., 2017; Cadar, 2018). Of particular interest are four core EF domains, dual-tasking, inhibition, shifting, and updating, which have been argued are fundamental for the accomplishment of many tasks in day-to-day life (Miyake et al., 2000b). These cognitive abilities are associated with an individual's level of independence and capacity to understand and coordinate their thoughts effectively. Neuroanatomical changes in the brain resulting in performance impairments in one or more of these EF domains have been reported in numerous studies of cognitively healthy (CH) older individuals and those living with mild cognitive impairment (MCI) and/or dementia, specifically Alzheimer's disease (AD) (Belleville et al., 1998; Wylie et al., 2007; Espinosa et al., 2009; Albinet et al., 2012; Johns et al., 2012; Clément et al., 2013; de Faria et al., 2015; Guarino et al., 2020; Rabi et al., 2020). These studies have employed a variety of tasks previously validated in diagnosing executive dysfunction because there are no generally accepted standard instruments for measuring executive dysfunction in any population.

### Assessing executive functions

Executive functions are heterogeneous and multifaceted (Norman and Shallice, 1986). Tasks employed to assess them normally depend on additional skills, such as language, visuospatial skills, or speed processing. To deal with such issues, EF tasks normally employ two or more conditions (e.g., congruent and incongruent, or repetition and shifting) that require matched supplementary skills but differ in the demand required by the specific EF. Therefore, the effects of supplementary skills can be removed by calculating the difference in performance between task conditions, i.e., the task cost. This task cost should quantify the effects on the specific EF (provided deficits in the supplementary skills do not entirely prevent task performance). Nevertheless, not all studies calculate cost measures, so a true representation of EF decline may not be observed, as any dysfunction reported may have occurred in one or more of the supplementary skills.

A further problem complicating the interpretation of results is that tasks created to assess a specific EF ability may require the contribution of other EF abilities (Lezak et al., 2012). For example, the random number generation task (Baddeley, 1998) requires both inhibition and updating ability for its successful completion. Please see Supplementary material for a full description of this and many of the tasks mentioned in the review listed under the EF they assess. Similarly, the behavioral assessment of the dysexecutive syndrome rule shift cards task (Wilson et al., 1996) requires the application of inhibition and shifting. Unlike supplementary skills, the task cost cannot identify the relative contribution of specific EFs to task performance deficits when the task involves multiple EFs (inhibition and shifting, in this example). However, additional measures of the EFs may potentially help disentangle the results.

These problems are complicated because performance in a particular EF task is not necessarily predictive of performance in another task measuring the same EF due to variations in sensitivity and specificity (Burgess et al., 1998; Huang et al., 2017; Fallahtafti et al., 2021). Currently, there is no clear consensus among researchers on how best to measure EFs, and a variety of tasks have been employed across various participant groups (Miyake et al., 2000a). This makes comparisons across studies difficult as (i) not all tasks are equally sensitive in assessing the decline of EF ability and may draw on other EF abilities, and (ii) even studies using the same task may employ different stimuli or modify task demands.

The primary aim of the current review is to examine factors affecting the assessment of EFs and their decline with age, particularly by comparing tasks in studies that employed multiple tasks on the same group of participants. This review covers recent studies published between 2000 and 2022 that assessed dual-tasking, inhibition, shifting, and updating by comparing cognitively healthy young and older adults and those living with MCI and/or AD. The review aims to determine the EF tasks most frequently employed for each of the four EF types and their practical utility within the different populations (normal and clinical) considered.

### Cognitive status

Determining the general cognitive status of participants is important, particularly for middle-aged and older individuals due to cognitive aging. However, there are several studies that do not screen participants in these age groups. Ebert and Anderson (2009) found that 25% of their supposedly CH older adult participants met the criteria for amnestic MCI (aMCI) following psychometric testing. Therefore, studies that did not confirm the cognitive status of their control group are likely to have included individuals with a form of pathological cognitive impairment.

This review only considered studies that assessed the cognitive status of their CH and pathologically impaired older adults with the mini-mental state examination (MMSE) (Folstein et al., 1975) or a modified form, i.e., 3MSE (modified MMSE) (Teng and Chui, 1987; Tombaugh et al., 1996). This is the most widely used screening tool for cognitive impairment. Still, it should be noted that a participant categorized as CH with the MMSE might not be considered the same if tested with more sensitive cognitive tests. When diagnosing dementia, the Alzheimer's disease assessment scale-cognitive subscale (ADAS-Cog) (Rosen et al., 1984), clinical dementia rating scale (CDR) (Hughes et al., 1982), Montreal cognitive assessment (MoCA) (Nasreddine et al., 2005) and Mattis dementia rating scale (DRS) (Mattis, 1976) are frequently used and have been considered to be more sensitive in rating the cognitive status of memory-impaired individuals than MMSE (Perneczky et al., 2006; Balsis et al., 2015; Pinto et al., 2019).

## Methods

A literature search of English language journal articles published between 2000 and 2022 was conducted in PubMed/Medline, PsycINFO, Embase, Web of Science, and Google Scholar databases. The search was based on a combination of key terms including Alzheimer's disease (AD), age-associated cognitive decline, cognitive decline, cognitive aging/ageing, cognitive impairment, dementia, dual-/multi-task or tasking, executive dysfunction, executive function(s), response inhibition, mild cognitive impairment (MCI), older adult, set-shifting, switching, working memory, working memory updating and updating (see Table 1 for examples with the initial number of publications found). The articles were then screened for suitability before being included in the review. Duplicate publications and review articles were removed, and publications in books, doctoral dissertations, master's theses, and newspapers were excluded from this literature review. Individual papers were then examined for relevance and additional references not revealed by the initial searches.

**Table 1:** Literature search combinations.

**Search term combination**	**Number of articles found**
Cognitive AND (ageing OR aging) AND (cognitive AND dual task) AND MMSE	17,800
Cognitive AND (ageing OR aging) AND response inhibition MMSE	19,000
Cognitive AND (ageing OR aging) AND set shifting AND MMSE	18,100
Cognitive AND (ageing OR aging) AND (working memory AND updating) AND MMSE	16,900
Mild cognitive impairment AND (cognitive AND dual task) AND MMSE	19,000
Mild cognitive impairment AND response inhibition AND MMSE	18,400
Mild cognitive impairment AND set shifting AND MMSE	18,600
Mild cognitive impairment AND (working memory AND updating) AND MMSE	17,600
Alzheimer's disease AND (cognitive AND dual task) AND MMSE	18,400
Alzheimer's disease AND response inhibition AND MMSE	18,400
Alzheimer's disease AND set shifting AND MMSE	18,000
Alzheimer's disease AND (working memory AND updating) AND MMSE	17,000

Eligibility included studies with CH older adult participants or those with a diagnosis of MCI or AD that employed a control group, i.e., comparing CH older adults with their younger counterparts. Studies were excluded if (i) they did not use the mini-mental state examination (MMSE) test (Folstein et al., 1975) or a modified form during the screening session to measure cognitive status; (ii) in the case of dual-tasking if one, or more, of the tasks was non-cognitive, i.e., assessing motor function involving walking or standing; and (iii) in the pathological cognitively impaired studies, MCI and AD was not a primary diagnosis of the participants, i.e., secondary to another condition.

## A review of executive function assessments from 2000 to 2022

### Dual-tasking

Dual-tasking, the simultaneous performance of two tasks (MacPherson, 2018), occurs in many day-to-day situations and is typically assessed by comparing single-task (ST) and dual-task (DT) performances (Della Sala et al., 1995). Between 2000 and 2022, the literature search identified 11 studies that compared cognitive DT ability between CH young and older adults, 9 comparing CH older adults and individuals living with MCI, and 12 comparing CH older adults and AD (see Supplementary Table S1). Two of these studies assessed both MCI and AD participants. Nine aging studies were excluded due to a failure to assess the cognitive status of their groups (Hartley, 2001; MacPherson et al., 2004; Bherer et al., 2005; Hartley and Maquestiaux, 2007; Logie et al., 2007; Hartley et al., 2011, 2015, 2016; Argiris et al., 2019), which leaves open to question the actual cognitive status of these “healthy” individuals.

The psychological refractory period (PRP) paradigm, or a variant, was the most frequently used (8 studies; 73%) to assess aging effects. The task requires participants to perform a single speeded response to a single stimulus (single task; ST) or two responses each to different stimuli (dual task; DT) presented at a range of different times relative to each other (i.e., 0, 100, 200 ms), which are termed stimuli onset asynchronies (SOA). A full description of all the tasks discussed in this review can be found in Supplementary Table S5. All included studies found significant declines in performance with age. In both ST and DT conditions, older adults were slower in completing tasks and made more errors than their younger counterparts. However, performance was especially impaired in DT conditions—they had higher DT costs [difference in the response times (RTs) and/or error rates between performance in the ST and simultaneous dual-task conditions], and these were generally higher for the shortest SOAs used by the researchers (termed the PRP effect). Strobach et al. (2012a,b) also reported that practice reduced DT costs in younger and older adults, but the older adults still showed a higher DT cost.

In general, the PRP paradigm is not performed in studies of pathological aging (as it is likely to be too difficult for these participants). For MCI participants, the most commonly employed task (three studies; 33%) was Baddeley's digit recall and tracking DT (Baddeley et al., 1986; Foley et al., 2013) while for AD groups the most frequently used paradigm (seven studies; 58%) was the Della Sala dual task (Della Sala et al., 1995), which is essentially a paper and pen version of the Baddeley's digit recall and tracking task. In Baddeley's digit recall and tracking DT, the recall condition requires participants to verbally repeat (in the same order) a series of digits immediately following the auditory presentation. Spans start at one digit and increase by a digit after the participant completes three trials at that length. In the tracking condition, participants follow the movement of a white square on a computer screen using a light pen. Both task conditions were performed separately (ST) and simultaneously (DT) (Baddeley et al., 1986).

Most studies of MCI groups reported maintained DT ability in digit recall and tracking. The exception was Lopez et al. (2006) with a mixed, i.e., consisting of a combination MCI group, indicating that the complexity and severity of MCI may affect the outcome measure. In contrast, the majority of the AD studies reported DT performance deficits. However, there is conflicting evidence as to whether it is preserved in mild/early AD (Lonie et al., 2009) or impaired (Perry et al., 2000), which may point to the need for a more sensitive assessment of patients. A fundamental issue is that there is unlikely to be a single DT paradigm that will distinguish deficits for all these groups, so care should be taken when selecting a task to choose one with established sensitivity for the groups tested or compared.

### Inhibition

Executive function inhibition is defined as the ability to control thoughts, attention, emotions, or actions to overcome a strong internal predisposition or previously prepared response (Diamond, 2013). Inhibition is commonly examined using interference paradigms where two stimulus features, each specifying a response, can either be the same (congruent) or different (incongruent) (Siéroff and Piquard, 2004; Scarpina and Tagini, 2017). Forty-eight studies were found to compare inhibitory control between young and older adults, 50 in MCI participants compared with healthy controls, and 49 AD participants (22 of these overlap with the MCI studies; see Supplementary Table S2).

The Stroop task (Stroop, 1935), or a variant, was the most frequently used (22 studies; 46%) in the aging studies. In the traditional version, participants complete three sections, each consisting of 100 items of a word naming (congruent), an ink color naming (congruent), and naming the ink color of the word (incongruent). Participants perform these tasks as quickly as possible, usually within a specific timeframe, e.g., within 45 s per section. All the studies that used this version and the modified versions of the Stroop task used by Maquestiaux et al. (2010) and Sylvain-Roy et al. (2015) reported age effects. Maquestiaux et al. (2010) version included a fourth task where participants shifted between identifying the color of the ink and reading the word aloud. In contrast, Sylvain-Roy et al. (2015) version presented all the three standard task conditions in random order in a single block. Both versions reported an age-related inhibitory decline. In addition to the standard version, Rey-Mermet et al. (2018) used a number of Stroop tasks where participants counted the number of centrally presented numeric characters whilst ignoring their numeric values (which differed from the number of characters in incongruent conditions). Surprisingly, the older adults actually showed lower interference effects than younger adults, which contrasted with their increased interference effects in the standard Stroop version and led the authors to question the general applicability of the concept of inhibition.

Evidence from variants of the Stroop task appears to suggest that the underlying nature of the stimuli and task may affect whether aging effects are observed. For example, age-related performance deficits have been shown in variants of the emotional Stroop task (Agustí et al., 2017; Kamboureli and Economou, 2021). In Agustí et al. (2017), participants viewed emotional faces (happy or sad) with the words happy or sad superimposed over them and in separate blocks, participants responded to the words or faces in both congruent and incongruent conditions. Kamboureli and Economou (2021) used a standard emotional Stroop task where participants named the color of words that could be associated with a negative or neutral emotion.

There is some evidence that the non-verbal Stroop task is less susceptible, or sensitive, to age-related decline. Pettigrew and Martin (2014) employed a nonverbal Stroop comprising three conditions—a neutral condition, where participants were presented with a stimulus in the center of a computer screen, e.g., a left-pointing arrow; a congruent condition, where the stimulus was on the same side the arrow pointed, e.g., a left-pointing arrow on the left side of the screen; and an incongruent condition, where the stimulus was on the opposite side to where the arrow was pointing, e.g., a left-pointing arrow on the right side of the screen. The participants were required to respond with the direction the arrow was pointing, right or left. They also performed the picture-word interference task (Lupker, 1979; Schriefers et al., 1990) involving the completion of two conditions—an interference condition, where a picture is superimposed with a distractor word from the same semantic category, and a non-interference condition, where a picture is superimposed with a distractor word from a different semantic category. Participants were required to identify the picture while ignoring the word. No effect of aging was reported with this task, although significant age-associated performance deficits were observed with the same group of participants with the traditional and non-verbal Stroop tasks.

For pathological aging, the standard Stroop, or a variant, was the most frequently employed task for both MCI (32 studies; 64%) and AD (37 studies; 76%) participants. Seventy-two percent of the MCI studies observed a decline in inhibitory capacity, while all but one of the AD studies (Bélanger and Belleville, 2009) reported deficits. As with the aging studies, the emotional Stroop was used in two studies, Meléndez et al. (2020) and Satorres et al. (2020), on AD participants, and both found inhibitory decline. Further, the picture Stroop was used by Nordlund et al. (2005) and Duong et al. (2006), the math Stroop used by Zamarian et al. (2007), and the numerical Stroop by Kaufmann et al. (2008) (see Supplementary material for full descriptions of these studies). All reported inhibitory decline and the absence of this decline in these tasks for healthy aging may indicate that they may help selectively identify pathologies.

The most common Stroop variant in these populations was the Victoria version (Spreen and Strauss, 1998) where participants were presented with three stimulus cards comprising 24 items and asked to either name the color of dots (dot condition), name the color of the ink of the neutral words (word condition), or name the color of the ink of color word names (interference condition, as the color was always different from the word). This is a briefer and generally easier task to perform than the traditional Stroop, and no group of MCI participants showed performance deficits relative to controls, which suggests that it is of limited utility in these patient groups and should be considered when designing studies (or reviewing manuscripts). However, with the traditional Stroop, performance deficits were reported in most of the studies that assessed MCI participants, which further emphasizes the need to use appropriately sensitive tools to assess deficits in different groups.

This is further supported by the results of the Duong et al. (2006) study. They reported inhibitory decline with the picture Stroop, a task involving the presentation of an animal drawing (either rabbit, horse, bear, or cow) in four conditions defined by text superimposed over the picture. These conditions were (i) neutral with “xxxx” text, (ii) congruent with the name of the pictured animal, (iii) incongruent/same with the name of one of the other animals, and (iv) incongruent/different using the name of an animal that was not pictured in other trials. Surprisingly, participants showed no deficits with the Victoria Stroop.

### Set-shifting

Set-shifting (or task switching) is the ability to effectively move back and forth between two tasks (Miyake et al., 2000b). It is mainly examined by comparing task performance under the following two conditions: (i) a repetition condition where participants perform the same task repeatedly in a block, and (ii) a shifting condition where participants switch (or shift) between two tasks pseudo-randomly within the same block. Twenty-nine studies were identified comparing performance between CH young and older adults, 45 studies assessing older individuals living with MCI participants, and 43 researching AD participants (18 of which overlap in also assessing MCI participants) (see Supplementary Table S3).

The trail-making task (TMT) (Reitan and Wolfson, 1986; Reitan, 1992) was the most commonly used in aging studies (17 studies; 59%). This test comprises two parts—part A requires participants to connect 25 numbered (1, 2, 3, etc.) dots or circles in sequential order, and part B requires participants to alternate connecting letters and numbers in ascending order (1, A, 2, B, etc.). Seventy-two percent of studies reported an age-associated decline (see Supplementary material), but there was some inconsistency in the outcome measures reported. For example, some studies reported the TMT cost (the difference between RT or error rate in parts A and B; see, for example, Tournier et al., 2014 and Yordanova et al., 2021); others reported the measures of TMT parts A and B separately or just the part B measure (the shifting measure of the task without considering the non-shifting condition; see Müller et al., 2014); and Rey-Mermet et al. (2018) reported the TMT ratio. Therefore, shifting ability and deficits may not be confidently compared across studies, and it is recommended that researchers should report scores for parts A and B alongside ratio or cost scores.

The trail-making task was also the most frequently used task in studies assessing both MCI (34 studies; 76%) and AD participants (29 studies; 67%). For MCI participants, there was generally a decline in shifting ability, whereas for other EF abilities, there were variations across different subtypes of MCI (Loewenstein et al., 2006; Silveri et al., 2007). Loewenstein et al. (2006) reported a deficit in shifting ability in MCI participants diagnosed with prodromal Alzheimer's disease but not in those with MCI diagnosed with vascular disease. Similarly, Silveri et al. (2007) reported impairments in their mixed MCI-type participants but not their aMCI or non-aMCI participants. In contrast, Zheng et al. (2014) reported performance deficits in aMCI based on TMT part B, highlighting the problem of using different outcome measures. In AD participants, all studies, except Price et al. (2009), reported a significant deficit in shifting ability, possibly due to the presence of AD pathology in the non-demented older adults, resulting in a decline in their cognitive function. However, they were not clinically diagnosed with AD.

In addition to the standard TMT, other variants were also employed by researchers who reported shifting deficits in both MCI and AD participants, indicating the generality of the findings from the standard TMT. These include an oral TMT (Bastug et al., 2013), the letter-number TMT (Pa et al., 2010), modified TMT (Kramer et al., 2006; Chen et al., 2013; Heuer et al., 2013), an alternating trail-making (Zheng et al., 2014), and a color trail test (D'Elia et al., 1996). The oral TMT (Bastug et al., 2013) required participants to either verbally count from 1 to 25 (part A) or to alternate between counting numbers and listing the alphabet, e.g., 1-A-2-B (part B).observing shifting performance deficits in aMCI and AD participants, analogous to those found with the traditional TMT. The letter-number TMT (Pa et al., 2010) is very similar but has two non-shifting parts: a letter only and a number only. In the version used by Chen et al. (2013) and Heuer et al. (2013), participants had to serially alternate between numbers and days of the week. In all these studies, shifting deficits were reported in MCI and AD participants. Further, studies have reported deficits only in AD patients, but in the case of Heuer et al. (2013), the absence of effects in other groups (MCI and healthy controls) appears due to ceiling effects in an easy TMT task. Two studies in AD patients used a color TMT paradigm, which required participants to connect circles numbered 1 to 25 in ascending order (part A) or connecting sequentially numbered circles whilst alternating between two circle colors, i.e., 1-pink-2-blue-3-pink-, etc. (part B) (McGuinness et al., 2010; Huang et al., 2017). Both studies reported significant performance deficits in their AD participants, though Huang et al. (2017) used only part B of the test.

### Working memory updating

Updating, defined as the continuous changing of content in working memory (WM), is examined by tasks that require the manipulation of the content of WM (Miyake et al., 2000b). Thirty-six studies compared young and older adults, whilst 35 examined CH older adults and participants with MCI, and 32 compared CH older adults and participants with AD. Seventeen of these studies assessed both MCI and AD participants (see Supplementary Table S4).

From these, the n-back task (Kirchner, 1958; Jaeggi et al., 2010) was the most commonly employed task in aging studies (12 studies; 33%), which required participants to keep a continuous memory of stimuli, and they typically had to respond to whether the current stimuli matched one that occurred n items earlier. Across these studies, the span lengths ranged from 1- to 4-back (with 2-back being the most common). All the studies reported an age-associated decline in updating ability at various n-back lengths and stimuli. Amer and Hasher (2014) utilized a 1-back task with a word or non-word superimposed upon a picture as stimuli. Participants responded every time when either two consecutive pictures were identical, or two consecutive pictures were different whilst disregarding the superimposed words or nonwords. Clarys et al. (2009) and Boucard et al. (2012) required participants to listen to a sequence of letters and determine if the current letter matched the letter heard two letters prior. The generality of the findings across sensory domains was evident when participants viewed a sequence of letters and matched stimuli in a 2-back task (Daffner et al., 2011) or 3-back task (Missonnier et al., 2011; Nagel et al., 2011) or when tested with visual stimuli (Berger et al., 2017; Peng et al., 2020). In the 1-back task Peng et al. (2020) used, participants had to identify if the object presented on the screen was the same or different from the object presented immediately before. Berger et al. (2017) employed 1- and 2-back using faces of emotional expressions (“angry,” “neutral,” or “happy”) and age groups (“young,” “middle-aged,” or “old”) where participants had to identify either whether the expression or age group was the same or different. The general conclusion across all these studies was that older individuals were less proficient at suppressing irrelevant information, and this problem increased with load demand during the 2- and 3-back conditions. Whilst younger individuals were also affected by the increased load in n-back tasks, their performance remained more accurate and faster than older participants. The increased susceptibility of older participants to irrelevant information was tested directly by Kato et al. (2016), who used two versions of the n-back—a non-distractor and a distractor. In the non-distractor, participants must identify if the word presented is the same as the previous one (1-back task) or two items prior (2-back task). In the distractor condition, a sound was played, which the participants had to ignore. They found that the performance of the older participants was disrupted by the sound, whilst that of the younger participants was unaffected.

Due to its ease of administration in cognitively impaired groups, the backward digit span (BDS) (Griffin and Heffernan, 1983; Wechsler, 2012; Egeland, 2015) was the most commonly utilized task in studies of both MCI (28 studies; 88%) and AD (25 studies; 81%) populations, which included 15 studies testing both groups. The BDS requires participants to immediately repeat a list of digits they had just heard in reverse order. The span length ranges from two to eight digits, with each span length typically completed two times.

Findings in studies of MCI patients have been nearly evenly split between those reporting deficits in the backward digit span and those indicating it was unaffected with this variation relating in part to different sub-types of MCI (Lopez et al., 2006; Zhou and Jia, 2009; Doi et al., 2013). Zhou and Jia (2009) examined participants in the prodromal phase of AD (MCI-AD) and with MCI caused by cerebral small vessel disease (MCI-SVD) but only found WM updating deficits in the MCI-SVD participants. Doi et al. (2013) found substantial performance impairment in late-stage aMCI participants but not early-stage aMCI, and differences along this spectrum of severity may explain variations in the findings for aMCI across studies. This is partially supported by Lopez et al. (2006), who assessed aMCI and a broad cognitive impaired MCI type (MCI-multiple cognitive domain type; MCI-MCDT) and found aMCI preserved WM updating ability while in the MCI-MCDT, it was impaired. Meanwhile, Emrani et al. (2018) examined aMCI and combined mixed domain/dysexecutive MCI type participants and reported deficits in both groups. However, more performance errors were produced in the combined mixed domain/dysexecutive MCI individuals, particularly at position 5 of a span length of 5, implying worsened impairment in comparison to the aMCI participants. Therefore, these findings may indicate that the BDS task may be able to distinguish between severity and subtypes of MCI. However, further studies are needed to assess MCI subtypes employing different updating tasks to tease out the nature of underlying deficits.

In terms of AD, most of the studies reported a decline in WM updating ability but not necessarily in other dementia types for both mild and moderate participants (Ferreira et al., 2019). Smits et al. (2015) examined patient groups with multiple dementia types and found impaired WM updating for vascular dementia, dementia with Lewy bodies, and AD participants but not frontotemporal dementia and language variant frontotemporal dementia participants. Similarly, Pa et al. (2010) reported deficits for AD and cortical basal degeneration but not their amyotrophic lateral sclerosis, MCI, frontotemporal dementia, or semantic dementia groups. Crawford et al. (2013) reported impairments for AD but not their Parkinson's disease participants. Therefore, the task may help distinguish between dementia types, but more work is needed on teasing out the underlying basis of deficits as this may also vary across groups.

### Section conclusion

To conclude, numerous tasks have been employed to examine EFs in CH adults and individuals living with MCI and AD. The most frequently employed tasks in cognitive aging studies for dual-tasking ability were the PRP paradigm for inhibition ability, the Stroop task for shifting ability, the TMT, and updating the n-back task, similar to findings reported by de Faria et al. (2015). In the cognitively impaired participant studies, dual-tasking was frequently evaluated with the Della Sala DT, inhibition ability with the Stroop task, shifting ability with the TMT, and updating with the BDS task. The decline in each of these EFs was largely reported with all the tasks, particularly in the AD groups.

The assessment of dual-tasking and WM updating was largely dependent on the cognitive status of the participants, i.e., CH older adults and the MCI/AD groups. The PRP and n-back tasks reviewed to be most commonly employed with the CH older adults are both computerized and require more technical ability for their completion in comparison to the pen-and-paper common tests, Della Sala DT, and BDS task, found with the MCI and AD groups. Consequently, the ease of application of these latter tests was a major factor.

Interestingly, the BDS task seemed to be good at distinguishing between the subtypes and severity of MCI (Lopez et al., 2006; Zhou and Jia, 2009; Doi et al., 2013), although Chang et al. (2010) reported an insignificant difference in performance between lower and higher EF participants. Also, AD was reported to greatly affect WM updating irrespective of the task used and, on some occasions, to a greater degree than other dementia types.

The traditional Stroop task was generally sensitive enough to detect inhibitory deficits in cognitive aging and degenerative neuropathological conditions. Variations between the modified and alternative Stroop types, typically the Victoria Stroop, demonstrated the difference the makeup of a task can make. The Victoria Stroop is simpler than the traditional Stroop, and it appeared to show little sensitivity to MCI; hence, it may have a role in separating MCI from AD. However, it should only be used with a strong theoretical rationale. A final significant point is that the baseline conditions are not the same in these tasks, and thus, the inhibitory cost measure should be considered.

The TMT was the most employed task for shifting ability in aging and neurodegenerative studies, with a large proportion reporting a decline in shifting ability. However, the differences in outcome measures, i.e., part B measure, cost measure, or ratio, used by researchers may account for this and thus demonstrate the importance of standardizing tasks and reporting the outcome measures.

## Discussion of EF task variables

Differences in the demand characteristics, sensitivity, outcome measure, and stimuli were observed with all the EF tasks discussed in the prior sections. This section will briefly discuss these factors.

### Stimuli

The type of stimuli used in a task may affect their sensitivity in detecting impairments in EF tasks. An important observation from the reviewed studies is that emotional stimuli may be more robust to the inhibitory effects of aging (Agustí et al., 2017; Dupart et al., 2018; Waring et al., 2019; Williams et al., 2020; Kamboureli and Economou, 2021), as well as between the CH older adults and individuals living with AD (Meléndez et al., 2020; Satorres et al., 2020).

For instance, Agustí et al. (2017) used an emotional Stroop task, which consisted of the word happy or sad, superimposed on happy or sad facial expressions in congruent and incongruent trials. Participants completed face or word-only trials where they had to identify the emotion of the face or the written word only, respectively. Greater interference was observed for the trials of positive faces and words in the young and older adults assessed, with the older adults showing an age effect, particularly with the positive faces, suggesting an inclination toward positive stimuli. This was similarly seen with the AD participants in the Meléndez et al. (2020) study. Interference was greater for positive stimuli and facial trials, increasing as the severity of AD increased from mild to moderate AD. However, the interference for negative and positive facial stimuli was comparable. Satorres et al. (2020) did not report the baseline measures of their trials.

Dupart et al. (2018) used the emotional Hayling Sentence Completion Test (HSCT). The traditional HSCT (Burgess and Shallice, 1997) requires participants to complete a cloze sentence with a missing last word. In part A, the initiation section with the congruent condition of the test, a related, expected word should be provided. In part B, the inhibition section with the incongruent condition, an unrelated, unexpected word should be provided. The emotional type utilized emotionally charged sentences and compared the words the participants produced as either emotionally neutral, positive, or negative. The older adults were slower at generating negative words than neutral words, in contrast to young adults in the initiation section of the test. However, both groups were faster at producing positive words than neutral words in both the initiation and inhibition sections, indicating that positive stimuli may influence better performance in such a task, i.e., positive emotion had a sufficient effect on both populations. Nonetheless, RT age effects were reported for both sections in all the conditions, i.e., between the neutral and negative conditions and between the positive and neutral conditions. This result is supported by Williams et al. (2020), who reported that happy facial expressions aided response inhibition in both young and older adults in contrast to fearful faces with the employment of the stop-signal task. Only older adults showed the benefit of happy facial expressions compared to neutral expressions.

Waring et al. (2019) reported better response inhibition (i.e., fewer false alarms) in older adults for an emotional facial expressions go/no-go task compared to younger adults, indicating better emotional regulation among older adults. The task involved the participant being cued to which facial expression was the “go” stimuli and the other the “no-go” stimuli, with each facial expression serving as a “go” stimulus. The task combinations were fear/neutral, neutral/fear, happy/fear, fear/happy, happy/neutral, and neutral/happy. All these findings are consistent with research on the emotional effect of performance across several cognitive domains (De Houwer and Hermans, 2010).

Additionally, several studies used the same task but with different types of stimuli (Nordlund et al., 2005; Duong et al., 2006; Zamarian et al., 2007; Salthouse, 2010; Vallesi et al., 2010; Bastug et al., 2013; Pettigrew and Martin, 2014; Agustí et al., 2017; Rey-Mermet et al., 2018; Meléndez et al., 2020; Satorres et al., 2020; Kamboureli and Economou, 2021; Ward et al., 2021, see Supplementary material for more detail). Two of them (Nordlund et al., 2005; Duong et al., 2006) observed different findings with the same group of MCI participants using the picture Stroop and Victoria Stroop versions. Both reported a decline in inhibitory ability with the picture Stroop task but not with the Victoria version.

Using two sets of stimuli for the task-switching paradigm, an addition–subtraction and a left–right, on CH older adults and in those suffering from MCI and AD, Belleville et al. (2008) reported high shifting costs with the left–right spatial task only. MCI participants presented with larger global switch costs (performance in the switch block minus the repetition block). In contrast, AD showed both increased global and local costs (performance in the switch trials minus the repetition trials within a switching block).

Two variant tasks of the BDS were also employed in addition to the traditional BDS task by two researchers—Sung et al. (2012), with the word backward span and Kessels et al. (2015), with the backward spatial span. The word backward span is essentially the same as the backward digit span but with words instead of digits. Participants are read various increasing span lengths of words and are required to immediately verbally recall the span of words in reverse order. The backward spatial span requires participants to recall various sequence spans presented on a screen in reverse order. Sung et al. (2012) reported updating deficits in their MCI participants with the BDS as well as the alphabet span but not the backward word. Kessels et al. (2015), who updated ability in MCI and AD, only reported performance deficits in their AD participants with the backward spatial span task. Whereas with the BDS task, deficits in both the MCI and AD participants were observed. This result was also reported during the assessment with the forward spatial span (Wechsler, 1987) and forward digit span (Wechsler, 2012). Kessels et al. (2015) concluded that the spatial test of WM load was limited and less vulnerable to subtle impairments. Therefore, it may be concluded that the area of the brain associated with visuospatial WM processes may not be as affected as that associated with verbal WM processing (Donolato et al., 2017) or lexical processing in MCI participants.

Therefore, the modality, type, and/or nature of the stimuli used in a task may account for performance differences in research and, as such, should be considered during task selection. As highlighted in this section, the use of letter, lexicon, numerical, and spatial stimuli as opposed to photos or images, such as faces, particularly emotional in nature. It would be interesting to know if using different stimuli produces the same type of performance shortfall. However, the overall task may further differ in other parameters as well. Therefore, we recommend standardizing the stimuli.

### Demand

Variations in the cognitive demands within and across a task may relate to a decrease or increase in the number of different cognitive processes and/or functions required for performing and successfully completing a task; more specifically, differences in the task demand, which researchers may choose to modify to reduce or increase performance difficulty in their participants. As such, this may eliminate and/or recruit additional cognitive processes or simply place greater strain on those already used. This review found a number of modified tasks (Kramer et al., 2003, 2006; Amieva et al., 2004; Maquestiaux et al., 2010; Endrass et al., 2012; Hsieh and Fang, 2012; Hsieh et al., 2012, 2016; Chen et al., 2013; Heuer et al., 2013; Van Dam et al., 2013; Wang et al., 2013; Sylvain-Roy et al., 2015), as well as various forms of the set-shifting task, the WSCT, in both the age effect studies and the neurodegenerative studies (Perry et al., 2000; Calderon et al., 2001; Hartman et al., 2001; Traykov et al., 2002, 2007; Nagahama et al., 2003; Nordlund et al., 2005; Stokholm et al., 2006; Guild et al., 2014; Oosterman et al., 2014). Amieva et al. (2004) modified the Stroop task by having two Stroop types—an interference (naming the color of the word in the incongruent condition) and a reverse Stroop (reading the word in the incongruent condition).

Nonetheless, the employment of these modified tasks is not always clearly defined. We would suggest that researchers conduct and report the unmodified original task results in addition to the modified parameters of the new task results for a better understanding of how participants are affected, as seen in Hartman et al. (2001), with the use of the standard Wisconsin card shifting task (WSCT) and a modified version which included visual cues to remind the participant of the most recent sort. In the standard WSCT (Berg, 1948; Nelson, 1976) task, participants are presented with a number of stimulus cards with sets of symbols that vary in color, shape, and number (e.g., three green triangles or two yellow squares). They are instructed to categorize them according to a particular dimension (i.e., color, shape, or number). The category rule changes every time 10 (out of a maximum of 128) response cards have been sorted correctly, but the participants are unaware of this pattern. The modified flanker task, first introduced by Van't Ent (2002) and used by Hsieh and Fang (2012) and Hsieh et al. (2012), combines the standard task (please see Supplementary material for description) with compatible (PRO) and incompatible response (ANTI) conditions. In the PRO condition, responses correspond to the target arrow, i.e., a right arrow is responded to with the keyboard button “M” and left with “Z,” but in the ANTI, the opposite applies. To distinguish between the conditions, different colors were used for the target arrow. A bias condition was also incorporated where the target arrow was flanked by rectangles. All conditions were performed as congruent and incongruent tasks. Similarly, Hsieh et al. (2016) employed three demand levels of an adapted go/no-go task (Newman and Kosson, 1986) by using various percentages of “go” and “no-go” conditions, i.e., a low demand version with 20% of the task “go” and 80% “no-go,” an equivalent demand of 50% “go” and 50% “no-go,” and a high demand of 80% “go” and 20% “no-go” in an inhibitory study between CH young and older adults. The fewer “no-go” conditions resulted in less response time. In addition, two types of “no-go” stimuli were used—an irrelevant type, where the stimulus was from the same category as the “go” stimulus, i.e., a letter with a letter, and a conflict type, where the stimulus was from a different category, i.e., a letter with a number. Accordingly, a fair comparison of the performance outcomes could be made within and across studies using a heterogeneity of modified tasks and their demands. Thus, the demand for EF tasks may be revised for a desired outcome or to reduce the possibility of floor (too difficult) or ceiling (too easy) effects in the group being assessed.

### Sensitivity

The sensitivity of tasks refers to how well its findings detect an effect based on the effect size^1^ or statistical power^2^ of the research conducted, such as the power of EF tasks in assessing their intended cognitive process. A few studies in this review were observed to have employed multiple tasks to assess the same EF on the same group of participants, with a proportion reporting converging results between two or more tasks, suggesting these tasks measured the same cognitive process with the same power. Of particular interest, however, were the studies with tasks that reported conflicting findings with two or more tasks. This was observed in the aging studies of Levinoff et al. (2006), Andrés et al. (2008), Kubo-Kawai and Kawai (2010), Kessels et al. (2011), Albinet et al. (2012), Boucard et al. (2012), Kawai et al. (2012), Wang and Su (2013), Oosterman et al. (2014), Pettigrew and Martin (2014), Schroeder (2014), Kessels et al. (2015), Sylvain-Roy et al. (2015), and Rey-Mermet et al. (2018), and in the pathological impaired studies of Dwolatzky et al. (2003), Nordlund et al. (2005), Duong et al. (2006), Levinoff et al. (2006), Belleville et al. (2007), Silveri et al. (2007), Traykov et al. (2007), Wylie et al. (2007), Belleville et al. (2008), Bélanger and Belleville (2009), Pa et al. (2010), Price et al. (2010), Sinai et al. (2010), Coubard et al. (2011), Sung et al. (2012), Zheng et al. (2012), Huff et al. (2015), Kessels et al. (2015), Aurtenetxe et al. (2016), Tsai et al. (2016), and Huang et al. (2017).

Five of these cognitive aging studies, Albinet et al. (2012), Boucard et al. (2012), Pettigrew and Martin (2014), Sylvain-Roy et al. (2015), and Rey-Mermet et al. (2018), employed three or more tasks to assess one or more EFs. Albinet et al. (2012) examined updating ability between CH young and older adults with the random number generation (Baddeley, 1998) spatial running span (Morris and Jones, 1990; Albinet et al., 2012; Boucard et al., 2012), and verbal running span tasks (Morris and Jones, 1990; Albinet et al., 2012; Boucard et al., 2012) and reported no age effect when applying only the random number generation task. Boucard et al. (2012) assessed shifting ability between CH young, middle-aged, and older adults with the digit number-letter task (Rogers and Monsell, 1995), dimension-switching task (Rogers and Monsell, 1995; Monsell and Mizon, 2006; Albinet et al., 2012), and plus-minus task (Jersild, 1927; Spector and Biederman, 1976; Miyake et al., 2000b) and found no age effect with the plus-minus task in the middle-aged and older adult groups. Pettigrew and Martin (2014) employed four tasks to assess inhibitory ability between young and older adults—the flanker task (Eriksen and Eriken, 1974), the Stroop task, a nonverbal Stroop task, and the picture-word interference task. They reported no age effect with the picture-word interference task only. Rey-Mermet et al. (2018) used 11 tasks—the Antisaccade task (Hallett, 1978; Roberts et al., 1994), color Stroop (Stroop, 1935), number Stroop (Salthouse and Meinz, 1995), arrow flanker (Eriksen and Eriken, 1974; Unsworth and Spillers, 2010), letter flanker (Eriksen and Eriken, 1974; Friedman and Miyake, 2004), Simon (Simon, 1969), stop-signal (Logan et al., 1984; Williams et al., 1999), global-local (Hübner and Malinowski, 2002), positive compatibility (Eimer and Schlaghecken, 1998; Schlaghecken et al., 2012), negative compatibility (Eimer and Schlaghecken, 1998; Schlaghecken et al., 2012), and the n-2 repetition costs in task switching task (Mayr and Keele, 2000), to study age-related inhibitory ability. A decline was observed using all except the n-2 repetition costs in task switching tasks. Sylvain-Roy et al. (2015) employed the left–right (Belleville et al., 2008), number–letter (Rogers and Monsell, 1995), and the plus-minus task for shifting ability, and five updating tasks, the alpha span (Belleville et al., 1998; Craik et al., 2018), keep track (Yntema, 1963), letter updating (Sylvain-Roy et al., 2015), tone-monitoring (Larson et al., 1988; Miyake et al., 2000b), and reading span (Daneman and Carpenter, 1980). They reported age effect with all except the plus-minus and letter updating tasks. These findings may suggest that the tasks that did not detect a significant decline in their intended EF ability were not sensitive enough or tapped into different cognitive processes. This highlights the importance of carefully selecting a task or applying more than one task to confidently assess a particular ability.

For instance, the random number generation task requires inhibition and updating ability for successful completion, the latter of which showed a performance deficit. Also, the requirements of the plus-minus task where participants complete three conditions: (i) to add a specific number to every number presented, (ii) subtract a specific number from every number presented, and (iii) alternate between adding and subtracting a specific number, might be more familiar to participants, i.e., basic math, than typical EF tasks. For instance, the application of simple math, i.e., subtraction and addition, is an automated cognitive process, therefore reducing the occurrence of cognitive deficits. Similarly, it is unknown if the participants were able to complete the simple math sum successfully prior. Hence, the deficit reported may be due, in part, to forgetting how to perform the math.

Further, Pettigrew and Martin (2014) observed a near-ceiling effect with the accuracy performance (97.8% mean) of the picture-word interference task with marginal RT, suggesting it was easy to complete. Comparatively, the letter updating task may not be efficient at detecting updating deficits. Hence, it is important to note that tasks assessing the same cognitive domain may report differently due to variations in the cognitive requirements or task difficulty. Therefore, the absence of an age effect on a specific task cannot be taken to indicate the cognitive domain is unimpaired.

Similarly, in the pathological impairment studies, Silveri et al. (2007) assessed shifting ability with part B of the TMT, visual elevator task (Robertson et al., 2001), and WCST on CH older adults, aMCI, non-aMCI, and mixed MCI participants. They reported performance deficits in the mixed MCI group with all the tasks but only in the aMCI group with the visual elevator task. This may indicate that the visual elevator task used an additional cognitive process, which had declined in aMCI but was not detected by the other two, highlighting the issue of task purity.

The systematic variation of tasks may also account for how well a task detects an effect. For instance, easy tasks may probably not detect a decline due to a ceiling effect with all participants performing too well. Whereas a hard task may detect a false positive, in that participants find the task too difficult to understand and/or hard to complete, causing a floor effect. Therefore, the absence of an effect with a specific task cannot be taken to indicate the cognitive domain is unimpaired. To conclude, these findings highlight the importance of carefully selecting tasks and strongly suggest that the application of more than one or two tasks in the assessment of an EF is advantageous to confidently assess an ability, as the absence of an effect on a specific task cannot be taken to indicate the cognitive domain is unimpaired. However, it is important to note that tasks assessing the same cognitive domain may report differently due to variations in their cognitive requirements.

### Outcome measures

Many of the EF tasks employed by the studies reviewed compared performance between two task conditions, such as congruent vs. incongruent, typically reporting the relative task cost measure or, in some instances, a ratio (Sinai et al., 2010; Oosterman et al., 2014; Rey-Mermet et al., 2018; Caillaud et al., 2020). However, not all researchers used comparison measures, possibly because this was not their intended outcome measure. Instead, they just reported the findings from one half of a task, such as part B of the HSCT (Wang and Su, 2013) or the TMT (please see Supplementary material for such studies). Granted, this latter outcome measure assesses the intended EF ability of the mentioned tasks, i.e., response inhibition and set-shifting, respectively. Nevertheless, it does not take into consideration the overlapping supplementary cognitive abilities used in both parts of the task, which are eliminated when using the cost measure. For example, if participants spent longer completing part B, they may have also spent longer completing part A, suggesting no significant difference in the cost measure.

When analyzing and comparing study outcomes, it is important to consider the outcome measure being employed, as the reporting of performance deficits is dependent on it. We recommend all outcome measures should be reported for comprehensive coverage of cognitive outcomes.

### Physical health

The level of physical health of participants in the examination of EFs is also important. For example, physically active groups have been shown to perform better in EF tasks in CH older adults (Hillman et al., 2006; Boucard et al., 2012; see Zheng et al., 2022 for a systematic review), aMCI participants (Tsai et al., 2016), and AD patients (Oussama et al., 2022). Unfortunately, as this is rarely measured it could not be an exclusion criterion for the current review, though we would strongly encourage researchers to include a simple measure of physical health in future studies.

In more detail, the inhibitory assessment findings from Hillman et al. (2006) reported with the use of the flanker task (Eriksen and Eriken, 1974) that better physical activity in both CH young and older adults was related to faster performance during the incongruent and congruent conditions of the task and better accuracy in the incongruent condition. The Boucard et al. (2012) study reported, in addition to age effects across their three groups of participants groups of young, middle-aged (young-old), and older adults with the Stroop task, that the more active subgroups of all three performed superior. The more active young and older adults outperformed their sedentary counterparts in the Simon task, but the reverse was observed in the middle-aged group. With the random number generation task (Audiffren et al., 2009), the active young performed poorer than their sedentary counterparts, whilst the active middle-aged and older adults performed better than their sedentary counterparts. Likewise, physical activity further contributed to updating performance deficits with the verbal running span task. The active young and middle-aged participants outperformed their sedentary equivalents, but not the older adults. Still, age-associated updating effects without any physical activity interaction were reported with the utilization of the spatial running span and the n-back tasks. Hence, the verbal running span task might be sensitive to physical activity level, though physical activity did not positively contribute to shifting examination with the employment of the dimension-switching task (Rogers and Monsell, 1995; Monsell and Mizon, 2006; Albinet et al., 2012), (digit) number–letter task (Rogers and Monsell, 1995), or the plus–minus task (Jersild, 1927; Spector and Biederman, 1976; Miyake et al., 2000b).

Concerning non-CH older individuals, Tsai et al. (2016) described a link between poorer task-switching performance, particularly in the heterogeneous condition, in aMCI participants as compared to CH older adults. The study concluded that increased physical activity might reduce or aid in the prevention of further cognitive declines and progression to a form of dementia, which has been suggested to be a positive intervention in AD (Oussama et al., 2022).

Several studies have demonstrated that regular physical activity, particularly in older age, benefits and may have a protective effect against age-associated cognitive decline and, ultimately, dementia by reducing cardiovascular and metabolic markers associated with increased risk of cognitive decline (Bherer et al., 2013; Blondell et al., 2014; Murman, 2015; Pereira et al., 2019; Chen et al., 2020; Erickson et al., 2022; Lebeau et al., 2022). This links with the vascular hypothesis of AD (Scheffer et al., 2021), which suggests cardiovascular disease is one of the contributing factors to the onset of AD due to a reduction in cerebral blood flow, causing chronic cerebral hypoperfusion. Therefore, it is important to note the physical attributes and not just the educational and cognitive health of a study population when comparing results from studies assessing the same type of cognitive domain. Such characteristics may greatly affect results.

## Conclusion

To conclude, numerous tasks have been employed to examine EFs in CH adults and in individuals living with MCI and AD. This heterogeneity of approaches provides a strong basis for understanding which variants of paradigms are likely to reveal deficits in differing populations, but they also produce apparently conflicting evidence. This, in part, results from the huge variation in approaches adopted across studies, and we feel it is time that researchers and reviewers prioritize this issue by drawing on existing evidence when designing or publishing studies. Specifically, researchers should, by default, use variants of tasks that have been established as being effective in the target (or analogous) populations and should avoid arbitrary changes in the configurations of the paradigms, which includes matching as far as possible established parameters (stimuli, presentation times, response formats, etc.). If they wish to assess the value of a different version of a task, then they should ideally compare it to a standard baseline version of the task to meaningfully assess the value of the variation. A negative result from a task variant previously *well-established as ineffective* in the target group or where many parameters are varied *adds very little, if anything, to the field*. Reviewers looking at submissions should expect a clear rationale for any task variations. This is not an argument against innovation or change, but simply that such innovation should be more strongly grounded in previous findings than is often the case.

More specifically, the heterogeneity of task demands across studies could mean a fair comparison of the performance outcomes may not be made due to the different or additional cognitive processes required for their completion. Furthermore, the inconsistent results for an EF, as observed with the use of multiple tasks on the same study population, reporting conflicting results shows the great importance of task sensitivity as well as the inclusion of several tasks in assessing an ability during research. Nonetheless, while the same task might have been used by some researchers, inconsistencies in the methodology, stimuli, and outcome measures were also observed. Such irregularities would be reduced if EF tasks were standardized, and thus, we recommend this, as well as the implementation of tests in various languages. This review highlighted the importance of carefully selecting tasks and strongly suggested that the application of more than one or two tasks in the assessment of an EF is advantageous to confidently assess an ability, as the absence of an effect on a specific task cannot be taken to indicate the cognitive domain is unimpaired. However, it is important to note that tasks assessing the same cognitive domain may report differently due to variations in their cognitive requirements.

Another finding was with the cognitively impaired participant populations. The level of pathology was shown to affect the reported result in several studies. However, particularly with the participants living with MCI, it is not usually known what subtype, i.e., aMCI, mixed MCI, or severity, i.e., early- vs. late-stage, the group of participants is comprised of as they are normally classified as MCI. Therefore, the performance deficit observed due to the variation of a condition should also be considered in such studies, and wherever possible, authors should seek to clarify the characteristics of such groups. An adequate participant sample size is required, and the heterogeneity of such groups should be considered when determining the power of the analysis of a study.

One limitation of this review was that it failed to be comprehensive and systemic due to methodological shortcomings. Consequently, several studies may not have been included in the list of studies captured, and the tasks observed to be most frequently employed for each EF in the cognitive aging and cognitive impairment studies may differ. Nevertheless, we believe that roughly 80% of the existing literature on the topic was screened. A second limitation was not addressing the variation in age ranges used by different researchers as their young, middle-aged, and older adults. In comparing some studies, it can be seen that there are quite large differences in what population is considered young adults, i.e., a mean of 20.0 years (SD 1.4) (Bherer et al., 2006) vs. 29.2 years (SD 4.1) (Gamboz et al., 2009) and an overlap in the middle-aged and older adult age range. For instance, Kato et al. (2016) had a middle-aged group of an average of 64.8 years (SD 3.0) and older adults aged 73.9 (SD 2.6), whilst in Laguë-Beauvais et al. (2015), older adults averaged 63.47 years (SD 3.67). This will affect the assessment of cognition (e.g., MMSE vs. EF tasks) as the cross-analysis assessments are not comparable.

Despite these shortcomings, this review does highlight the many parameters that should be considered in EF studies, particularly cognitive aging and cognitive impairment studies, and should be beneficial to those currently researching in this field. The overall goal for future studies should be to reduce inconsistency in methodology and improve EF assessment across CH and impaired populations, and we further suggest the use of computer-assisted assessments to aid in these assessments and reduce human error in collecting and quantifying results (Young et al., 2022), such as with the dual-task, Stroop task, and TMT. The computerized standard versions would produce performance results faster, hopefully, more accurately, and make comparisons between studies easier and more precise.

## Author contributions

MI: Writing – review & editing, Writing – original draft. AS: Writing – review & editing. AP: Writing – review & editing.

## Footnotes

^1^The strength of the relationship between two variables in a population.

^2^The probability of the null hypothesis being correctly rejected when it is false.
