# Supplementary material for: The assessment of executive function abilities in healthy and neurodegenerative aging—A selective literature review
Source: Front Aging Neurosci. 2024 Mar 26;16:1334309. doi: 10.3389/fnagi.2024.1334309 (PMC11002121; doi:10.3389/fnagi.2024.1334309)
Supplement: Supplementary file 1 [file Data_Sheet_1.docx]

Supplementary Material

Supplementary Information for

**The Assessment of Executive Function Abilities - A Selective Review**

**Dr Mojitola I Idowu, Dr Andre J Szameitat, Dr Andrew Parton**

Corresponding Author: Dr Mojitola I Idowu

Email: Mojitola.Idowu@outlook.com

**This file includes:**

**Tables S1 to S5**

**References.**

# Tables

**Table S1. Assessing Cognitive Dual-Task capacity in Cognitive Aging, and MCI and AD sufferers.** Studies are arranged by publication year under each heading.

| **Study** | **Participants** | **Age group** | **MMSE (Mean/SD)** | **Task/Test** | **Deficit** |
| --- | --- | --- | --- | --- | --- |
| ***Cognitive Aging studies*** | | | | | |
| McCabe & Hartman (2003) | CHOA 48  CHYA 48 | 72.3 (5.9)  20.1 (2.4) | 29.4 (0.7)  NA | DT word span task | Yes  NA |
| Bherer et al. (2006) | CHOA 7M 5F  CHYA 5M 7F | 70.0 (7.0)  20.0 (1.4) | MM 56 (UNK)  NA | Auditory discrimination and visual identification task | Yes  NA |
| Maquestiaux et al. (2010) | CHOA 3M 9F  CHYA 10M 10F | 63.3 (3.0)  24.6 (2.5) | 29.2 (1.0)  NA | PRP Paradigm | Yes  NA |
| Strobach et al. (2012a) | CHOA 5M 5F  CHYA 5M 5F | 63.3 (3.4)  22.7 (3.3) | 29.8 (0.4)  NA | PRP Paradigm | Yes  NA |
| Strobach et al. (2012b) | CHOA 5M 5F  CHYA 5M 5F | 63.3 (3.4) 22.7 (3.3) | 29.8 (0.4)  NA | PRP Paradigm | Yes  NA |
| Laguë-Beauvais et al. (2015) | CHOA 6M 13F  CHYA 7M 9F | 63.47 (3.67) 23.94 (2.32) | 28.26 (0.93)  NA | Color and Letter dual-task  (PRP Paradigm) | Yes  NA |
| Ren et al. (2017) | CHOA 20  CHYA 20 | 69.0 (4.45)  21.95 (0.89) | UNK  UNK | Audiovisual temporal asynchrony integration task  (PRP Paradigm) | Yes  NA |
| Ren et al. (2018) | CHOA 15  CHYA 15 | 68.20 (4.60)  23.00 (0.93) | UNK  UNK | Audiovisual temporal asynchrony integration task  (PRP Paradigm) | Yes  NA |
| B. Wang et al. (2018) | CHOA 25  CHYA 27 | 68.8 (0.90)  23.1 (0.19) | > 24  > 24 | Audiovisual temporal asynchrony integration task  (PRP Paradigm) | Yes  NA |
| Ward et al. (2021) | CHOA 17M 16F  CHYA 19M 15F | 66.0 (5.0)  21.0 (2.0) | 28.9 (1.1)  NA | Dual- task Stroop paradigm Color-Dual | Yes  NA |
| Ward et al. (2021) | CHOA 17M 16F  CHYA 19M 15F | 66.0 (5.0)  21.0 (2.0) | 28.9 (1.1)  NA | Dual- task Stroop paradigm Lexical-Dual | Yes  NA |
| Yordanova et al. (2021) | CHOA 43M 75F  CHYA 17M 19F | 70.4 (4.2)  25.2 (2.7) | Assessed, not reported  UNK | PRP Paradigm | Yes  NA |
| ***MCI and AD studies*** | | | | | |
| Perry et al. (2000) | mAD 14  miAD 13  CHOA 30 | 70.1 (9.0)  68.2 (7.6)  67.8 (8.7) | 20.4 (2.0)  26.08 (1.6)  29.4 (0.8) | Della Sala DT | Yes  No  NA |
| Baddeley et al. (2001) – Experiment 4 | AD 26M 10F  CHOA 18M 18F  CHOY 10M 26F | 76.28 (6.33)  74.36 (8.12)  38.4 (8.79) | 19.94 (1.78)  UNK  NA | Visual search and auditory detection DT | Yes  Yes  NA |
| Calderon et al. (2001) | AD 6M 3F  CHOA 7M 10F  [DLB 8M 2F] | 71.2 (5.2)  68.3 (5.3)  72.5 (9.6) | 21.4 (2.2)  28.8 (1.0)  [20.0 (3.1)] | Della Sala DT | Yes  NA  [Yes] |
| Logie et al. (2004) – Experiment 1 | AD 4M 4F  CHOA 4M 4F  CHOY 4M 4F | 74.1 (2.4)  72.25 (6.40)  25.75 (6.00) | 21.1 (2.3)  28.9 (1.3)  NA | Baddeley’s digit recall and tracking  DT | Yes  No  NA |
| MacPherson et al. (2004) | AD 12  CHOA 12  CHOY 12 | 75.8 (8.5)  2.4 (5.4)  22.1 (3.8) | 22.0 (2.0)  UNK  NA | Della Sala DT | Yes  Yes  NA |
| Dannhauser et al. (2005) | aMCI 5M 5F  CHOA 4M 10F | 72.0 (UNK)  68.0 (UNK) | 24.5 (1.5)  28.3 (1.6) | Visual and auditory processing paradigm | Yes  NA |
| Nordlund et al. (2005) | MCI 35  CHOA 112 | 64.0 (8.2)  67.0 (5.5) | 28.5 (1.5)  29.3 (1.1) | Baddeley’s digit recall and tracking DT | No  NA |
| Lopez et al. (2006) | mixMCI 13M 15F  aMCI 6M 4F  CHOA 142M 232F | 79.7 (5.7)  79.9 (3.4)  79.5 (3.7) | 3MSE 88.2 (7.3)  92.6 (6.2)  96.0 (12.3) | Baddeley’s digit recall and tracking DT | Yes  No  NA |
| Sebastian et al. (2006) | AD 8M 19F  CHOA 7M 20F  CHOY 3M 27F | 73.70 (4.35)  72.26 (4.24)  18.60 (1.35) | 20.37 (2.20)  27.56 (2.12)  NA | Della Sala DT | Yes  No  NA |
| MacPherson et al. (2007) | AD 5M 10F  CHOA 10M 10F  CHOY 10M 10F | 75.0 (8.2)  70.8 (4.1)  26.0 (4.9) | 22.1 (1.8)  UNK  NA | Baddeley’s digit recall and tracking DT | Yes  No  NA |
| Silveri et al. (2007) | mixMCI 8  naMCI 12  aMCI 13  CHOA 21 | 74.68 (3.77)  66.87 (5.46)  73.85 (6.49)  70.62 (6.27) | 26.00 (1.41)  27.00 (2.67)  26.54 (1.98)  29.05 (0.97) | Test for Everyday Attention DT | Yes  No  No  NA |
| Kaschel et al. (2009) | AD 12M 10F  CHOA 9M 15F  [D 21M 22F] | 65.7 (6.1)  64.5 (8.3) [62.1 (6.6)] | 21.5 (3.3)  28.5 (1.3)  [29.1 (0.8)] | Baddeley’s digit recall and tracking DT | Yes  NA  [No] |
| Lonie et al. (2009) | mAD 3M 7F  aMCI 16M 17F  CHOA 8M 13F  [D 3M 14F] | 73.6 (UNK)  73.1 (UNK)  69.5 (UNK)  [73.3 (UNK)] | 25.0 (2.3)  28.4 (1.6)  29.1 (0.7)  [28.6 (1.5)] | Baddeley’s digit recall and tracking DT | No  No  NA  [No] |
| Della Sala et al. (2010) | AD 4M 4F  CHOA 4M 4F | 74.1 (2.4)  72.3 (6.4) | 21.1 (2.3)  UNK | Della Sala DT | Yes  NA |
| S. E. Price et al. (2010) | aMCI 8M 25F  CHOA 9M 24F | 77.61 (7.20)  75.52 (6.17) | 27.4 (1.4)  29.0 (0.9) | Test for Everyday Attention DT | No  NA |
| Foley et al. (2011) | AD 23M 27F  MCI 18M 31F  CHOA 22M 28F | 71.40 (7.08)  69.43 (6.74)  72.56 (7.85) | 19.32 (4.14)  27.04 (1.74)  UNK | Della Sala DT | Yes  No  NA |
| Clément et al. (2013) | LMCI 5M 7F  HMCI 5M 7F  CHOA 6M 8F | 67.21 (6.80)  68.50 (10.82)  68.33 (6.91) | 27.00 (1.81)  28.92 (1.68)  29.29 (1.14) | Alphanumeric equation task and visual detection DT | Yes  Yes  NA |
| Foley et al. (2013) | AD 23M 27F  CHOA 22M 28F | 71.40 (7.08)  72.56 (7.85) | 19.32 (4.14)  NA | Della Sala DT | Yes  NA |
| Makizako et al. (2013) | aMCI 21M 15F  CHOA 26M 36F | 76.2 (7.2)  74.0 (6.1) | 27.1 (1.8)  27.0 (2.0) | Visual stimuli and cognitive test DT | Yes  NA |

3MSE - modified Mini-Mental State Examination, a - amnestic, AD - Alzheimer’s disease, CHOA - Cognitively healthy older adult, CHYA - Cognitively healthy young adult, HMCI - high cognition MCI, LMCI - low cognition MCI, MCI - Mild Cognitive Impairment, m - mild, mi - minimal, MMSE - Mini-Mental State Examination, mix – mixed, mo - moderate, MM - modified extended Mini-Mental State Examination, NA - non applicable, UNK – unknown.

**Table S2. Assessing Inhibition** **ability in Cognitive Aging, and MCI and AD sufferers**

| **Study** | **Participants** | **Age group** | **MMSE (Mean/SD)** | **Task/Test** | **Deficit** |
| --- | --- | --- | --- | --- | --- |
| ***Cognitive Aging studies*** | | | | | |
| Nielson et al. (2002) **–** Part 1 | CHOA 4M 4F  CHYOA 1M 8F  CHMA 3M 4F  CHYA 6M 4F | 75.1 (UNK)  68.9 (UNK)  43.3 (UNK)  25.5 (UNK) | > 26  > 26  > 26  NA | Go/No-Go | Yes  No  No  NA |
| Langenecker & Nielson (2003) | CHOA 3M 8F  CHYA 4M 7F | 72.80 (3.46)  28.09 (4.11) | > 26  > 26 | Go/No-Go | No  NA |
| Langenecker et al. (2004) | CHOA 5M 8F  CHYA 6M 7F | 71.1 (5.4)  26.3 (5.5) | 28.4 (1.56)  NA | Stroop | Yes  NA |
| Nielson et al. (2004) | CHOA 6M 8F  CHYA 8M 6F | 71.1 (4.3)  29.7 (8.3) | 28.6 (1.5)  NA | Go/No-Go | Yes  NA |
| Colcombe et al. (2005) | gCHOA 6M 8F  pCHOA 10M 10F  CHYA 12M 8F | 67.56 (UNK)  67.37 (UNK)  23.50 (UNK) | > 26  > 26  NA | Flanker | Yes  No  NA |
| Bherer et al. (2006) | CHOA 7M 5F  CHYA 5M 7F | 70.0 (7.0)  20.0 (1.4) | MM 56 (UNK)  NA | Stroop | Yes  NA |
| Keightley et al. (2006) | CHOA 30  CHYA 30 | 72.5 (7.8)  25.7 (5.1) | 28.8 (0.9)  29.7 (0.5) | Stroop | Yes  NA |
| Jennings et al. (2007) | CHOA 35M 28F  CHYA 25M 35F | 69.14 (SE 0.83)  19.20 (SE 0.12) | 29.21 (SE 0.12)  NA | Flanker (Attentional network task) | Yes  NA |
| Langenecker et al. (2007) | CHOA 11  CHYA 11 | 72.8 (3.5)  28.1 (4.1) | 29.4 (0.8)  29.3 (0.7) | Go/No-Go | Yes  NA |
| Andrés et al. (2008) – Experiment 1 | CHOA 30  CHYA 30 | 73.8 (5.7)  20.0 (1.5) | 28.46 (1.13)  NA | Stroop | Yes  NA |
| Andrés et al. (2008) – Experiment 1 | CHOA 30  CHYA 30 | 73.8 (5.7)  20.0 (1.5) | 28.46 (1.13)  NA | Negative Priming | No  NA |
| Andrés et al. (2008) – Experiment 2 | CHOA 43  CHYA 45 | 68.4 (9.4)  24.3 (4.5) | 29.1 (1.3)  NA | Stop-signal | Yes  NA |
| Andrés et al. (2008) – Experiment 2 | CHOA 43  CHYA 45 | 68.4 (9.4)  24.3 (4.5) | 29.1 (1.3)  NA | Negative Priming | No  NA |
| Damoiseaux et al. (2008) | CHOA 9M 13F  CHYA 5M 5F | 70.73 (6.0)  22.80 (2.3) | 28.73 (1.4)  29.50 (0.5) | Stroop | Yes  NA |
| Clarys et al. (2009) | CHOA 44  CHYA 44 | 70.75 (6.54)  24.07 (3.45) | > 27  NA | Stroop | Yes  NA |
| Gamboz et al. (2009) | CHOA 40  CHYA 40 | 67.8 (5.0)  29.2 (4.1) | 29.5 (0.8)  NA | Stop-signal | Yes  NA |
| Kubo-Kawai & Kawai (2010) | CHOA 9M 6F  CHYA 6M 12F | 70.4 (UNK)  25.3 (UNK) | ≥ 24  NA | Simon | Yes  NA |
| Kubo-Kawai & Kawai (2010) | CHOA 9M 6F  CHYA 6M 12F | 70.4 (UNK)  25.3 (UNK) | ≥ 24  NA | Simon (Go/no-go version) | No  NA |
| Maquestiaux et al. (2010) | CHOA 3M 9F  CHYA 10M 10F | 63.3 (3.0)  24.6 (2.5) | 29.2 (1.0)  UNK | Modified Stroop | Yes  NA |
| Morrone et al. (2010) | CHOA 12M 18F  CHYA 10M 20F | 70.0 (3.32)  24.5 (2.81) | 29.5 (0.62)  NA | Hayling | Yes  NA |
| Morrone et al. (2010) | CHOA 12M 18F  CHYA 10M 20F | 70.0 (3.32)  24.5 (2.81) | 29.5 (0.62)  NA | Stroop | Yes  NA |
| Salthouse (2010) – Study 1 | CHOA 114  CHMA 89  CHYA 62 | 72.6 (8.9)  51.2 (5.0)  27.0 (6.0) | > 24  > 24  > 24 | Flanker | Yes  Yes  NA |
| Salthouse (2010) – Study 1 | CHOA 114  CHMA 89  CHYA 62 | 72.6 (8.9)  51.2 (5.0)  27.0 (6.0) | > 24  > 24  > 24 | Flanker - letter | Yes  Yes  NA |
| Vallesi et al. (2010) | CHOA 9M 11F  CHYA 8M 12F | 73.0 (UNK)  26.0 (UNK) | 28.5 (UNK)  NA | Letter-Number Go/No-Go | Yes  NA |
| Vallesi et al. (2010) | CHOA 9M 11F  CHYA 8M 12F | 73.0 (UNK)  26.0 (UNK) | 28.5 (UNK)  NA | Number Go/No-Go | Yes  NA |
| Albinet et al. (2012) | CHOA 17M 22F  CHYA 11M 17F | 71.2 (4.4)  22.7 (3.3) | 28.4 (1.4)  NA | Stroop | Yes  NA |
| Albinet et al. (2012) | CHOA 17M 22F  CHYA 11M 17F | 71.2 (4.4)  22.7 (3.3) | 28.4 (1.4)  NA | Stop-signal | Yes  NA |
| Albinet et al. (2012) | CHOA 17M 22F  CHYA 11M 17F | 71.2 (4.4)  22.7 (3.3) | 28.4 (1.4)  NA | Random Number Generation, Adjacency | Yes  NA |
| Boucard et al. (2012) | acCHOA 7M 8F  seCHOA 7M 8F  acCHYOA 7M 8F seCHYOA 7M 8F acCHYA 15M 17F  seCHYA 15M 16F | 73.4 (2.4)  75.4 (3.4)  66.3 (3.0)  66.3 (3.3)  21.9 (1.9)  22.0 (2.7) | 29.2 (0.8)  28.9 (1.0)  29.1 (0.8)  29.1 (1.0)  NA  NA | Random Number Generation, Adjacency | Yes  Yes  Yes  Yes  NA  NA |
| Boucard et al. (2012) | acCHOA 7M 8F  seCHOA 7M 8F  acCHYOA 7M 8F seCHYOA 7M 8F acCHYA 15M 17F  seCHYA 15M 16F | 73.4 (2.4)  75.4 (3.4)  66.3 (3.0)  66.3 (3.3)  21.9 (1.9)  22.0 (2.7) | 29.2 (0.8)  28.9 (1.0)  29.1 (0.8)  29.1 (1.0)  NA  NA | Simon | Yes  Yes  Yes  Yes  NA  NA |
| Boucard et al. (2012) | acCHOA 7M 8F  seCHOA 7M 8F  acCHYOA 7M 8F seCHYOA 7M 8F acCHYA 15M 17F  seCHYA 15M 16F | 73.4 (2.4)  75.4 (3.4)  66.3 (3.0)  66.3 (3.3)  21.9 (1.9)  22.0 (2.7) | 29.2 (0.8)  28.9 (1.0)  29.1 (0.8)  29.1 (1.0)  NA  NA | Stroop | Yes  Yes  Yes  Yes  NA  NA |
| Endrass et al. (2012) | CHOA 11M 11F  CHYA 10M 11F | 69.1 (UNK)  22.0 (UNK) | 29.1 (0.9)  NA | Modified Flanker | Yes  NA |
| Hsieh et al. (2012) | CHOA 9M 7F  CHYA 6M 10F | 64.63 (4.13)  20.44 (1.71) | 29.56 (0.63)  29.69 (0.70) | Flanker (PRO-bias) | Yes  NA |
| Hsieh et al. (2012) | CHOA 9M 7F  CHYA 6M 10F | 64.63 (4.13)  20.44 (1.71) | 29.19 (0.83)  29.63 (0.50) | Flanker (ANTI -bias) | Yes  NA |
| Hsieh & Fang (2012)  – Experiment 1 | CHOA 9M 7F  CHYA 6M 10F | 64.13 (2.47)  21.06 (1.61) | 29.56 (0.63)  29.69 (0.70) | Flanker (PRO-bias) | No  NA |
| Hsieh & Fang (2012)  – Experiment 2 | CHOA 9M 7F  CHYA 6M 10F | 64.19 (5.72)  21.19 (2.20) | 29.06 (0.93)  29.63 (0.62) | Flanker (non-bias) | No  NA |
| Hsieh & Fang (2012)  – Experiment 3 | CHOA 9M 7F  CHYA 6M 10F | 70.0 (UNK)  22.6 (UNK) | 29.19 (0.83)  29.63 (0.50) | Flanker (ANTI -bias) | No  NA |
| Kawai et al. (2012) | CHOA 13M 2F  CHYA 8M 5F | 70.0 (UNK)  22.6 (UNK) | 27.7 (UNK)  NA | Flanker | No  NA |
| Kawai et al. (2012) | CHOA 13M 2F  CHYA 8M 5F | 75.0 (7.2)  30.0 (3.9) | 27.7 (UNK)  NA | Simon | Yes  NA |
| Mayas et al. (2012) | CHOA 7M 11F  CHYA 7M 11F | 75.0 (7.2)  30.0 (3.9) | 29.44 (0.70)  29.44 (0.70) | Stroop | Yes  NA |
| Mayas et al. (2012) | CHOA 7M 11F  CHYA 7M 11F | 78.75 (3.07)  69.21 (2.50)  26.53 (3.90) | 29.44 (0.70)  29.44 (0.70) | Negative Priming | Yes  NA |
| Wang & Su (2013) | CHOA 16M 16F  CHOM 21M 21F  CHYA 16M 16F | 78.75 (3.07)  69.21 (2.50)  26.53 (3.90) | > 27  > 27  > 27 | Hayling part B | Yes  Yes  NA |
| Wang & Su (2013) | CHOA 7M 11F  CHOM 7M 11F  CHYA 7M 11F | 72.8 (7.3)  22.2 (2.3) | > 27  > 27  > 27 | Stroop | Yes  No  NA |
| Aisenberg et al. (2014) – Experiment 1 | CHOA 15  CHYA 15 | 68.81 (5.25)  19.41 (1.67) | 29.3 (UNK)  NA | Simon | Yes  NA |
| Amer & Hasher (2014) –  Experiment 1 | CHOA 9M 23F  CHYA 12M 22F | 66.1 (11.8)  24.0 (4.0) | 29.09 (1.06)  NA | Stroop | Yes  NA |
| Oosterman et al. (2014) | CHOA 13M 12F  CHYA 15M 11F | 71.0 (5.0)  21.0 (3.1) | UNK  UNK | Stroop | Yes  NA |
| Pettigrew & Martin (2014) | CHOA 60  CHYA 102 | 71.0 (5.0)  21.0 (3.1) | 28.8 (1.1)  NA | Flanker | Yes  NA |
| Pettigrew & Martin (2014) | CHOA 60  CHYA 102 | 71.0 (5.0)  21.0 (3.1) | 28.8 (1.1)  NA | Stroop | Yes  NA |
| Pettigrew & Martin (2014) | CHOA 60  CHYA 102 | 71.0 (5.0)  21.0 (3.1) | 28.8 (1.1)  NA | Nonverbal Stroop task | Yes  NA |
| Pettigrew & Martin (2014) | CHOA 60  CHYA 102 | 69.61 (7.35)  20.68 (1.89) | 28.8 (1.1)  NA | Picture-word interference | No  NA |
| Tournier et al. (2014) | CHOA 31  CHYA 30 | 71.9 (5.8)  23.8 (2.7) | 28.93 (1.06)  NA | Hayling | Yes  NA |
| Aisenberg et al. (2015) – Experiment 1 | CHOA 51  CHYA 45 | 63.47 (3.67) 23.94 (2.32) | > 27  NA | Simon | Yes  NA |
| Laguë-Beauvais et al. (2015) | CHOA 6M 13F  CHYA 7M 9F | 70.9 (6.1)  23.7 (3.9) | 28.26 (0.93)  NA | Stroop | Yes  NA |
| Sylvain-Roy et al. (2015) | CHOA 28M 46F  CHYA 33M 42F | 70.9 (6.1)  23.7 (3.9) | 29.0 (1.1)  NA | Antisaccade | Yes  NA |
| Sylvain-Roy et al. (2015) | CHOA 28M 46F  CHYA 33M 42F | 69.60 (8.19)  21.82 (10.43) | 29.0 (1.1)  NA | Modified Stroop | Yes  NA |
| Agustí et al. (2017) | CHOA 21M 45F  CHYA 23M 62F | 69.60 (8.19)  21.82 (10.43) | ≥ 26  ≥ 26 | Emotional Stroop - face | Yes  NA |
| Agustí et al. (2017) | CHOA 21M 45F  CHYA 23M 62F | 68.7 (UNK)  25.0 (UNK) | ≥ 26  ≥ 26 | Emotional Stroop - word | Yes  NA |
| Coxon et al. (2016) | CHOA 9M 11F  CHYA 9M 11F | 68.63 (5.88)  21.63 (1.49) | ≥ 27  ≥ 29 | Stop-signal | Yes  NA |
| Hsieh et al. (2016) | CHOA 7M 9F  CHYA 7M 9F | 67.25 (4.59)  21.31 (1.26) | 27.19 (0.73) 28.19 (1.01) | 20% Go/80% No-Go (small demand) | Yes  NA |
| Hsieh et al. (2016) | CHOA 8M 8F  CHYA 8M 8F | 66.38 (4.43  21.31 (1.36) | 26.69 (1.10) 28.50 (0.71) | 50% Go/50% No-Go (equal demand) | Yes  NA |
| Hsieh et al. (2016) | CHOA 7M 9F  CHYA 7M 9F | 21.07 (3.28) [For 14]  61.43 (9.85) [For 15] | 27.06 (1.14) 28.63 (0.60) | 80% Go/20% No-Go (high demand) | Yes  NA |
| Crawford et al. (2017) | CHOA 15  CHYA 16 | 21.07 (3.28) [For 14]  61.43 (9.85) [For 15] | UNK  NA | Antisaccade | Yes  NA |
| Crawford et al. (2017) | CHOA 15  CHYA 16 | 21.07 (3.28) [For 14]  61.43 (9.85) [For 15] | UNK  NA | Memory-guided  Antisaccade | Yes  NA |
| Crawford et al. (2017) | CHOA 15  CHYA 16 | 69.4 (2.9)  22.5 (2.6) | UNK  NA | Go/No-Go Antisaccade | Yes  NA |
| Rey-Mermet et al. (2018) | CHOA 124  CHYA 108 | 69.4 (2.9)  22.5 (2.6) | > 26  > 26 | Antisaccade | Yes  NA |
| Rey-Mermet et al. (2018) | CHOA 124  CHYA 108 | 69.4 (2.9)  22.5 (2.6) | > 26  > 26 | Color Stroop | Yes  NA |
| Rey-Mermet et al. (2018) | CHOA 124  CHYA 108 | 69.4 (2.9)  22.5 (2.6) | > 26  > 26 | Number Stroop | Yes  NA |
| Rey-Mermet et al. (2018) | CHOA 124  CHYA 108 | 69.4 (2.9)  22.5 (2.6) | > 26  > 26 | Arrow flanker | Yes  NA |
| Rey-Mermet et al. (2018) | CHOA 124  CHYA 108 | 69.4 (2.9)  22.5 (2.6) | > 26  > 26 | Letter flanker | Yes  NA |
| Rey-Mermet et al. (2018) | CHOA 124  CHYA 108 | 69.4 (2.9)  22.5 (2.6) | > 26  > 26 | Simon | Yes  NA |
| Rey-Mermet et al. (2018) | CHOA 124  CHYA 108 | 69.4 (2.9)  22.5 (2.6) | > 26  > 26 | Stop-signal | Yes  NA |
| Rey-Mermet et al. (2018) | CHOA 124  CHYA 108 | 69.4 (2.9)  22.5 (2.6) | > 26  > 26 | Global | Yes  NA |
| Rey-Mermet et al. (2018) | CHOA 124  CHYA 108 | 69.4 (2.9)  22.5 (2.6) | > 26  > 26 | Local | Yes  NA |
| Rey-Mermet et al. (2018) | CHOA 124  CHYA 108 | 69.4 (2.9)  22.5 (2.6) | > 26  > 26 | Positive compatibility | Yes  NA |
| Rey-Mermet et al. (2018) | CHOA 124  CHYA 108 | 69.4 (2.9)  22.5 (2.6) | > 26  > 26 | Negative compatibility | Yes  NA |
| Rey-Mermet et al. (2018) | CHOA 124  CHYA 108 | 66.47 (6.73)  20.11 (1.31) | > 26  > 26 | n-2 repetition costs in task switching | No  NA |
| Dupart et al. (2018) | CHOA 7M 31F  CHYA 8M 30F | 70.42 (7.32)  19.00 (1.14) | 28.97 (1.35)  NA | Emotional Hayling | Yes  NA |
| Waring et al. (2019) | CHOA 17M 19F  CHYA 24M 20F | 70.42 (7.32)  19.00 (1.14) | 29.17 (1.06)  NA | Emotional Go/No-Go | Yes  NA |
| Waring et al. (2019) | CHOA 17M 19F  CHYA 24M 20F | 79.6 (4.8)  68.0 (3.5)  21.5 (3.2) | 29.17 (1.06)  NA | Color word interference | Yes  NA |
| Glisky et al. (2020) | CHOA 58M 66F  CHOA 48M 72F  CHYA 24M 20F | 79.6 (4.8)  68.0 (3.5)  21.5 (3.2) | 29 (range = 25-30)  29 (range = 25-30)  NA | Simon | No  No  NA |
| Glisky et al. (2020) | CHOA 58M 66F  CHOA 48M 72F  CHYA 24M 20F | 65.1 (4.6)  20.3 (1.7) | 29 (range = 25-30)  29 (range = 25-30)  NA | Stroop | Yes  Yes  NA |
| Servant & Evans (2020) | CHOA 4M 16F  CHYA 3M 17F | 68.41 (5.89)  19.12 (1.68) | 29.1 (1.1)  28.6 (1.2) | Flanker | Yes  NA |
| S. E. Williams et al. (2020) – Study 1 | CHOA 17M 24F  CHYA 17M 23F | 69.41 (6.49)  18.70 (0.76) | 29.29 (0.96)  NA | Emotional Go/No-Go | No  NA |
| S. E. Williams et al. (2020) – Study 2 | CHOA 17M 24F  CHYA 17M 23F | 70.30 (5.69)  19.22 (1.47) | 29.05 (1.12)  NA | Emotional Go/No-Go | No  NA |
| S. E. Williams et al. (2020) – Study 3 | CHOA 17M 24F  CHYA 17M 23F | 73.45 (7.25)  71.73 (5.10)  22.64 (2.15)  22.45 (2.42) | 28.95 (1.01)  NA | Emotional Go/No-Go | Yes  NA |
| Kamboureli & Economou (2021) | HA CHOA 4M 7F  LA CHOA 5M 6F  HA CHYA 4M 7F  LA CHYA 7M 4F | 73.45 (7.25)  71.73 (5.10)  22.64 (2.15)  22.45 (2.42) | 4M 7F  5M 6F  4M 7F  7M 4F | Stroop | Yes  NA  Yes  NA |
| Kamboureli & Economou (2021) | HA CHOA 4M 7F  LA CHOA 5M 6F  HA CHYA 4M 7F  LA CHYA 7M 4F | 70.4 (4.2)  25.2 (2.7) | 4M 7F  5M 6F  4M 7F  7M 4F | Emotional Stroop | Yes  NA  Yes  NA |
| Yordanova et al. (2021) | CHOA 43M 75F  CHYA 17M 19F | 67.00 (4.55)  50.25 (5.15)  26.40 (4.30)  17.40 (1.29) | Assessed, not reported  UNK | Stroop | Yes  NA |
| Belghali et al. (2022) | CHOA 15M 11F  CHMA 14M 13F  CHYA 12M 13F  CHAD 12M 13F | 74.04 (UNK)  21.48 (UNK) | ≥ 27  ≥ 27  ≥ 27  ≥ 27 | Stroop | Yes  No  NA  NA |
| Burca et al. (2022) | CHOA 51  CHYA 50 | 74.56 (8.35)  21.48 (4.03) | 29.00 (1.30)  NA | Stroop | Yes  NA |
| ***MCI and AD studies*** | | | | | |
| Perry et al. (2000) | mAD 14  miAD 13  CHOA 30 | 70.1 (9.0)  68.2 (7.6)  67.8 (8.7) | 20.4 (2.0)  26.08 (1.6)  29.4 (0.8) | Stroop | Yes  Yes  NA |
| Calderon et al. (2001) | AD 6M 3F  CHOA 7M 10F  [DLB 8M 2F] | 71.2 (5.2)  68.3 (5.3)  72.5 (9.6) | 21.4 (2.2)  28.8 (1.0)  [20.0 (3.1)] | Stroop | Yes  NA  [NA] |
| Collette et al. (2002) | AD 4M 22F  CHOA 4M 22F | 69.0 (7.4)  68.8 (7.2) | 19.3 (4.2)  UNK | Go/No-Go | Yes  NA |
| Collette et al. (2002) | AD 4M 22F  CHOA 4M 22F | 69.0 (7.4)  68.8 (7.2) | 19.3 (4.2)  UNK | Hayling | Yes  NA |
| Collette et al. (2002) | AD 4M 22F  CHOA 4M 22F | 69.0 (7.4)  68.8 (7.2) | 19.3 (4.2)  UNK | Stroop | Yes  NA |
| Dwolatzky et al. (2003) | mAD 13M 16F  MCI 17M 13F  CHOA 13M 26F | 80.55 (4.91)  77.15 (6.43)  73.41 (8.00) | 24.17 (3.25)  27.63 (1.54)  29.03 (1.11) | Go/No-Go | Yes  Yes  NA |
| Dwolatzky et al. (2003) | mAD 13M 16F  MCI 17M 13F  CHOA 13M 26F | 80.55 (4.91)  77.15 (6.43)  73.41 (8.00) | 24.17 (3.25)  27.63 (1.54)  29.03 (1.11) | Stroop | NA  Yes  NA |
| Shafiq-Antonacci et al. (2003) | AD 15M 20F  CHOA 79M 166F | 70.9 (9.4)  62.8 (8.6) | < 28  ≥ 28 | Antisaccade | Yes  NA |
| Amieva et al. (2004) | revAD 6M 16F  revCHOA 6M 16F  intAD 5M 17F  intCHOA 5M 17F | 74.8 (6.3)  74.9 (6.4)  74.0 (5.1)  74.0 (4.7) | 21.4 (2.4)  27.5 (1.7)  21.1 (3.0)  27.9 (1.7) | Modified Stroop | Yes  NA  Yes  NA |
| Levinoff et al. (2004) | AD 4M 22F  CHOA 4M 22F | 74.1 (8.3)  73.0 (6.1) | 22.4 (3.1) 28.8 (1.0) | Stroop | Yes  NA |
| Crawford et al. (2005) | mAD 13M 5F  CHOA 8M 10F CHOY 8M 9F | 77.8 (4.8)  75.2 (3.8)  23.8 (UNK) | 20.9 (4.3)  29.2 (1.1)  NA | Antisaccade | Yes  Yes  NA |
| Crawford et al. (2005) | mAD 13M 5F  CHOA 8M 10F CHOY 8M 9F | 77.8 (4.8)  75.2 (3.8)  23.8 (UNK) | UNK  > 27  NA | Go/No-Go | Yes  Yes  NA |
| Nordlund et al. (2005) | MCI 35  CHOA 112 | 64.0 (8.2)  67.0 (5.5) | 28.5 (1.5)  29.3 (1.1) | Picture Stroop | Yes  NA |
| Nordlund et al. (2005) | MCI 35  CHOA 112 | 64.0 (8.2)  67.0 (5.5) | 28.5 (1.5)  29.3 (1.1) | Stroop - Victoria | No  NA |
| Belleville et al. (2006) | AD 4M 8F  CHOA 4M 8F  CHOY 6M 6F | 72.5 (5.9)  72.7 (4.6)  22.0 (3.2) | 22.9 (2.0)  28.2 (1.1)  NA | Hayling | Yes  Yes  NA |
| Belleville et al. (2006) | AD 4M 8F  CHOA 4M 8F  CHOY 6M 6F | 72.5 (5.9)  72.7 (4.6)  22.0 (3.2) | 22.9 (2.0)  28.2 (1.1)  NA | Stroop | Yes  Yes  NA |
| Duong et al. (2006) | AD 39  MCI 61  CHOA 60 | 73.62 (8.94)  74.68 (6.48)  74.38 (5.74) | 29.12 (0.97)  27.20 (2.25)  22.08 (3.76) | Picture Stroop | Yes  Yes  NA |
| Duong et al. (2006) | AD 39  MCI 61  CHOA 60 | 73.62 (8.94)  74.68 (6.48)  74.38 (5.74) | 29.12 (0.97)  27.20 (2.25)  22.08 (3.76) | Stroop - Victoria | Yes  No  NA |
| Kramer et al. (2006) | AD 33  aMCI 22  CHOA 35 | 73.4 (9.2)  75.0 (6.1)  73.0 (5.3) | 25.2 (1.3)  28.5 (1.5)  29.5 (0.8) | Stroop | Yes  Yes  NA |
| Lopez et al. (2006) | mixMCI 13M 15F  aMCI 6M 4F  CHOA 142M 232F | 79.7 (5.7)  79.9 (3.4)  79.5 (3.7) | 3MSE 88.2 (7.3)  92.6 (6.2)  96.0 (12.3) | Stroop | Yes  No  NA |
| Stokholm et al. (2006) | AD 16M 20F  CHOA 12M 20F | 76.0 (5.6)  74.3 (4.2) | 25.9 (1.5)  29.3 (0.9) | Stroop | Yes  NA |
| Belleville et al. (2007) | AD 19  CHOA 29 in total  MCI 28  CHOA 29 in total | 73.42 (9.18)  72.42 (8.31)  64.76 (10.83)  66.12 (10.09) | 24.65 (3.60)  28.74 (0.93)  28.36 (1.98)  28.88 (0.99) | Hayling | Yes  NA  No  NA |
| Belleville et al. (2007) | AD 19  CHOA 29 in total  MCI 28  CHOA 29 in total | 73.42 (9.18)  72.42 (8.31)  64.76 (10.83)  66.12 (10.09) | 24.65 (3.60)  28.74 (0.93)  28.36 (1.98)  28.88 (0.99) | Stroop - Victoria | Yes  NA  No  NA |
| Traykov et al. (2007) | MCI 16M 4F  CHOA 14M 6F | 73.2 (8.0)  73.3 (7.0) | 28.95 (1.1)  29.5 (0.5) | Stroop | Yes  NA |
| Wylie et al. (2007) | MCI 8M 12F  CHOA 9M 11F | 73.0 (6.1)  71.5 (8.7) | 26.0 (2.5) 29.3 (0.8) | Stroop | No  NA |
| Wylie et al. (2007) | MCI 8M 12F  CHOA 9M 11F | 73.0 (6.1)  71.5 (8.7) | 26.0 (2.5) 29.3 (0.8) | Flanker | Yes  NA |
| Zamarian et al. (2007) | AD 6M 9F  CHOA 7M 13F  MCI 11M 7F  CHOA 5M 15F | 77.7 (5.0)  75.3 (4.0)  68.0 (6.9)  66.6 (1.5) | 21.3 (2.2)  29.1 (0.8)  27.0 (1.4)  28.8 (0.8) | Math Stroop | Yes  NA  Yes  NA |
| Zamarian et al. (2007) | AD 6M 9F  CHOA 7M 13F  MCI 11M 7F  CHOA 5M 15F | 77.7 (5.0)  75.3 (4.0)  68.0 (6.9)  66.6 (1.5) | 21.3 (2.2)  29.1 (0.8)  27.0 (1.4)  28.8 (0.8) | Color word interference | Yes  NA  Yes  NA |
| Zhang et al. (2007) | MCI 32  CHOA 32 | 73.7 (8.2)  73.5 (8.5) | 27.4 (2.0)  28.7 (1.8) | Go/No-Go | No  NA |
| Zhang et al. (2007) | MCI 32  CHOA 32 | 73.7 (8.2)  73.5 (8.5) | 27.4 (2.0)  28.7 (1.8) | Negative Priming | No  NA |
| Zhang et al. (2007) | MCI 32  CHOA 32 | 73.7 (8.2)  73.5 (8.5) | 27.4 (2.0)  28.7 (1.8) | Stroop | No  NA |
| Belleville et al. (2008) | AD 6M 7F  CHOA M 11F  MCI 8M 12F  CHOA M 15F | 73.2 (8.1)  72.8 (7.6)  66.3 (10.9)  66.2 (9.6) | 24.85 (4.0)  28.69 (0.8)  28.15 (2.1)  28.9 (0.9) | Stroop - Victoria | Yes  NA  No  NA |
| Bisiacchi et al. (2008) – Experiment 2 | AD 8M 12F  aMCI 6M 8F  CHOA 5M 9F | 77.65 (6.64)  76.36 (7.41)  78.55 (6.43) | 20.79 (1.92)  25.71 (1.59)  27.80 (1.57) | Hayling | Yes  No  NA |
| Kaufmann et al. (2008) | MCI 6  CHOA 9 | 69.8 (5.3)  68.3 (7.5) | 24.8 (1.2)  29.0 (1.2) | Numerical Stroop | Yes  NA |
| Ramsden et al. (2008) | AD 4M 11F  CHOA 6M 10F | 70.87 (5.77)  65.75 (4.23) | 20.67 (1.88)  29.06 (1.06) | Stroop | No  NA |
| Bélanger & Belleville (2009) | AD 8  MCI 18  CHOA 16  CHYA 20 | Not reported  Not reported  Not reported  Not reported | 23.5 (4.0)  27.3 (1.8)  29.2 (0.9)  NA | Hayling | Yes  Yes  Yes  NA |
| Bélanger & Belleville (2009) | AD 8  MCI 18  CHOA 16  CHYA 20 | Not reported  Not reported  Not reported  Not reported | 23.5 (4.0)  27.3 (1.8)  29.2 (0.9)  NA | Stroop | No  No  NA  NA |
| Brambati et al. (2009) | mMCI 18 5M 5F  aMCI-MD 3M 11F  aMCI-SD 5M 6F  CHOA 5M 8F | 71.5 (5.9)  72.3 (6.2)  74.9 (7.7)  75.0 (5.1) | 22.5 (2.3)  26.5 (1.8)  28.5 (1.0)  29.1 (1.2) | Stroop - Victoria | Yes  Yes  No  NA |
| C. Li et al. (2009) | AD 5M 5F  MCI 5M 4F  CHOA 4M 5F | 65.8 (6.1)  63.4 (4.6)  65.2 (7.2) | 16.7 (2.6)  26.4 (4.2)  28.8 (0.9) | Stroop | Yes  Yes  NA |
| Zhou & Jia (2009) | MCI/AD 12M 18F  MCI/SVD 36M 20F  CHOA 45M 35F | 72.1 (7.2)  67.3 (6.2)  66.9 (7.1) | 26.2 (1.1)  26.7 (2.2)  28.8 (1.1) | Stroop | Yes  Yes  NA |
| Bélanger et al. (2010) | AD 11  MCI 20  CHOA 20  CHYA 20 | 75.0 (6.4)  72.7 (6.8)  71.10 (7.5)  23.9 (4.7) | 23.4 (3.7)  27.4 (2.1)  28.8 (1.4)  NA | Stroop | Yes  Yes  Yes  NA |
| Hutchison et al. (2010) | AD (mild) 21M 17F  CHOA 24M 39F | 78.78 (5.89)  77.24 (9.80) | 28.22 (UNK)  29.19 (UNK) | Stroop | Yes  NA |
| Luks et al. (2010) | AD 4M 2F  MCI 6M 3F  CHOA 12M 10F  [CBD 1M 1F  FTD 8M 3F  PNFA 1M 1F  PSP 1M 2F  SD 6M 4F] | 63.9 (8.8) – includes all dementias  Not reported | 27.0 (0.8)  29.0 (1.0)  29.0 (0.7)  [28.0 (0.0)  27.0 (2.9)  27.0 (0.0)  27.0 (4.2)  24.0 (5.8)] | Flanker | Yes  No  NA  [Yes  Yes  No  Yes  Yes] |
| McGuinness et al. (2010) | AD 28  CHOA 75  [VaD 46] | 77.7 (6.9)  70.2 (7.9)  [75.9 (7.8)] | > 12  ≥ 28  [≥ 12] | Stroop | Yes  NA  [Yes] |
| Pa et al. (2010) | AD 6M 4F  MCI 30M 27F  CHOA 20M 20F  [ALS 5M 1F  CBD 4M 8F  FTD 17M 4F  SD 9M 5F] | 62.6 (5.4)  69.8 (9.3)  65.2 (8.9)  [62.0 (10.0)  62.9 (8.5)  60.8 (7.7)  62.1 (5.9)] | 26.0 (3.1)  28.4 (1.5)  29.8 (0.5)  [29.2 (2.0)  27.3 (2.0)  26.1 (4.4)  23.5 (6.2)] | Color word interference | Yes  Yes  NA  [No  Yes  Yes  Yes] |
| S. E. Price et al. (2010) | aMCI 8M 25F  CHOA 9M 24F | 77.61 (7.20)  75.52 (6.17) | 27.4 (1.4)  29.0 (0.9) | Color word interference | No  NA |
| Sinai et al. (2010) | MCI-able 6M 10F  MCI-cue 3M 2F  MCI-unable 2M 4F  CHOA 5M 12F | 75.5 (1.7)  77.0 (2.9)  76.5 (2.6)  75.7 (1.5) | 28.40 (0.4)  26.20 (0.8)  25.17 (0.8)  28.6 (0.4) | Stroop – Victoria | No  No  No  NA |
| Tse et al. (2010) | AD 74  CHOA 246  CHYA 32 | 75.82 (7.81)  71.77 (7.71)  20.31 (1.12) | 26.58 (2.78)  28.99 (1.36)  NA | Stroop | Yes  Yes  NA |
| Tse et al. (2010) | AD 74  CHOA 246  CHYA 32 | 75.82 (7.81)  71.77 (7.71)  20.31 (1.12) | 26.58 (2.78)  28.99 (1.36)  NA | Simon | Yes  Yes  NA |
| Ahn et al. (2011) | AD 52M 118F  aMCI 47M 52F  CHOA 56M 86F | 73.5 (7.5)  72.3 (7.2)  66.0 (7.9) | 19.3 (5.0)  26.2 (2.5)  28.7 (1.5) | Go/No-Go | Yes  Yes  NA |
| Ahn et al. (2011) | AD 52M 118F  aMCI 47M 52F  CHOA 56M 86F | 73.5 (7.5)  72.3 (7.2)  66.0 (7.9) | 19.3 (5.0)  26.2 (2.5)  28.7 (1.5) | Stroop | Yes  Yes  NA |
| Coubard et al. (2011) | AD 3M 14F  CHOA 10M 7F  CHYA 8M 10F | 78.68 (6.15)  77.65 (7.72)  25.36 (2.78) | UNK  UNK  NA | Stroop | Yes  Yes  NA |
| Gagnon & Belleville (2011) | AD 16  aMCI 13 and  md aMCI 7  CHOA 20 | 71.63 (7.27)  73.40 (6.89)  69.90 (7.93) | 23.94 (2.29)  27.95 (1.50)  28.80 (1.06) | Stroop – Victoria | Yes  No  NA |
| C. Li et al. (2011) | AD 3M 3F  CHOA 3M 5F  [VaD 4M 2F] | 68.0 (UNK)  66.0 (UNK)  66.0 (UNK) | 20.4 (UNK)  28.7 (UNK)  [20.4 (UNK)] | Stroop | Yes  NA  [Yes] |
| Yun et al. (2011) | AD 7M 10F  CHOA 11M 6F | 70.2 (8.4)  70.4 (5.7) | 16.6 (5.1)  26.8 (2.3) | Stroop | Yes  NA |
| Guerdoux et al. (2012) – Experiment 2 | AD 7M 10F  aMCI 10M 7F  CHOA 11M 6F | 75.0 (8.4)  71.0 (5.9)  72.0 (7.3) | 24.0 (1.9)  27.5 (1.6)  28.4 (1.3) | Stroop – Victoria | Yes  No  NA |
| Johns et al. (2012) | aMCI 18M 22F  CHOA 13M 19F | 72.4 (8.6)  71.8 (5.0) | 28.1 (1.4)  28.9 (1.1) | Stroop | Yes  NA |
| Johns et al. (2012) | aMCI 18M 22F  CHOA 13M 19F | 72.4 (8.6)  71.8 (5.0) | 28.1 (1.4)  28.9 (1.1) | Hayling | Yes  NA |
| Zheng et al. (2012) | aMCI 14M 20F  CHOA 18M 18F | 67.9 (6.7)  67.4 (5.0) | 28.3 (1.5)  29.5 (0.7) | Stroop | No  NA |
| Zheng et al. (2012) | aMCI 14M 20F  CHOA 18M 18F | 67.9 (6.7)  67.4 (5.0) | 28.3 (1.5)  29.5 (0.7) | Stop-signal | Yes  NA |
| Sung et al. (2012) | MCI 16  CHOA 16 | 73.0 (8.0)  70.0 (5.0) | ^1^24.87 (3.40)  ^1^26.45 (2.11) | Go/No-Go | No  NA |
| Sung et al. (2012) | MCI 16  CHOA 16 | 73.0 (8.0)  70.0 (5.0) | ^1^24.87 (3.40)  ^1^26.45 (2.11) | Stroop | Yes  NA |
| Alichniewicz et al. (2013) | aMCI 5M 18F  CHOA 8M 11F | 60.30 (9.31)  58.84 (7.41) | 28.91 (1.13)  29.32 (0.82) | Antisaccade | Yes  NA |
| Chen et al. (2013) | AD 88M 38F  aMCI 82M 38F  CHOA 68M 32F | 78.9 (5.5)  78.2 (7.7)  75.4 (7.3) | 20.2 (3.6)  26.6 (1.4)  28.4 (1.7) | Stroop | Yes  Yes  NA |
| Crawford et al. (2013) | AD 18  CHOA 18  CHYA 17  [PD 25] | 78 (4.8)  75 (3.6)  23.8 (1.7)  [63 (7.4)] | 20.9 (4.3)  29.2 (1.1)  UNK  [28.8 (1.2)] | Antisaccade | Yes  NA  No  [No] |
| Heuer et al. (2013) | AD 16M 12F  MCI 18M 18F  CHOA 49M 69F | 60.9 (1.65)  72.9 (1.12)  69.4 (0.57) | 20.64 (0.92)  28.77 (0.24)  29.54 (0.64) | Antisaccade | Yes  No  NA |
| Heuer et al. (2013) | AD 16M 12F  MCI 18M 18F  CHOA 49M 69F | 60.9 (1.65)  72.9 (1.12)  69.4 (0.57) | 20.64 (0.92)  28.77 (0.24)  29.54 (0.64) | Stroop | Yes  No  NA |
| Van Dam et al. (2013) | aMCI 4M 4F  CHOA 2M 6F | 77.6 (7.0)  74.6 (9.2) | 27.1 (1.8) 28.8 (1.4) | Modified ANT (Flanker) | Yes  NA |
| P. Wang et al. (2013) | AD 3M 4F  MCI 9M 6F  CHOA 9M 7F | 68.59 (2.9)  72.9 (1.9)  69.3 (1.8) | 21.5 (0.8)  27.0 (0.5)  29.3 (0.5) | Modified Flanker | Yes  No  NA |
| Cid-Fernández et al. (2014) | aMCI 14M 16F  CHOA 22M 41F | 69.5 (8.2)  65.9 (8.0) | 25.9 (2.4)  28.2 (1.5) | Go/No-Go | Yes  NA |
| Peltsch et al. (2014) | AD 22M 50F  aMCI 10M 12F  CHOA 9M 15F | 76.0 (8.0)  76.0 (8.0)  73.0 (6.0) | 27.0 (2.0)  27.0 (2.0)  29.0 (1.0) | Antisaccade | Yes  Yes  NA |
| Peltsch et al. (2014) | AD 22M 50F  aMCI 10M 12F  CHOA 9M 15F | 76.0 (8.0)  76.0 (8.0)  73.0 (6.0) | 27.0 (2.0)  27.0 (2.0)  29.0 (1.0) | Stroop | Yes  Yes  NA |
| Pereiro et al. (2014) | md aMCI 31  sd aMCI 31  CHOA 41 | 69.22 (9.41)  68.25 (9.89)  67.34 (9.03) | 23.87 (1.78)  27.54 (1.47)  28.58 (1.35) | Simon | Yes  Yes  NA |
| Puente et al. (2014) | MCI 7M 10F  CHOA10M 16F | 75.0 (6.3)  74.0 (5.5) | 25.9 (2.4)  28.0 (2.0) | Stroop | Yes  NA |
| Zheng et al. (2014) | aMCI 16M 34F  CHOA 19M 29F | 69.8 (6.8)  69.2 (5.1) | 27.9 (1.5)  29.5 (0.7) | Stop-signal | Yes  NA |
| El Haj, Larøi, et al. (2015) | AD 8M 23F  CHOA 10M 23F | 71.42 (5.18)  68.85 (8.21) | 21.68 (1.87)  28.00 (1.52) | Stroop | Yes  NA |
| El Haj, Antoine, & Kapogiannis (2015) | AD 8M 16F  CHOA 9M 17F | 72.08 (7.20)  72.58 (7.20) | 21.83 (1.52)  28.31 (1.28) | Stroop | Yes  NA |
| B. Y. Li et al. (2016) | MCI 15M 9F  CHOA 14M 8F | 69.27 (7.55)  69.17 (8.91) | 26.41 (2.12)  28.95 (0.95) | Stroop | No  NA |
| Mudar et al. (2016) | aMCI 9M 16F  CHOA 9M 16F | 68.5 (8.0)  65.4 (7.1) | 28.4 (1.3)  28.6 (0.5) | Go/No-Go | Yes  NA |
| Yuan et al. (2016) | aMCI 57M 62F  CHOA 42M 37F | 68.16 (6.67)  69.65 (7.60) | 26.21 (2.69)  28.21 (1.46) | Stroop | Yes  NA |
| Borella et al. (2017) | MCI 6M 9F  CHOA 7M 11F | 72.73 (5.28)  69.72 (3.20) | 27.40 (1.45)  29.50 (0.62) | Stroop | Yes  NA |
| Huang et al. (2017) | AD 11M 20F  CHOA 17M 14F | 78.9 (6.3)  76.5 (5.9) | 21.2 (3.2)  27.0 (1.2) | Stroop | Yes  NA |
| Martyr et al. (2017) | AD 18M 12F  CHOA 22M 32F  [PD 15M 18F] | 78.40 (7.41)  72.09 (6.49)  [72.21 (8.04)] | 23.10 (2.87)  28.78 (1.00)  [29.39 (1.12)] | Hayling | Yes  NA  [Yes] |
| Nguyen et al. (2017) | aMCI 8M 14F  CHOA 6M 16F | 68.68 (7.69)  65.32 (6.84) | 28.32 (1.29)  28.75 (0.50) | Go/No-Go | Yes  NA |
| Borsa et al. (2018) | aMCI 5M 2F  CHOA 5M 2F | 73.29 (6.90)  68.71 (5.09) | 27.14 (2.11)  28.42 (1.81) | Flanker (Attentional network task) | Yes  NA |
| Holden et al. (2018) | AD 8M 15F  aMCI 11M 18F  CHOA 13M 14F | 70.6 (6.1)  71.3 (7.1)  69.5 (6.1) | 23.3 (2.6) 26.4 (1.8)  28.1 (1.6) | Antisaccade – Gap | Yes  Yes  NA |
| Matías-Guiu et al. (2018) | AD 7M 12F  CHOA 9M 10F  [bvFTD 9M 10F  ALS 8M 11F] | 72.16 (8.48)  64.89 (8.81)  [71.05 (7.72)  57.89 (9.74)] | 24.26 (4.33)  29.16 (1.21)  [24.00 (4.79)  28.00 (1.63)] | Hayling | Yes  NA  [Yes  No] |
| Matías-Guiu et al. (2018) | AD 7M 12F  CHOA 9M 10F  [bvFTD 9M 10F  ALS 8M 11F] | 72.16 (8.48)  64.89 (8.81)  [71.05 (7.72)  57.89 (9.74)] | 24.26 (4.33)  29.16 (1.21)  [24.00 (4.79)  28.00 (1.63)] | Stroop | Yes  NA  [Yes  No] |
| Noiret et al. (2018) | AD 9M 11F  CHOA 9M 11F | 79.00 (5.93)  71.75 (3.71) | 21.68 (3,51)  28.80 (1.32) | Antisaccade | Yes  NA |
| Cervera-Crespo et al. (2019) | moAD 8M 8F  mAD 7M 8F  CHOA 8M 8F | 79.3 (3.16)  79.1 (6.26)  77.9 (5.14) | 22.46 (1.06)  23.81 (0.91)  28.66 (2.49) | Hayling | Yes  Yes  NA |
| Garcia-Alvarez et al. (2019) | AD 27M 30F  MCI 27M 21F  CHOA 49M 75F | 76.58 (10.31)  76.68 (10.27)  73.17 (8.60) | 21.21 (4.28)  25.96 (2.03)  28.49 (1.40) | Stroop | UNK  Yes  NA |
| Ferreira et al. (2019) | moAD 11  mAD 22  CHOA 56  [D 19] | 73.00 (UNK)  75.00 (UNK)  68.50 (UNK)  [67.00 (UNK)] | 19.00 (UNK)  22.50 (UNK)  29.00 (UNK)  [29.00 (UNK)] | Stroop | Yes  Yes  NA  [No] |
| Caillaud et al. (2020) | MCI 14M 15F  SCI 25M 42F  CHOA 9M 21F | 76.3 (5.3)  72.3 (5.1)  71.9 (5.7) | 24.3 (1.6) 24.4 (1.9) 25.2 (1.0) | Hayling | Yes  No  NA |
| Meléndez et al. (2020) | moAD 9M 16F  mAD 10M 15F  CHOA 11M 14F | 81.40 (7.70)  81.44 (4.26)  79.76 (5.18) | 16.2 (2.23)  22.2 (0.95)  29 (1.38) | Emotional Stroop – face | Yes  Yes  NA |
| Meléndez et al. (2020) | moAD 9M 16F  mAD 10M 15F  CHOA 11M 14F | 81.40 (7.70)  81.44 (4.26)  79.76 (5.18) | 16.2 (2.23)  22.2 (0.95)  29 (1.38) | Emotional Stroop – word | Yes  Yes  NA |
| Satorres et al. (2020) | AD 12M 43F  CHOA 17M 23F | 80.70 (6.66)  78.97 (5.37) | 19.09 (3.44)  28.87(1.43) | Emotional Stroop – face | Yes  NA |
| Satorres et al. (2020) | AD 12M 43F  CHOA 17M 23F | 80.70 (6.66)  78.97 (5.37) | 19.09 (3.44)  28.87(1.43) | Emotional Stroop – word | Yes  NA |
| Chehrehnegar et al. (2022) | AD 6M 14F  aMCI 13M 27F  CHOA 23M 36F | 73.52 (7.46)  68.10 (8.81)  62.55 (6.78) | 22.04 (3.27)  25.62 (3.22)  28.16 (1.52) | Antisaccade – gap | Yes  Yes  NA |
| Chehrehnegar et al. (2022) | AD 6M 14F  aMCI 13M 27F  CHOA 23M 36F | 73.52 (7.46)  68.10 (8.81)  62.55 (6.78) | 22.04 (3.27)  25.62 (3.22)  28.16 (1.52) | Antisaccade – overlap | Yes  Yes  NA |
| Opwonya et al. (2022) | MCI 38M 41F  CHOA 72M 98F | 73.3 (7.7)  71.5 (6.2) | ^1^25.8 (3.3)  ^1^27.5 (1.8) | Antisaccade | Yes  NA |
| Opwonya et al. (2022) | MCI 38M 41F  CHOA 72M 98F | 73.3 (7.7)  71.5 (6.2) | ^1^25.8 (3.3)  ^1^27.5 (1.8) | Go/No-Go Antisaccade | Yes  NA |

^1^Korean MMSE, ac – active, ALS – amyotrophic lateral sclerosis, bv – behavioral variant, CBD – cortical basal degeneration, FTD – frontotemporal dementia, g – good, HA – high anxiety, int – performed the Interference task first, LA – low anxiety, MCI/SVD – cerebral small vessel disease originated, MCI/AD – AD originated, md – multi-domain, p – Progressors, pr – poor, rev – performed the reverse task first, rMCI – reverted back to CH, sd – single-domain, se – sedentary, SD – semantic dementia, SE – Standard Error, sMCI – stayed as MCI, VaD – Vascular dementia.

**Table S3. Assessing Shifting ability in Cognitive Aging, and MCI and AD sufferers**

| **Study** | **Participants** | **Age group** | **MMSE (Mean/SD)** | **Task/Test** | **Deficit** |
| --- | --- | --- | --- | --- | --- |
| ***Cognitive Aging studies*** | | | | | |
| Hartman et al. (2001) – Experiment 1 | CHOA 31M 45F  CHYA 31M 54F | 70.3 (4.3)  19.7 (1.7) | > 24  NA | WCST | Yes  NA |
| Hartman et al. (2001) – Experiment 2 | CHOA 22M 26F  CHYA 19M 29F | 69.8 (4.2)  20.3 (2.6) | 29.2 (0.9)  NA | Modified WCST | Yes  NA |
| Souchay & Isingrini (2004) | CHOA M F  CHYA M F | 75.63 (10.22)  23.80 (2.50) | 28.65 (1.43)  NA | WCST | Yes  NA |
| Rhodes & Kelley (2005) | CHOA 50  CHYA 50 | 71.84 (5.40) 19.64 (1.19 | > 27  > 27 | TMT | Yes  NA |
| Rhodes & Kelley (2005) | CHOA 50  CHYA 50 | 71.84 (5.40) 19.64 (1.19 | > 27  > 27 | WCST | Yes  NA |
| Bherer et al. (2006) | CHOA 7M 5F  CHYA 5M 7F | 70.0 (7.0)  20.0 (1.4) | MM 56 (UNK)  NA | TMT part B | Yes  NA |
| Chee et al. (2006) – Experiment 1 | CHOA 6M 11F  CHYA 7M 13F | 66.9 (4.25)  21.3 (1.11) | 28.7 (1.05)  29.4 (0.92) | TMT part B | Yes  NA |
| Hillman et al. (2006) | acCHOA 17  seCHOA 15  acCHYA 18  seCHYA 16 | 63.7 (0.9)  65.9 (0.8)  19.4 (0.3)  19.4 (0.2) | 27.8 (0.4)  29.1 (0.3)  28.9 (0.3)  29.2 (0.3) | Task Switching  paradigm (digit) – global switch cost | Yes  Yes  NA  NA |
| Hillman et al. (2006) | acCHOA 17  seCHOA 15  acCHYA 18  seCHYA 16 | 63.7 (0.9)  65.9 (0.8)  19.4 (0.3)  19.4 (0.2) | 27.8 (0.4)  29.1 (0.3)  28.9 (0.3)  29.2 (0.3) | Task Switching  paradigm (digit) – local switch cost | Yes  Yes  NA  NA |
| Keightley et al. (2006) | CHOA 30  CHYA 30 | 72.5 (7.8)  25.7 (5.1) | 28.8 (0.9)  29.7 (0.5) | TMT | No  NA |
| Damoiseaux et al. (2008) | CHOA 9M 13F  CHYA 5M 5F | 70.73 (6.0)  22.80 (2.3) | 28.73 (1.4)  29.50 (0.5) | TMT part B | No  NA |
| Skinner & Fernandes (2008) | CHOA 30  CHYA 30 | 72.60 (7.05)  20.20 (2.19) | 28.73 (1.26)  NA | TMT part B | Yes  NA |
| Clarys et al. (2009) | CHOA 44  CHYA 44 | 70.75 (6.54)  24.07 (3.45) | > 27  NA | Number-Letter | Yes  NA |
| Clarys et al. (2009) | CHOA 44  CHYA 44 | 70.75 (6.54)  24.07 (3.45) | > 27  NA | WCST | Yes  NA |
| Gamboz et al. (2009) | CHOA 40  CHYA 40 | 67.8 (5.0)  29.2 (4.1) | 29.5 (0.8)  NA | Number-Letter | Yes  NA |
| Gamboz et al. (2009) | CHOA 40  CHYA 40 | 67.8 (5.0)  29.2 (4.1) | 29.5 (0.8)  NA | WCST | Yes  NA |
| Taconnat et al. (2009) | CHOA 15M 47F  CHYA 36M 26F | 69.29 (5.16)  27.58 (5.60) | > 27  NA | WCST | Yes  NA |
| Gold et al. (2010) | CHOA 10 M 10F CHYA 10 M 10F | 68.3 (4.6)  24.3 (3.9) | > 28  NA | Number-Letter | Yes  NA |
| Maquestiaux et al. (2010) | CHOA 3M 9F  CHYA 10M 10F | 63.3 (3.0)  24.6 (2.5) | 29.2 (1.0)  NA | TMT part B | No  NA |
| Albinet et al. (2012) | CHOA 17M 22F  CHYA 11M 17F | 71.2 (4.4)  22.7 (3.3) | 28.4 (1.4)  NA | Dimension-Switching | Yes  NA |
| Albinet et al. (2012) | CHOA 17M 22F  CHYA 11M 17F | 71.2 (4.4)  22.7 (3.3) | 28.4 (1.4)  NA | S-R compatibility switching task | Yes  NA |
| Albinet et al. (2012) | CHOA 17M 22F  CHYA 11M 17F | 71.2 (4.4)  22.7 (3.3) | 28.4 (1.4)  NA | WCST | Yes  NA |
| Boucard et al. (2012) | acCHOA 7M 8F  seCHOA 7M 8F  acCHYOA 7M 8F seCHOYA 7M 8F acCHYA 15M 17F  seCHYA 15M 16F | 73.4 (2.4)  75.4 (3.4)  66.3 (3.0)  66.3 (3.3)  21.9 (1.9)  22.0 (2.7) | 29.2 (0.8)  28.9 (1.0)  29.1 (0.8)  29.1 (1.0)  NA  NA | Dimension-Switching | Yes  Yes  Yes  Yes  NA  NA |
| Boucard et al. (2012) | acCHOA 7M 8F  seCHOA 7M 8F  acCHYOA 7M 8F seCHOYA 7M 8F acCHYA 15M 17F  seCHYA 15M 16F | 73.4 (2.4)  75.4 (3.4)  66.3 (3.0)  66.3 (3.3)  21.9 (1.9)  22.0 (2.7) | 29.2 (0.8)  28.9 (1.0)  29.1 (0.8)  29.1 (1.0)  NA  NA | (Digit) Number–Letter | Yes  Yes  Yes  Yes  NA  NA |
| Boucard et al. (2012) | acCHOA 7M 8F  seCHOA 7M 8F  acCHYOA 7M 8F seCHYOA 7M 8F acCHYA 15M 17F  seCHYA 15M 16F | 73.4 (2.4)  75.4 (3.4)  66.3 (3.0)  66.3 (3.3)  21.9 (1.9)  22.0 (2.7) | 29.2 (0.8)  28.9 (1.0)  29.1 (0.8)  29.1 (1.0)  NA  NA | Plus–Minus | No  No  No  No  NA  NA |
| Laguë-Beauvais et al. (2013) | CHOA 3M 16F  CHYA 8M 13F | 63.47 (3.67) 23.94 (2.32) | 29.00 (1.15)  NA | TMT part B | Yes  NA |
| Wang & Su (2013) | CHOA 16M 16F  CHOM 21M 21F  CHYA 16M 16F | 78.75 (3.07)  69.21 (2.50)  26.53 (3.90) | > 27  > 27  > 27 | WCST | Yes  Yes  NA |
| Müller et al. (2014) | CHOA 8M 12F  CHYA 8M 12F | 70.95 (3.55)  25.70 (3.02) | 29.25 (0.97)  NA | TMT part B | Yes  NA |
| Oosterman et al. (2014) | CHOA 13M 12F  CHYA 15M 11F | 66.1 (11.8)  24.0 (4.0) | UNK  UNK | MCST | Yes  NA |
| Oosterman et al. (2014) | CHOA 13M 12F  CHYA 15M 11F | 66.1 (11.8)  24.0 (4.0) | UNK  UNK | TMT ratio | No  NA |
| Tournier et al. (2014) | CHOA 31  CHYA 30 | 69.61 (7.35)  20.68 (1.89) | 28.93 (1.06)  NA | TMT | Yes  NA |
| Laguë-Beauvais et al. (2015) | CHOA 6M 13F  CHYA 7M 9F | 63.47 (3.67) 23.94 (2.32) | 28.26 (0.93)  NA | TMT part B | Yes  NA |
| Sylvain-Roy et al. (2015) | CHOA 28M 46F  CHYA 33M 42F | 70.9 (6.1)  23.7 (3.9) | 29.0 (1.1)  NA | Left–right shifting | Yes  NA |
| Sylvain-Roy et al. (2015) | CHOA 28M 46F  CHYA 33M 42F | 70.9 (6.1)  23.7 (3.9) | 29.0 (1.1)  NA | Number–Letter | Yes  NA |
| Sylvain-Roy et al. (2015) | CHOA 28M 46F  CHYA 33M 42F | 70.9 (6.1)  23.7 (3.9) | 29.0 (1.1)  NA | Plus–Minus | No  NA |
| Rey-Mermet et al. (2018) | CHOA 124  CHYA 108 | 69.4 (2.9)  22.5 (2.6) | > 26  > 26 | TMT ratio | No  NA |
| Waring et al. (2019) | CHOA 17M 19F  CHYA 24M 20F | 70.42 (7.32)  19.00 (1.14) | 29.17 (1.06)  NA | TMT | Yes  NA |
| Glisky et al. (2020) | CHOA 58M 66F  CHOA 48M 72F  CHYA 24M 20F | 79.6 (4.8)  68.0 (3.5)  21.5 (3.2) | 29 (range = 25-30)  29 (range = 25-30)  NA | Number-letter | Yes  Yes  NA |
| Glisky et al. (2020) | CHOA 58M 66F  CHOA 48M 72F  CHYA 24M 20F | 79.6 (4.8)  68.0 (3.5)  21.5 (3.2) | 29 (range = 25-30)  29 (range = 25-30)  NA | Global-local | No  No  NA |
| Yordanova et al. (2021) | CHOA 43M 75F  CHYA 17M 19F | 70.4 (4.2)  25.2 (2.7) | Assessed, not reported  UNK | TMT | Yes  NA |
| Belghali et al. (2022) | CHOA 15M 11F  CHMA 14M 13F  CHYA 12M 13F  CHAD 12M 13F | 67.00 (4.55)  50.25 (5.15)  26.40 (4.30)  17.40 (1.29) | ≥ 27  ≥ 27  ≥ 27  ≥ 27 | TMT | Yes  No  NA  NA |
| Belghali et al. (2022) | CHOA 15M 11F  CHMA 14M 13F  CHYA 12M 13F  CHAD 12M 13F | 67.00 (4.55)  50.25 (5.15)  26.40 (4.30)  17.40 (1.29) | ≥ 27  ≥ 27  ≥ 27  ≥ 27 | Stroop switching card test | Yes  No  NA  NA |
| Chu et al. (2022) –  Part 1 | CHOA 12M 8F  CHYA 11M 18F | 74.2 (4.2)  25.5 (4.4) | 29.0 (0.8) 29.7 (0.5) | TMT | Yes  NA |
| Chu et al. (2022) – Part 2 | CHOA 10M 11F  CHYA 8M 9F | 72.9 (4.5)  26.8 (4.5) | 29.2 (0.9)  29.8 (0.4) | TMT | Yes  NA |
| ***MCI and AD studies*** | | | | | |
| Perry et al. (2000) | mAD 14  miAD 13  CHOA 30 | 70.1 (9.0)  68.2 (7.6)  67.8 (8.7) | 20.4 (2.0)  26.08 (1.6)  29.4 (0.8) | MCST | Yes  No  NA |
| Perry et al. (2000) | mAD 14  miAD 13  CHOA 30 | 70.1 (9.0)  68.2 (7.6)  67.8 (8.7) | 20.4 (2.0)  26.08 (1.6)  29.4 (0.8) | Visual Elevator | Yes  No  NA |
| Calderon et al. (2001) | AD 6M 3F  CHOA 7M 10F  [DLB 8M 2F] | 71.2 (5.2)  68.3 (5.3)  [72.5 (9.6)] | 21.4 (2.2)  28.8 (1.0)  [20.0 (3.1)] | MCST | Yes  NA  [Yes] |
| Traykov et al. (2002) | AD 6M 3F  CHOA 7M 10F  [VaD 8M 2F] | 82.7 (6.3)  80.4 (5.8)  [81.2 (5.0)] | 23.2 (2.4)  29.2 (0.6)  [23.9 (2.0)] | MCST | Yes  NA  [Yes] |
| Traykov et al. (2002) | AD 6M 3F  CHOA 7M 10F  [VaD 8M 2F] | 82.7 (6.3)  80.4 (5.8)  [81.2 (5.0)] | 23.2 (2.4)  29.2 (0.6)  [23.9 (2.0)] | TMT part B | Yes  NA  [Yes] |
| Kramer et al. (2003) | AD 15M 20F  CHOA  [FTD  SD] | 68.3 (7.5)  67.6 (9.9)  [63.0 (11.9)  67.3 (9.7)] | 24.0 (3.3)  29.5 (0.7)  [24.3 (4.0)  23.4 (3.1)] | Design Fluency | Yes  NA  [Yes  Yes] |
| Kramer et al. (2003) | AD 15M 20F  CHOA  [FTD  SD] | 68.3 (7.5)  67.6 (9.9)  [63.0 (11.9)  67.3 (9.7)] | 24.0 (3.3)  29.5 (0.7)  [24.3 (4.0)  23.4 (3.1)] | Modified TMT | Yes  NA  [Yes  Yes] |
| Nagahama et al. (2003) | AD 54  MCI 17  CHOA 22 | 74.2 (5.1)  72.8 (5.4)  70.8 (9.1) | 20.8 (3.3)  26.4 (2.0)  29.1 (0.8) | MCST | Yes  Yes  NA |
| Nordlund et al. (2005) | MCI 35  CHOA 112 | 64.0 (8.2)  67.0 (5.5) | 28.5 (1.5)  29.3 (1.1) | MCST | No  NA |
| Nordlund et al. (2005) | MCI 35  CHOA 112 | 64.0 (8.2)  67.0 (5.5) | 28.5 (1.5)  29.3 (1.1) | TMT part B | Yes  NA |
| Baudic et al. (2006) | mAD 6M 12F  vmAD 3M 15F  CHOA 3M 14F | 4.1 (0.7)  4.2 (0.9)  5.1 (0.7) | 29.1 (0.6)  25.6 (1.0)  21.2 (1.2) | MCST | Yes  Yes  NA |
| Baudic et al. (2006) | mAD 6M 12F  vmAD 3M 15F  CHOA 3M 14F | .1 (0.7)  4.2 (0.9)  5.1 (0.7) | 29.1 (0.6)  25.6 (1.0)  21.2 (1.2) | TMT part B | Yes  Yes  NA |
| Kramer et al. (2006) | AD 33  aMCI 22  CHOA 35 | 73.4 (9.2)  75.0 (6.1)  73.0 (5.3) | 25.2 (1.3)  28.5 (1.5)  29.5 (0.8) | Modified TMT | Yes  Yes  NA |
| Loewenstein et al.  (2006) | mAD 6M 12F  MCI/AD 3M 15F  MCI/Vas 3M 15F  CHOA 3M 14F | 80.2 (6.0)  78.9 (5.5)  79.7 (7.0)  79.4 (4.0) | 22.9 (2.8)  25.54 (2.1)  27.1 (1.9)  27.7 (1.6) | TMT part B | Yes  Yes  No  NA |
| Lopez et al. (2006) | mixMCI 13M 15F  aMCI 6M 4F  CHOA 142M 232F | 79.7 (5.7)  79.9 (3.4)  79.5 (3.7) | 3MSE 88.2 (7.3)  92.6 (6.2)  96.0 (12.3) | TMT part B | Yes  Yes  NA |
| Stokholm et al. (2006) | AD 16M 20F  CHOA 12M 20F | 76.0 (5.6)  74.3 (4.2) | 25.9 (1.5)  29.3 (0.9) | Design Fluency | Yes  NA |
| Stokholm et al. (2006) | AD 16M 20F  CHOA 12M 20F | 76.0 (5.6)  74.3 (4.2) | 25.9 (1.5)  29.3 (0.9) | TMT part B | Yes  NA |
| Stokholm et al. (2006) | AD 16M 20F  CHOA 12M 20F | 76.0 (5.6)  74.3 (4.2) | 25.9 (1.5)  29.3 (0.9) | Modified WCST | Yes  NA |
| Kramer et al. (2007) | AD 16  CHOA 36  [FTD 30  SD 19] | 60.8 (8.6)  64.4 (10.5)  [58.0 (6.9)  61.9 (6.2)] | 22.8 (4.1)  29.6 (0.6)  [25.6 (3.7)  24.1 (4.6)] | Design Fluency | Yes  NA  [Yes  No] |
| Silveri et al. (2007) | mixMCI 8  naMCI 12  aMCI 13  CHOA 21 | 74.68 (3.77)  66.87 (5.46)  73.85 (6.49)  70.62 (6.27) | 26.00 (1.41)  27.00 (2.67)  26.54 (1.98)  29.05 (0.97) | TMT part B | Yes  No  No  NA |
| Silveri et al. (2007) | mixMCI 8  naMCI 12  aMCI 13  CHOA 21 | 74.68 (3.77)  66.87 (5.46)  73.85 (6.49)  70.62 (6.27) | 26.00 (1.41)  27.00 (2.67)  26.54 (1.98)  29.05 (0.97) | Visual Elevator | Yes  No  Yes  NA |
| Silveri et al. (2007) | mixMCI 8  naMCI 12  aMCI 13  CHOA 21 | 74.68 (3.77)  66.87 (5.46)  73.85 (6.49)  70.62 (6.27) | 26.00 (1.41)  27.00 (2.67)  26.54 (1.98)  29.05 (0.97) | WCST | Yes  No  No  NA |
| Traykov et al. (2007) | MCI 16M 4F  CHOA 14M 6F | 73.2 (8.0)  73.3 (7.0) | 28.95 (1.1)  29.5 (0.5) | MCST | Yes  NA |
| Traykov et al. (2007) | MCI 16M 4F  CHOA 14M 6F | 73.2 (8.0)  73.3 (7.0) | 28.95 (1.1)  29.5 (0.5) | TMT part B | No  NA |
| Zamarian et al. (2007) | AD 6M 9F  CHOA 7M 13F  MCI 11M 7F  CHOA 5M 15F | 77.7 (5.0)  75.3 (4.0)  68.0 (6.9)  66.6 (1.5) | 21.3 (2.2)  29.1 (0.8)  27.0 (1.4)  28.8 (0.8) | TMT part B | NA  NA  Yes  NA |
| Zhang et al. (2007) | MCI 32  CHOA 32 | 73.7 (8.2)  73.5 (8.5) | 27.4 (2.0)  28.7 (1.8) | TMT | Yes  NA |
| Ashendorf et al. (2008) | AD 33M 24F  MCI 82M 118F  CHOA 89M 180F | 79.7 (7.1)  72.5 (8.6)  72.4 (8.5) | 24.2 (3.6)  28.1 (1.9)  29.2 (1.3) | TMT part B | Yes  Yes  NA |
| Belleville et al. (2008) | AD 6M 7F  CHOA 0M 11F  MCI 8M 12F  CHOA 0M 15F | 73.2 (8.1)  72.8 (7.6)  66.3 (10.9)  66.2 (9.6) | 24.85 (4.0)  28.69 (0.8)  28.15 (2.1)  28.9 (0.9) | Task Switching  Paradigm (addition-subtraction condition) – global switch cost | No  NA  No  NA |
| Belleville et al. (2008) | AD 6M 7F  CHOA 0M 11F  MCI 8M 12F  CHOA 0M 15F | 73.2 (8.1)  72.8 (7.6)  66.3 (10.9)  66.2 (9.6) | 24.85 (4.0)  28.69 (0.8)  28.15 (2.1)  28.9 (0.9) | Task Switching  Paradigm (addition-subtraction condition) – local switch cost | No  NA  No  NA |
| Belleville et al. (2008) | AD 6M 7F  CHOA 0M 11F  MCI 8M 12F  CHOA 0M 15F | 73.2 (8.1)  72.8 (7.6)  66.3 (10.9)  66.2 (9.6) | 24.85 (4.0)  28.69 (0.8)  28.15 (2.1)  28.9 (0.9) | Task Switching  Paradigm (left-right condition) – global switch cost | Yes  NA  Yes  NA |
| Belleville et al. (2008) | AD 6M 7F  CHOA 0M 11F  MCI 8M 12F  CHOA 0M 15F | 73.2 (8.1)  72.8 (7.6)  66.3 (10.9)  66.2 (9.6) | 24.85 (4.0)  28.69 (0.8)  28.15 (2.1)  28.9 (0.9) | Task Switching  Paradigm (left-right condition) – local switch cost | Yes  NA  No  NA 16 |
| Ramsden et al. (2008) | AD 4M 11F  CHOA 6M 10F | 70.87 (5.77)  65.75 (4.23) | 20.67 (1.88)  29.06 (1.06) | TMT | Yes  NA |
| Borkowska et al. (2009) | MCI 9M 21F  CHOA 9M 21F  (D 9M 21F) | 61.9 (5.6)  59.7 (7.7)  [55.4 (4.7)] | 25.3 (0.9)  29.5 (1.9)  [29.1 (1.3)] | WCST | Yes  NA  [Yes] |
| Ebert & Anderson (2009) | aMCI 15  CHOA 44  CHYA 27 | 72.3 (5.6)  73.8 (6.1)  22.3 (2.6) | 28.4 (1.8)  29.3 (1.0)  NA | TMT | No  NA  NA |
| Espinosa et al. (2009) | AD 12M 38F  MCI 28M 22F  CHOA 13M 37F | 76.92 (6.35)  74.30 (6.93)  72.26 (7.85) | 21.94 (2.58) 26.06 (2.68)  28.38 (1.68) | Rule Shift Cards | Yes  Yes  NA |
| Lonie et al. (2009) | AD 3M 7F  aMCI 16M 17F  CHOA 8M 13F  [D 3M 14F] | 73.6 (UNK)  73.1 (UNK)  69.5 (UNK)  [73.3 (UNK)] | 25.0 (2.3)  28.4 (1.6)  29.1 (0.7)  [28.6 (1.5)] | TMT part B | Yes  Yes  NA  [Yes] |
| Mandzia et al. (2009) | MCI 7M 7F  CHOA 7M 7F | 68.6 (7.4)  72.2 (6.4) | 27.7 (1.1)  28.6 (1.1) | WCST | No  NA |
| J. L. Price et al. (2009) | AD 38  CHOA 59 | 85.1 (SE 1.6)  83.7 (SE 1.4) | 28.1 (SE 0.4)  28.2 (SE 0.3) | TMT part B | No  NA |
| Schmitter-Edgecombe & Sanders (2009) | MCI 12M 14F  CHOA 12M 14F | 70.88 (9.55)  70.38 (9.10) | 27.38 (1.77)  28.85 (1.22) | Task Switching  paradigm (digit-letter) – global switch cost | Yes  No |
| Schmitter-Edgecombe & Sanders (2009) | MCI 12M 14F  CHOA 12M 14F | 70.88 (9.55)  70.38 (9.10) | 27.38 (1.77)  28.85 (1.22) | TMT part B | Yes  NA |
| Chang et al. (2010) | MCI LEF 137M 58F  MCI HEF 96M 67F  CHOA 115M 107F | 75.52 (7.35)  75.49 (7.30  76.54 (5.04) | 26.98 (1.68)  27.35 (1.75)  29.12 (0.99) | TMT part B | Yes  Yes  NA |
| Hutchison et al. (2010) | mAD 32  CHOA 64 | 78.78 (5.89)  77.24 (9.80) | 28.22 (UNK)  29.19 (UNK) | TMT part B | Yes  NA |
| McGuinness et al. (2010) | AD 28  CHOA 75  [VaD 46] | 77.7 (6.9)  70.2 (7.9)  75.9 (7.8) | ≥ 12  > 28  [≥ 12] | Color Trails | Yes  NA  [Yes] |
| Pa et al. (2010) | AD 6M 4F  MCI 30M 27F  CHOA 20M 20F  [ALS 5M 1F  CBD 4M 8F  FTD 17M 4F  SD 9M 5F] | 62.6 (5.4)  69.8 (9.3)  65.2 (8.9)  [62.0 (10.0)  62.9 (8.5)  60.8 (7.7)  62.1 (5.9)] | 26.0 (3.1)  28.4 (1.5)  29.8 (0.5)  [29.2 (2.0)  27.3 (2.0)  26.1 (4.4)  23.5 (6.2)] | Design Fluency | Yes  Yes  NA  [No  Yes  Yes  Yes] |
| Pa et al. (2010) | AD 6M 4F  MCI 30M 27F  CHOA 20M 20F  [ALS 5M 1F  CBD 4M 8F  FTD 17M 4F  SD 9M 5F] | 62.6 (5.4)  69.8 (9.3)  65.2 (8.9)  [62.0 (10.0)  62.9 (8.5)  60.8 (7.7)  62.1 (5.9)] | 26.0 (3.1)  28.4 (1.5)  29.8 (0.5)  [29.2 (2.0)  27.3 (2.0)  26.1 (4.4)  23.5 (6.2)] | Letter-number TMT | Yes  Yes  NA  [No  Yes  Yes  Yes] |
| S. E. Price et al. (2010) | aMCI 8M 25F  CHOA 9M 24F | 77.61 (7.20)  75.52 (6.17) | 27.4 (1.4)  29.0 (0.9) | TMT | Yes  NA |
| Sinai et al. (2010) | MCI-able 6M 10F  MCI-cue 3M 2F  MCI-unable 2M 4F  CHOA 5M 12F | 75.5 (1.7)  77.0 (2.9)  76.5 (2.6)  75.7 (1.5) | 28.4 (0.4)  26.2 (0.8)  25.17 (0.8)  28.6 (0.4) | Task Switching  paradigm (digit-letter) – global switch cost | No  No  -  NA |
| Sinai et al. (2010) | MCI-able 6M 10F  MCI-cue 3M 2F  MCI-unable 2M 4F  CHOA 5M 12F | 75.5 (1.7)  77.0 (2.9)  76.5 (2.6)  75.7 (1.5) | 28.4 (0.4)  26.2 (0.8)  25.17 (0.8)  28.6 (0.4) | Task Switching  paradigm (digit-letter) – mixing cost | No  Yes  -  NA |
| Sinai et al. (2010) | MCI-able 6M 10F  MCI-cue 3M 2F  MCI-unable 2M 4F  CHOA 5M 12F | 75.5 (1.7)  77.0 (2.9)  76.5 (2.6)  75.7 (1.5) | 28.4 (0.4)  26.2 (0.8)  25.17 (0.8)  28.6 (0.4) | TMT ratio | No  Yes  Yes  NA |
| Tse et al. (2010) | AD 74  CHOA 246  CHYA 32 | 75.82 (7.81)  71.77 (7.71)  20.31 (1.12) | 26.58 (2.78)  28.99 (1.36)  NA | Task Switching  paradigm (digit-letter) – local switch cost | Yes  No  NA |
| Tse et al. (2010) | AD 74  CHOA 246  CHYA 32 | 75.82 (7.81)  71.77 (7.71)  20.31 (1.12) | 26.58 (2.78)  28.99 (1.36)  NA | TMT part B | Yes  NA  NA |
| P. J. Brown et al. (2011) | AD 102M 91F  aMCI 256M 138F  CHOA 119M 110F | 75.33 (7.48)  74.86 (7.40)  75.90 (5.00) | 23.34 (2.06)  27.04 (1.78)  29.11 (1.00) | TMT part B | Yes  Yes  NA |
| Coubard et al. (2011) | AD 3M 14F  CHOA 10M 7F  CHYA 8M 10F | 78.68 (6.15)  77.65 (7.72)  25.36 (2.78) | UNK  UNK  NA | Plus–Minus | Yes  No  NA |
| Coubard et al. (2011) | AD 3M 14F  CHOA 10M 7F  CHYA 8M 10F | 78.68 (6.15)  77.65 (7.72)  25.36 (2.78) | UNK  UNK  NA | Rule shift cards | Yes  Yes  NA |
| Coubard et al. (2011) | AD 3M 14F  CHOA 10M 7F  CHYA 8M 10F | 78.68 (6.15)  77.65 (7.72)  25.36 (2.78) | UNK  UNK  NA | TMT | Yes  No  NA |
| Kessels et al. (2011) | AD 10M 15F  MCI 14M 11F  CHOA 13M 12F | 77.6 (7.2)  77.3 (6.9)  74.4 (6.8) | 21.1 (2.3)  24.9 (2.9)  28.2 (1.5) | TMT part B | Yes  Yes  NA |
| Guerdoux et al. (2012) – Experiment 2 | AD 7M 10F  aMCI 10M 7F  CHOA 11M 6F | 75.0 (8.4)  71.0 (5.9)  72.0 (7.3) | 24.0 (1.9)  27.5 (1.6)  28.4 (1.3) | TMT part B | Yes  No  NA |
| Zheng et al. (2012) | aMCI 14M 20F  CHOA 18M 18F | 67.9 (6.7)  67.4 (5.0) | 28.3 (1.5)  29.5 (0.7) | More-odd shifting | Yes  NA |
| Alichniewicz et al. (2013) | aMCI 5M 18F  CHOA 8M 11F | 60.30 (9.31)  58.84 (7.41) | 28.91 (1.13)  29.32 (0.82) | TMT part B | No  NA |
| Ballesteros et al. (2013) | MCI 10M 10F  CHOA 12M 8F  CHYA 12M 8F | 74.52 (3.94)  69.15 (83.15)  26.25 (1.68) | 24.70 (1.03)  29.40 (0.68)  29.65 (0.49) | WCST | Yes  Yes  NA |
| Bastug et al. (2013) | AD 30  aMCI 30  CHOA 25 | 76.3 (4.54)  72.6 (8.8)  70.0 (6.3) | ^2^24.4 (UNK)  ^2^26 (UNK)  ^2^28 (UNK) | TMT | Yes  Yes  NA |
| Bastug et al. (2013) | AD 30  aMCI 30  CHOA 25 | 76.3 (4.54)  72.6 (8.8)  70.0 (6.3) | ^2^24.4 (UNK)  ^2^26 (UNK)  ^2^28 (UNK) | Oral TMT (OTMT) | Yes  Yes  NA |
| Cangöz et al. (2013) | AD 19M 31F  CHOA 19M 31F | 73.98 (7.35)  74.74 (7.24) | 20.92 (3.03)  27.90 (1.37) | TMT | Yes  NA |
| Chen et al. (2013) | AD 88M 38F  aMCI 82M 38F  CHOA 68M 32F | 78.9 (5.5)  78.2 (7.7)  75.4 (7.3) | 20.2 (3.6)  26.6 (1.4)  28.4 (1.7) | Modified TMT part B | Yes  Yes  NA |
| Chen et al. (2013) | AD 88M 38F  aMCI 82M 38F  CHOA 68M 32F | 78.9 (5.5)  78.2 (7.7)  75.4 (7.3) | 20.2 (3.6)  26.6 (1.4)  28.4 (1.7) | Design Fluency | Yes  Yes  NA |
| Etienne et al. (2013) | AD 10  CHOA 29 | 74.86 (5.36)  70.83 (2.95) | 24.80 (1.48)  29.65 (0.66) | Plus–Minus | Yes  NA |
| Heuer et al. (2013) | AD 16M 12F  MCI 18M 18F  CHOA 49M 69F | 60.9 (1.65)  72.9 (1.12)  69.4 (0.57) | 20.64 (0.92)  28.77 (0.24)  29.54 (0.64) | Design Fluency | Yes  Yes  NA |
| Heuer et al. (2013) | AD 16M 12F  MCI 18M 18F  CHOA 49M 69F | 60.9 (1.65)  72.9 (1.12)  69.4 (0.57) | 20.64 (0.92)  28.77 (0.24)  29.54 (0.64) | Modified TMT | Yes  Yes  NA |
| Lee et al. (2013) | AD 8M 23F  CHOA 5M 26F | 76.45 (5.57) 75.84 (4.74) | ^1^16.16 (5.25)  ^1^25.58 (3.60) | TMT part B | Yes  NA |
| Makizako et al. (2013) | aMCI 21M 15F  CHOA 26M 36F | 76.2 (7.2)  74.0 (6.1) | 27.1 (1.8)  27.0 (2.0) | TMT | No  NA |
| Guild et al. (2014) | sd aMCI 2M 12F  CHOA 22M 26F | 73.07 (6.44)  70.65 (4.47) | 28.14 (1.46)  28.88 (1.36) | TMT part B | No  NA |
| Guild et al. (2014) | sd aMCI 2M 12F  CHOA 22M 26F | 73.07 (6.44)  70.65 (4.47) | 28.14 (1.46)  28.88 (1.36) | MCST | No  NA |
| Peltsch et al. (2014) | AD 22M 50F  aMCI 10M 12F  CHOA 9M 15F | 76.0 (8.0)  76.0 (8.0)  73.0 (6.0) | 27.0 (2.0)  27.0 (2.0)  29.0 (1.0) | WCST | Yes  Yes  NA |
| Peters et al. (2014) | pMCI 8M 10F  sMCI 9M 13F  CHOA 6M 14F | 72.9 (6.3)  70.4 (7.1)  72.0 (6.9) | 27.2 (2.0) 28.1 (1.4)  29.6 (0.5) | TMT part B | No  No  NA |
| Puente et al. (2014) | MCI 7M 10F  CHOA 10M 16F | 75.0 (6.3)  74.0 (5.5) | 25.9 (2.4)  28.0 (2.0) | TMT part B | No  NA |
| Zheng et al. (2014) | aMCI 16M 34F  CHOA 19M 29F | 69.8 (6.8)  69.2 (5.1) | 27.9 (1.5)  29.5 (0.7) | Alternating trail making (TMT part B) | Yes  NA |
| Zheng et al. (2014) | aMCI 16M 34F  CHOA 19M 29F | 69.8 (6.8)  69.2 (5.1) | 27.9 (1.5)  29.5 (0.7) | More-odd shifting | Yes  NA |
| El Haj, Larøi, et al. (2015) | AD 8M 23F  CHOA 10M 23F | 71.42 (5.18)  68.85 (8.21) | 21.68 (1.87)  28.00 (1.52) | Plus–Minus | Yes  NA |
| El Haj, Antoine, & Kapogiannis (2015) | AD 8M 16F  CHOA 9M 17F | 72.08 (7.20)  72.58 (7.20) | 21.83 (1.52)  28.31 (1.28) | Plus–Minus | Yes  NA |
| Huff et al. (2015) | AD 104  CHOA 213  CHMA 208  CHYA 30 | 75.15 (7.74)  75.92 (5.90)  58.08 (7.35)  20.23 (1.01) | 26.62 (3.12)  28.66 (1.41)  29.32 (1.04)  NA | Task Switching  paradigm (digit-letter) – global switch cost | Yes  Yes  Yes  NA |
| Huff et al. (2015) | AD 104  CHOA 213  CHMA 208  CHYA 30 | 75.15 (7.74)  75.92 (5.90)  58.08 (7.35)  20.23 (1.01) | 26.62 (3.12)  28.66 (1.41)  29.32 (1.04)  NA | Task Switching  paradigm (digit-letter) – local switch cost | Yes  No  No  NA |
| Smits et al. (2015) | AD 101M 98F  CHOA 49M 63F  [VaD 6M 4F  DLB 26M 0F  bvFTD 14M 6F  lvFTD 12M 3F] | 65.0 (8.0)  61.0 (8.0)  [67.0 (5.0)  66.0 (9.0)  63.0 (8.0)  63.0 (8.0)] | 22.0 (4.0)  28.0 (1.0)  [25.0 (4.0)  23.0 (3.0)  26.0 (3.0)  24.0 (3.0)] | TMT part B | Yes  NA  [Yes  Yes  No  No] |
| Aurtenetxe et al. (2016) | MCI 11M 9F  CHOA 8M 12F | 73.6 (3.5)  71.7 (2.8) | 28.3 (1.7)  29.4 (0.7) | TMT part B | No  NA |
| Aurtenetxe et al. (2016) | MCI 11M 9F  CHOA 8M 12F | 73.6 (3.5)  71.7 (2.8) | 28.3 (1.7)  29.4 (0.7) | Rule shift cards | Yes  NA |
| Mudar et al. (2016) | aMCI 9M 16F  CHOA 9M 16F | 68.5 (8.0)  65.4 (7.1) | 28.4 (1.3)  28.6 (0.5) | TMT part B | Yes  NA |
| Redondo et al. (2016) | AD 16M 6F  CHOA 11M 12F  [DB 12M 8F] | 77.74 (3.90)  70.92 (4.25)  [70.82 (3.55)] | 23.71 (4.25)  28.12 (1.61)  [26.57 (1.95)] | WCST | Yes  NA  [Yes] |
| Tsai et al. (2016) | aMCI 12M 18F  CHOA 14M 16F | 68.23 (5.25)  66.87 (4.38) | 27.60 (1.99)  28.57 (1.17) | Task Switching  paradigm (digit) – global switch cost | Yes  NA |
| Tsai et al. (2016) | aMCI 12M 18F  CHOA 14M 16F | 68.23 (5.25)  66.87 (4.38) | 27.60 (1.99)  28.57 (1.17) | Task Switching  paradigm (digit) – local switch cost | No  NA |
| Yuan et al. (2016) | aMCI 57M 62F  CHOA 42M 37F | 68.16 (6.67)  69.65 (7.60) | 26.21 (2.69)  28.21 (1.46) | TMT part B | Yes  NA |
| Huang et al. (2017) | AD 11M 20F  CHOA 17M 14F | 78.9 (6.3)  76.5 (5.9) | 21.2 (3.2)  27.0 (1.2) | Color Trails B | Yes  NA |
| Huang et al. (2017) | AD 11M 20F  CHOA 17M 14F | 78.9 (6.3)  76.5 (5.9) | 21.2 (3.2)  27.0 (1.2) | WCST | No  NA |
| Nguyen et al. (2017) | aMCI 8M 14F  CHOA 6M 16F | 68.68 (7.69)  65.32 (6.84) | 28.32 (1.29)  28.75 (0.50) | TMT part B | Yes  NA |
| Holden et al. (2018) | AD 8M 15F  aMCI 11M 18F  CHOA 13M 14F | 70.6 (6.1)  71.3 (7.1)  69.5 (6.1) | 23.3 (2.6) 26.4 (1.8)  28.1 (1.6) | TMT part B | Yes  Yes  NA |
| Matías-Guiu et al. (2018) | AD 7M 12F  CHOA 9M 10F  [bvFTD 9M 10F  ALS 8M 11F] | 72.16 (8.48)  64.89 (8.81)  [71.05 (7.72)  57.89 (9.74)] | 24.26 (4.33)  29.16 (1.21)  [24.00 (4.79)  28.00 (1.63)] | TMT part B | Yes  NA  [Yes  No] |
| Mehrotra & Wagner (2018) | AD 104  CHOA 213  CHMA 246  CHYA 30 | 75.15 (7.74)  75.92 (5.90)  58.08 (7.35)  20.23 (1.01) | 26.62 (3.12)  28.66 (1.41)  29.32 (1.04)  NA | Task Switching  paradigm (digit-letter) – global switch cost | Yes  Yes  Yes  NA |
| Mehrotra & Wagner (2018) | AD 104  CHOA 213  CHMA 246  CHYA 30 | 75.15 (7.74)  75.92 (5.90)  58.08 (7.35)  20.23 (1.01) | 26.62 (3.12)  28.66 (1.41)  29.32 (1.04)  NA | Task Switching  paradigm (digit-letter) – local switch cost | Yes  Yes  Yes  NA |
| Noiret et al. (2018) | AD 9M 11F  CHOA 9M 11F | 79.00 (5.93)  71.75 (3.71) | 21.68 (3,51)  28.80 (1.32) | TMT part B | Yes  NA |
| Garcia-Alvarez et al. (2019) | AD 27M 30F  MCI 27M 21F  CHOA 49M 75F | 76.58 (10.31)  76.68 (10.27)  73.17 (8.60) | 21.21 (4.28)  25.96 (2.03)  28.49 (1.40) | TMT | Yes  Yes  NA |
| Caillaud et al. (2020) | MCI 14M 15F  SCI 25M 42F  CHOA 9M 21F | 76.3 (5.3)  72.3 (5.1)  71.9 (5.7) | 24.3 (1.6) 24.4 (1.9) 25.2 (1.0) | TMT ratio | Yes  No  NA |
| Velichkovsky et al. (2020) | MCI 7M 14F  CHOA 7M 27M  CHYA 19M 38F | 68.5 (10.5)  57.7 (9.8)  24.9 (3.2) | 28.1 (1.2)  29.6 (0.5)  NA | Number-Letter (local switch costs) | Yes  Yes  NA |

^2^ - median scores, CHMA - Cognitive healthy middle-aged adult, DB - Diabetic, DLB - dementia with Lewy bodies, g - good, HEF - higher executive function, LEF - lower executive function, lv - language variant, MCI/Vas - vascular originated, MCST - Modified card sorting test, p - poor, TMT - Trail making test, VaD - vascular dementia, vm - very mild, WCST - Wisconsin card sorting test.

**Table S4. Assessing Updating ability in Cognitive Aging, and MCI and AD sufferers**

| **Study** | **Participants** | **Age group** | **MMSE (Mean/SD)** | **Task/Test** | **Deficit** |
| --- | --- | --- | --- | --- | --- |
| ***Cognitive Aging studies*** | | | | | |
| Clarys et al. (2002) | CHVOA 28  CHOA 27  CHYA 27 | 76.71 (4.04)  65.96 (4.07)  31.85 (6.16) | 28.75 (0.89)  28.63 (0.97)  NA | Alpha span | Yes  Yes  NA |
| Clarys et al. (2002) | CHVOA 28  CHOA 27  CHYA 27 | 76.71 (4.04)  65.96 (4.07)  31.85 (6.16) | 28.75 (0.89)  28.63 (0.97)  NA | Backward word span | Yes  Yes  NA |
| Salat et al. (2002) | CHOA 15M 16F  CHYA 10M 10F | 84.3 (0.9)  29.9 (UNK) | 28.6 (0.2)  NA | N-back (3) | Yes  NA |
| Rhodes & Kelley (2005) | CHOA 50  CHYA 50 | 71.84 (5.40) 19.64 (1.19) | > 27  > 27 | Operation span | Yes  NA |
| Bherer et al. (2006) | CHOA 7M 5F  CHYA 5M 7F | 70.0 (7.0)  20.0 (1.4) | MM 56 (UNK)  NA | BDS | No  NA |
| Chee et al. (2006) – Experiment 1 | CHOA 6M 11F  CHYA 7M 13F | 66.9 (4.25)  21.3 (1.11) | 28.7 (1.05)  29.4 (0.92) | BDS | Yes  NA |
| Chee et al. (2006) – Experiment 1 | CHOA 6M 11F  CHYA 7M 13F | 66.9 (4.25)  21.3 (1.11) | 28.7 (1.05)  29.4 (0.92) | Backward Spatial Span | Yes  NA |
| Keightley et al. (2006) | CHOA 30  CHYA 30 | 72.5 (7.8)  25.7 (5.1) | 28.8 (0.9)  29.7 (0.5) | LNS | Yes  NA |
| Damoiseaux et al. (2008) | CHOA 9M 13F  CHYA 5M 5F | 70.73 (6.0)  22.80 (2.3) | 28.73 (1.4)  29.50 (0.5) | BDS | No  NA |
| McCabe & Hartman (2008) – Experiment 1 | CHOA 36  CHYA 36 | 68.2 (7.1)  19.1 (2.1) | 29.1 (1.0)  NA | N-back (2), verbal | Yes  NA |
| McCabe & Hartman (2008) – Experiment 1 | CHOA 36  CHYA 36 | 68.2 (7.1)  19.1 (2.1) | 29.1 (1.0)  NA | N-back (3), verbal | Yes  NA |
| McCabe & Hartman (2008) – Experiment 2 | CHOA 36  CHYA 36 | 71.5 (7.3)  19.1 (0.8) | 29.2 (1.0)  NA | N-back (2) | Yes  NA |
| McCabe & Hartman (2008) – Experiment 2 | CHOA 36  CHYA 36 | 71.5 (7.3)  19.1 (0.8) | 29.2 (1.0)  NA | N-back (3) | Yes  NA |
| Vaughan et al. (2008) – Experiment 1 | CHOA 58  CHYA 54 | 72.2 (UNK)  18.7 (UNK) | > 27  NA | N-back (4) | Yes  NA |
| Clarys et al. (2009) | CHOA 44  CHYA 44 | 70.75 (6.54)  24.07 (3.45) | > 27  NA | N-back (2) | Yes  NA |
| Daffner et al. (2011) | CHOAh 6M/3F  CHOAl 2M/7F  CHYAh 5M/7F  CHYAl 3M/8F | 72.1 (7.6)  73.6 (4.4)  22.8 (1.9)  22.3 (2.1) | 29.1 (0.9)  29.1 (0.9)  NA  NA | N-back (2) | Yes  Yes  Yes  NA |
| Gamboz et al. (2009) | CHOA 40  CHYA 40 | 67.8 (5.0)  29.2 (4.1) | 29.5 (0.8)  NA | Reading span | Yes  NA |
| Rose et al. (2009) | CHOA 9M 15F  CHYA 7M 17F | 75.1 (6.1)  20.3 (1.6) | 28.2 (1.2)  NA | LNS | Yes  NA |
| Maquestiaux et al. (2010) | CHOA 3M 9F  CHYA 10M 10F | 63.3 (3.0)  24.6 (2.5) | 29.2 (1.0)  UNK | LNS | Yes  NA |
| Morrone et al. (2010) | CHOA 12M 18F  CHYA 10M 20F | 70.0 (3.32)  24.5 (2.81) | 29.5 (0.62)  NA | LNS | No  NA |
| Missonnier et al. (2011) | CHOA 10M 22F  CHYA 13M 19F | 70.2 (7.9)  25.8 (4.6) | UNK  UNK | N-back (3) | Yes  NA |
| Nagel et al. (2011) | CHOA 15M 15F  CHYA 15M 15F | 63.5 (2.7)  24.3 (3.1) | > 26  NA | N-back (3) | Yes  NA |
| Albinet et al. (2012) | CHOA 17M 22F  CHYA 11M 17F | 71.2 (4.4)  22.7 (3.3) | 28.4 (1.4)  NA | Random Number Generation, Redundancy | No  NA |
| Albinet et al. (2012) | CHOA 17M 22F  CHYA 11M 17F | 71.2 (4.4)  22.7 (3.3) | 28.4 (1.4)  NA | Spatial running span task | Yes  NA |
| Albinet et al. (2012) | CHOA 17M 22F  CHYA 11M 17F | 71.2 (4.4)  22.7 (3.3) | 28.4 (1.4)  NA | Verbal running span task | Yes  NA |
| Boucard et al. (2012) | acCHOA 7M 8F  seCHOA 7M 8F  acCHYOA 7M 8F seCHYOA 7M 8F acCHYA 15M 17F  seCHYA 15M 16F | 73.4 (2.4)  75.4 (3.4)  66.3 (3.0)  66.3 (3.3)  21.9 (1.9)  22.0 (2.7) | 29.2 (0.8)  28.9 (1.0)  29.1 (0.8)  29.1 (1.0)  NA  NA | N-back (2) | Yes  Yes  Yes  Yes  NA  NA |
| Boucard et al. (2012) | acCHOA 7M 8F  seCHOA 7M 8F  acCHYOA 7M 8F seCHYOA 7M 8F acCHYA 15M 17F  seCHYA 15M 16F | 73.4 (2.4)  75.4 (3.4)  66.3 (3.0)  66.3 (3.3)  21.9 (1.9)  22.0 (2.7) | 29.2 (0.8)  28.9 (1.0)  29.1 (0.8)  29.1 (1.0)  NA  NA | Spatial Running Span | Yes  Yes  Yes  Yes  NA  NA |
| Boucard et al. (2012) | acCHOA 7M 8F  seCHOA 7M 8F  acCHYOA 7M 8F seCHYOA 7M 8F acCHYA 15M 17F  seCHYA 15M 16F | 73.4 (2.4)  75.4 (3.4)  66.3 (3.0)  66.3 (3.3)  21.9 (1.9)  22.0 (2.7) | 29.2 (0.8)  28.9 (1.0)  29.1 (0.8)  29.1 (1.0)  NA  NA | Verbal Running Span | Yes  Yes  Yes  Yes  NA  NA |
| Laguë-Beauvais et al. (2013) | CHOA 3M 16F  CHYA 8M 13F | 63.47 (3.67) 23.94 (2.32) | 29.00 (1.15)  NA | LNS | No  NA |
| Amer & Hasher (2014) –  Experiment 2 | CHOA 9M 23F  CHYA 11M 21F | 69.81 (5.31)  18.97 (1.91) | 29.19 (1.15)  NA | N-back (1) | Yes  NA |
| Ford et al. (2014) – Experiment 1 | CHOA 10M 22F  CHYA 10M 22F | 73.4 (1.2)  20.1 (0.63) | 29.3 (0.13)  NA | BDS | No  NA |
| Ford et al. (2014) – Experiment 2 | CHOA 8M 24F  CHYA 11M 21F | 70.3 (1.1)  20.8 (0.57) | 29.0 (0.20)  NA | BDS | No  NA |
| Oosterman et al. (2014) | CHOA 13M 12F  CHYA 15M 11F | 66.1 (11.8)  24.0 (4.0) | UNK  UNK | LNS | Yes  NA |
| Pettigrew & Martin (2014) | CHOA 60  CHYA 102 | 71.0 (5.0)  21.0 (3.1) | 28.8 (1.1)  NA | BDS | Yes  NA |
| Pettigrew & Martin (2014) | CHOA 60  CHYA 102 | 71.0 (5.0)  21.0 (3.1) | 28.8 (1.1)  NA | Operation span | Yes  NA |
| Schroeder (2014) | CHOA 18M 24F  CHYA 17M 25F | 73.00 (4.77)  19.45 (1.78) | 28.45 (1.48) 29.05 (1.50) | Alpha span | Yes  NA |
| Schroeder (2014) | CHOA 18M 24F  CHYA 17M 25F | 73.00 (4.77)  19.45 (1.78) | 28.45 (1.48) 29.05 (1.50) | BDS | No  NA |
| Tournier et al. (2014) | CHOA 31  CHYA 30 | 69.61 (7.35)  20.68 (1.89) | 28.93 (1.06)  NA | Operation Span | No  NA |
| Laguë-Beauvais et al. (2015) | CHOA 6M 13F  CHYA 7M 9F | 63.47 (3.67) 23.94 (2.32) | 28.26 (0.93)  NA | LNS | Yes  NA |
| Sylvain-Roy et al. (2015) | CHOA 28M 46F  CHYA 33M 42F | 70.9 (6.1)  23.7 (3.9) | 29.0 (1.1)  NA | Alpha span | Yes  NA |
| Sylvain-Roy et al. (2015) | CHOA 28M 46F  CHYA 33M 42F | 70.9 (6.1)  23.7 (3.9) | 29.0 (1.1)  NA | Keep-track | Yes  NA |
| Sylvain-Roy et al. (2015) | CHOA 28M 46F  CHYA 33M 42F | 70.9 (6.1)  23.7 (3.9) | 29.0 (1.1)  NA | Letter updating | No  NA |
| Sylvain-Roy et al. (2015) | CHOA 28M 46F  CHYA 33M 42F | 70.9 (6.1)  23.7 (3.9) | 29.0 (1.1)  NA | Tone-monitoring | Yes  NA |
| Sylvain-Roy et al. (2015) | CHOA 28M 46F  CHYA 33M 42F | 70.9 (6.1)  23.7 (3.9) | 29.0 (1.1)  NA | Reading span | Yes  NA |
| Kato et al. (2016) | CHOA 10M 10F CHMA 6M 14F  CHYA 8M 12F | 73.9 (2.6)  64.8 (3.0)  22.5 (3.3) | > 27  > 27  NA | N-back (2) | Yes  Yes  NA |
| Berger et al. (2017) | CHOA 7M 18F  CHYA 8M 17F | 68.80 (5.94)  25.32 (4.14) | 29.20 (0.91)  NA | N-back (1) | Yes  NA |
| Berger et al. (2017) | CHOA 7M 18F  CHYA 8M 17F | 68.80 (5.94)  25.32 (4.14) | 29.20 (0.91)  NA | N-back (2) | Yes  NA |
| Glisky et al. (2020) | CHOA 58M 66F  CHOA 48M 72F  CHYA 24M 20F | 79.6 (4.8)  68.0 (3.5)  21.5 (3.2) | 29 (range = 25-30)  29 (range = 25-30)  NA | Consonant updating/letter memory | Yes  Yes  NA |
| Glisky et al. (2020) | CHOA 58M 66F  CHOA 48M 72F  CHYA 24M 20F | 79.6 (4.8)  68.0 (3.5)  21.5 (3.2) | 29 (range = 25-30)  29 (range = 25-30)  NA | Keep track | Yes  Yes  NA |
| Peng et al. (2020) | CHOA 7M 21F  CHMA 3M 25F  CHA 17M 11F  CHYA 2M 26F | 67.68 (4.37)  54.21 (4.06)  31.68 (4.63)  22.71 (1.67) | 27.8 (2.2)  NA  NA  NA | N-back (1) | Yes  Yes  No  NA |
| Yordanova et al. (2021) | CHOA 43M 75F  CHYA 17M 19F | 70.4 (4.2)  25.2 (2.7) | Assessed, not reported  UNK | BDS | Yes  NA |
| Burca et al. (2022) | CHOA 51  CHYA 50 | 74.56 (8.35)  21.48 (4.03) | 29.00 (1.30)  NA | BDS | Yes  NA |
| Chu et al. (2022) –  Part 1 | CHOA 12M 8F  CHYA 11M 18F | 74.2 (4.2)  25.5 (4.4) | 29.0 (0.8) 29.7 (0.5) | BDS | Yes  NA |
| Chu et al. (2022) – Part 2 | CHOA 10M 11F  CHYA 8M 9F | 72.9 (4.5)  26.8 (4.5) | 29.2 (0.9)  29.8 (0.4) | BDS | Yes  NA |
| ***MCI and AD studies*** | | | | | |
| Perry et al. (2000) | mAD 14  miAD 13  CHOA 30 | 70.1 (9.0)  68.2 (7.6)  67.8 (8.7) | 20.4 (2.0)  26.08 (1.6)  29.4 (0.8) | BDS | No  No  NA |
| Calderon et al. (2001) | AD 6M 3F  CHOA 7M 10F  [DLB 8M 2F] | 71.2 (5.2)  68.3 (5.3)  [72.5 (9.6)] | 21.4 (2.2)  28.8 (1.0)  [20.0 (3.1)] | BDS | Yes  NA  [Yes] |
| Belleville et al. (2003) | AD 6M 17F  CHOA 4M 19F  CHOY 7M 8F | 72.8 (5.88)  71.35 (5.59)  24.6 (3.98) | 22.57 (UNK)  UNK  NA | Alphabet span | Yes  No  NA |
| Kramer et al. (2003) | AD 30 - ?M ?F  CHOA  [FTD 11M 10F  SD 8M 6F] | 68.3 (7.5)  67.6 (9.9)  [63.0 (11.9)  67.3 (9.7)] | 24.0 (3.3)  29.5 (0.7)  [24.3 (4.0)  23.4 (3.1)] | BDS | Yes  NA  [Yes  No] |
|  |  |  |  |  |  |
| Grundman et al. (2004) | moAD 83M 100F  mAD 67M 55F  MCI 417M 352F  CHOA 43M 63F | 74.0 (8.0)  73.1 (7.1)  72.9 (7.3)  70.0 (8.3) | 19.9 (3.5)  23.3 (2.4)  27.3 (1.9)  29.1 (1.3) | BDS | UNK  UNK  No  NA |
| Kramer et al. (2006) | AD 33  aMCI 22  CHOA 35 | 73.4 (9.2)  75.0 (6.1)  73.0 (5.3) | 25.2 (1.3)  28.5 (1.5)  29.5 (0.8) | BDS | No  No  NA |
| Levinoff et al. (2006) | AD 40  MCI 73  CHOA 40 | 78.8 (5.9)  74.0 (7.3)  74.1 (7.1) | 25.1 (2.6)  27.7 (1.9)  28.7 (1.2) | BDS | No  No  NA |
| Levinoff et al. (2006) | AD 40  MCI 73  CHOA 40 | 78.8 (5.9)  74.0 (7.3)  74.1 (7.1) | 25.1 (2.6)  27.7 (1.9)  28.7 (1.2) | Backward Spatial Span | Yes  Yes  NA |
| Levinoff et al. (2006) | AD 40  MCI 73  CHOA 40 | 78.8 (5.9)  74.0 (7.3)  74.1 (7.1) | 25.1 (2.6)  27.7 (1.9)  28.7 (1.2) | LNS | Yes  Yes  NA |
| Lopez et al. (2006) | mixMCI 13M 15F  aMCI 6M 4F  CHOA 142M 232F | 79.7 (5.7)  79.9 (3.4)  79.5 (3.7) | 3MSE 88.2 (7.3)  92.6 (6.2)  96.0 (12.3) | BDS | Yes  No  NA |
| Belleville et al. (2007) | AD 19  CHOA 29 in total  MCI 28  CHOA 29 in total | 73.42 (9.18)  72.42 (8.31)  64.76 (10.83)  66.12 (10.09) | 24.65 (3.60)  28.74 (0.93)  28.36 (1.98)  28.88 (0.99) | Alphabet span | Yes  NA  Yes/No  NA |
| Bisiacchi et al. (2008) – Experiment 2 | AD 8M 12F  aMCI 6M 8F  CHOA 5M 9F | 77.65 (6.64)  76.36 (7.41)  78.55 (6.43) | 20.79 (1.92)  25.71 (1.59)  27.80 (1.57) | BDS | Yes  No  NA |
| Bélanger & Belleville (2009) | AD 8  MCI 18  CHOA 16  CHYA 20 | Not reported  Not reported  Not reported  Not reported | 23.5 (4.0)  27.3 (1.8)  29.2 (0.9)  NA | BDS | Yes  Yes  No  NA |
| Borkowska et al. (2009) | MCI 9M 21F  CHOA 9M 21F  [D 9M 21F] | 61.9 (5.6)  59.7 (7.7)  [55.4 (4.7)] | 25.3 (0.9)  29.5 (1.9)  [29.1 (1.3)] | N-back (1) | Yes  NA  [Yes] |
| Mandzia et al. (2009) | MCI 7M 7F  CHOA 7M 7F | 68.6 (7.4)  72.2 (6.4) | 27.7 (1.1)  28.6 (1.1) | BDS | No  NA |
| Schmitter-Edgecombe & Sanders (2009) | MCI 12M 14F  CHOA 12M 14F | 70.88 (9.55)  70.38 (9.10) | 27.38 (1.77)  28.8 (1.22) | LNS | No  NA |
| Zhou & Jia (2009) | MCI/AD 12M 18F  MCI/SVD 36M 20F  CHOA 45M 35F | 72.1 (7.2)  67.3 (6.2)  66.9 (7.1) | 26.2 (1.1)  26.7 (2.2)  28.8 (1.1) | BDS | No  Yes  NA |
| Chang et al. (2010) | MCI LEF 137M 58F  MCI HEF 96M 67F  CHOA 115M 107F | 75.52 (7.35)  75.49 (7.30  76.54 (5.04) | 26.98 (1.68)  27.35 (1.75)  29.12 (0.99) | BDS | Yes  Yes  NA |
| Hutchison et al. (2010) | mAD 32  CHOA 64 | 78.78 (5.89)  77.24 (9.80) | 28.22 (UNK)  29.19 (UNK) | BDS | Yes  NA |
| Muangpaisan et al. (2010) | MCI 12M 14F  CHOA 12M 14F | 66.3 (7.9)  63.7 (7.3) | 26.5 (1.6)  28.1 (1.8) | BDS | Yes  NA |
| Pa et al. (2010) | AD 6M 4F  MCI 30M 27F  CHOA 20M 20F  [ALS 5M 1F  CBD 4M 8F  FTD 17M 4F  SD 9M 5F] | 62.6 (5.4)  69.8 (9.3)  65.2 (8.9)  [62.0 (10.0)  62.9 (8.5)  60.8 (7.7)  62.1 (5.9)] | 26.0 (3.1)  28.4 (1.5)  29.8 (0.5)  [29.2 (2.0)  27.3 (2.0)  26.1 (4.4)  23.5 (6.2)] | BDS | Yes  Yes  NA  [No  Yes  Yes  Yes] |
| Sinai et al. (2010) | MCI-able 6M 10F  MCI-cue 3M 2F  MCI-unable 2M 4F  CHOA 5M 12F | 75.5 (1.7)  77.0 (2.9)  76.5 (2.6)  75.7 (1.5) | 28.4 (0.4)  26.2 (0.8)  25.17 (0.8)  28.6 (0.4) | LNS | No  No  No  NA |
| Tse et al. (2010) | AD 74  CHOA 246  CHYA 32 | 75.82 (7.81)  71.77 (7.71)  20.31 (1.12) | 26.58 (2.78)  28.99 (1.36)  NA | BDS | Yes  NA  NA |
| Ahn et al. (2011) | AD 52M 118F  aMCI 47M 52F  CHOA 56M 86F | 73.5 (7.5)  72.3 (7.2)  66.0 (7.9) | 19.3 (5.0)  26.2 (2.5)  28.7 (1.5) | BDS | Yes  Yes  NA |
| Coubard et al. (2011) | AD 3M 14F  CHOA 10M 7F  CHYA 8M 10F | 78.68 (6.15)  77.65 (7.72)  25.36 (2.78) | UNK  UNK  NA | BDS | Yes  Yes  NA |
| Gagnon & Belleville (2011) | AD 16  aMCI 13 and  md aMCI 7  CHOA 20 | 71.63 (7.27)  73.40 (6.89)  69.90 (7.93) | 23.94 (2.29)  27.95 (1.50)  28.80 (1.06) | Operation Span | Yes  No  NA |
| Kessels et al. (2011) | AD 10M 15F  MCI 14M 11F  CHOA 13M 12F | 77.6 (7.2)  77.3 (6.9)  74.4 (6.8) | 21.1 (2.3)  24.9 (2.9)  28.2 (1.5) | BDS | Yes  Yes  NA |
| Kessels et al. (2011) | AD 10M 15F  MCI 14M 11F  CHOA 13M 12F | 77.6 (7.2)  77.3 (6.9)  74.4 (6.8) | 21.1 (2.3)  24.9 (2.9)  28.2 (1.5) | LNS | Yes  Yes  NA |
| Guerdoux et al. (2012) – Experiment 2 | AD 7M 10F  aMCI 10M 7F  CHOA 11M 6F | 75.0 (8.4)  71.0 (5.9)  72.0 (7.3) | 24.0 (1.9)  27.5 (1.6)  28.4 (1.3) | BDS | Yes  No  NA |
| Johns et al. (2012) | aMCI 18M 22F  CHOA 13M 19F | 72.4 (8.6)  71.8 (5.0) | 28.1 (1.4)  28.9 (1.1) | LNS | Yes  NA |
| Sung et al. (2012) | MCI 16  CHOA 16 | 73.0 (8.0)  70.0 (5.0) | ^1^24.87 (3.40)  ^1^26.45 (2.11) | Alphabet span | Yes  NA |
| Sung et al. (2012) | MCI 16  CHOA 16 | 73.0 (8.0)  70.0 (5.0) | ^1^24.87 (3.40)  ^1^26.45 (2.11) | BDS | Yes  NA |
| Sung et al. (2012) | MCI 16  CHOA 16 | 73.0 (8.0)  70.0 (5.0) | ^1^24.87 (3.40)  ^1^26.45 (2.11) | Backward word span | No  NA |
| Zheng et al. (2012) | aMCI 14M 20F  CHOA 18M 18F | 67.9 (6.7)  67.4 (5.0) | 28.3 (1.5)  29.5 (0.7) | Keep track | Yes  NA |
| Zheng et al. (2012) | aMCI 14M 20F  CHOA 18M 18F | 67.9 (6.7)  67.4 (5.0) | 28.3 (1.5)  29.5 (0.7) | N-back (2) | Yes  NA |
| Bastug et al. (2013) | AD 30  aMCI 30  CHOA 25 | 76.3 (4.54)  72.6 (8.8)  70.0 (6.3) | ^2^24.4 (UNK)  ^2^26 (UNK)  ^2^28 (UNK) | BDS | No  No  NA |
| Doi et al. (2013) | LS aMCI 37  ES aMCI 34  CHOA 29 | 76.8 (7.5)  75.4 (7.2)  72.8 (4.7) | 27.0 (1.9)  26.6 (1.9)  27.6 (2.0) | BDS | Yes  No  NA |
| Chen et al. (2013) | AD 88M 38F  aMCI 82M 38F  CHOA 68M 32F | 78.9 (5.5)  78.2 (7.7)  75.4 (7.3) | 20.2 (3.6)  26.6 (1.4)  28.4 (1.7) | BDS | Yes  No  NA |
| Crawford et al. (2013) | AD 18  CHOA 18  [PD 25] | 78.0 (4.8)  75.0 (3.6)  [63.0 (7.4)] | 20.9 (4.3)  29.2 (1.1)  [28.8 (1.2)] | BDS | Yes  NA  [No] |
| Heuer et al. (2013) | AD 16M 12F  MCI 18M 18F  CHOA 49M 69F | 60.9 (1.65)  72.9 (1.12)  69.4 (0.57) | 20.64 (0.92)  28.77 (0.24)  29.54 (0.64) | BDS | Yes  No  NA |
| Guild et al. (2014) | sd aMCI 2M 12F  CHOA 22M 26F | 73.07 (6.44)  70.65 (4.47) | 28.14 (1.46)  28.88 (1.36) | BDS | No  NA |
| Zheng et al. (2014) | aMCI 16M 34F  CHOA 19M 29F | 69.8 (6.8)  69.2 (5.1) | 27.9 (1.5)  29.5 (0.7) | Keep track | Yes  NA |
| El Haj, Larøi, et al. (2015) | AD 8M 23F  CHOA 10M 23F | 71.42 (5.18)  68.85 (8.21) | 21.68 (1.87)  28.00 (1.52) | N-back (2) | No  NA |
| El Haj, Antoine, & Kapogiannis (2015) | AD 8M 16F  CHOA 9M 17F | 72.08 (7.20)  72.58 (7.20) | 21.83 (1.52)  28.31 (1.28) | N-back (2) | Yes  NA |
| Kessels et al. (2015) | AD 6M 8F  MCI 6M 5F  CHOA 9M 16F | 77.1 (7.7)  78.3 (5.8)  74.8 (7.9) | 19.00 (3.3)  26.7 (1.0)  29.4 (0.8) | BDS | Yes  Yes  NA |
| Kessels et al. (2015) | AD 6M 8F  MCI 6M 5F  CHOA 9M 16F | 77.1 (7.7)  78.3 (5.8)  74.8 (7.9) | 19.00 (3.3)  26.7 (1.0)  29.4 (0.8) | Backward Spatial Span | Yes  No  NA |
| Smits et al. (2015) | AD 101M 98F  CHOA 49M 63F  [VaD 6M 4F  DLB 26M 0F  bvFTD 14M 6F  lvFTD 12M 3F] | 65.0 (8.0)  61.0 (8.0)  [67.0 (5.0)  66.0 (9.0)  63.0 (8.0)  63.0 (8.0)] | 22.0 (4.0)  28.0 (1.0)  [25.0 (4.0)  23.0 (3.0)  26.0 (3.0)  24.0 (3.0)] | BDS | Yes  NA  [Yes  Yes  No  No] |
| Aurtenetxe et al. (2016) | MCI 11M 9F  CHOA 8M 12F | 73.6 (3.5)  71.7 (2.8) | 28.3 (1.7)  29.4 (0.7) | BDS | Yes  NA |
| Mudar et al. (2016) | aMCI 9M 16F  CHOA 9M 16F | 68.5 (8.0)  65.4 (7.1) | 28.4 (1.3)  28.6 (0.5) | BDS | No  NA |
| Pitarque et al. (2016) | AD 7M 23F  aMCI 10M 20F  CHOA 7M 23F  CHYA 14M 28F | 80.03 (1.19)  77.07 (1.19)  75.83 (1.03)  22.14 (0.50) | 20.53 (0.67)  24.83 (0.82)  28.40 (0.28)  NA | BDS | Yes  Yes  NA  NA |
| Redondo et al. (2016) | AD 16M 6F  CHOA 11M 12F  [DB 12M 8F] | 77.74 (3.90)  70.92 (4.25)  [70.82 (3.55)] | 23.71 (4.25)  28.12 (1.61)  [26.57 (1.95)] | N-back (2) | Yes  NA  [No] |
| Redondo et al. (2016) | AD 16M 6F  CHOA 11M 12F  [DB 12M 8F] | 77.74 (3.90)  70.92 (4.25)  [70.82 (3.55)] | 23.71 (4.25)  28.12 (1.61)  [26.57 (1.95)] | N-back (3),  verbal | Yes  NA  [Yes] |
| Liao et al. (2017) | aMCI 28M 33F  CHOA 27M 38F  [D 20M 41F] | 69.7 (6.0)  68.9 (5.8)  [68.2 (5.5)] | 26.3 (2.8)  28.4 (1.4)  [27.8 (1.6)] | BDS | No  NA  [No] |
| Nguyen et al. (2017) | aMCI 8M 14F  CHOA 6M 16F | 68.68 (7.69)  65.32 (6.84) | 28.32 (1.29)  28.75 (0.50) | BDS | No  NA |
| Emrani et al. (2018) | aMCI 15  mixMCI 18  CHOA 33 | 76.20 (6.48)  77.88 (5.46)  77.27 (6.26) | 26.73 (2.21)  26.44 (1.58)  27.69 (1.75) | BDS | Yes  Yes  NA |
| Matías-Guiu et al. (2018) | AD 7M 12F  CHOA 9M 10F  [bvFTD 9M 10F  ALS 8M 11F] | 72.16 (8.48)  64.89 (8.81)  [71.05 (7.72)  57.89 (9.74)] | 24.26 (4.33)  29.16 (1.21)  [24.00 (4.79)  28.00 (1.63)] | BDS | No  NA  [No  No] |
| Cervera-Crespo et al. (2019) | moAD 8M 8F  mAD 7M 8F  CHOA 8M 8F | 79.3 (3.16)  79.1 (6.26)  77.9 (5.14) | 22.46 (1.06)  23.81 (0.91)  28.66 (2.49) | Alpha span | Yes  Yes  NA |
| Garcia-Alvarez et al. (2019) | AD 27M 30F  MCI 27M 21F  CHOA 49M 75F | 76.58 (10.31)  76.68 (10.27)  73.17 (8.60) | 21.21 (4.28)  25.96 (2.03)  28.49 (1.40) | BDS | Yes  Yes  NA |
| Garcia-Alvarez et al. (2019) | AD 27M 30F  MCI 27M 21F  CHOA 49M 75F | 76.58 (10.31)  76.68 (10.27)  73.17 (8.60) | 21.21 (4.28)  25.96 (2.03)  28.49 (1.40) | LNS | Yes  Yes  NA |
| Garcia-Alvarez et al. (2019) | AD 27M 30F  MCI 27M 21F  CHOA 49M 75F | 76.58 (10.31)  76.68 (10.27)  73.17 (8.60) | 21.21 (4.28)  25.96 (2.03)  28.49 (1.40) | N-back (1) | Yes  Yes  NA |
| Ferreira et al. (2019) | moAD 11  mAD 22  CHOA 56  [D 19] | 73.00 (UNK)  75.00 (UNK)  68.50 (UNK)  [67.00 (UNK)] | 19.00 (UNK)  22.50 (UNK)  29.00 (UNK)  [29.00 (UNK)] | BDS | Yes  Yes  NA  [Yes] |

ES - early stage, h - high, LS - late stage, l - low, p - progressive, sev - severe, s - stable.

**S5. Task Descriptions**

| **Task** | **Description** |
| --- | --- |
| ***Dual-Tasking*** | |
| Alphanumeric equation task and a visual detection DT (Compton & Logan, 1991; Logan, 1988) | Participants are required to perform an alphanumeric equation while simultaneously responding to a color change on a computer screen. |
| Auditory discrimination and visual identification task (Bherer et al., 2006) | Participants are required to distinguish between low and high pitch tones whilst identifying which of the two letters (B or C) is presented on a computer screen. |
| Baddeley’s digit recall and tracking task and its variant (Baddeley et al., 1986) | In the recall condition participants must verbally recall a span of digits immediately following their presentation, in the same order heard. Participants completed the span length at which they could recall three different lists correctly for 2 minutes. In the tracking condition, participants tracked the path of a white square on a computer screen at a difficulty based on their performance in a test session by increasing the speed of the moving target. Participants completed this task in three 2 minutes trials. Both tasks also were performed simultaneously for 2 minutes. |
| Digit recall and tracking task (Foley et al., 2013) | In the recall condition, a fixed span length for the participant is used. This is previously established as the maximum span length recalled during a 90 second single-task practice session. In the tracking condition, participants are required to draw a line in successive order through a series of 319 circles arranged along an irregular path across a sheet of A3 paper with a pencil, as fast as they can, from start to finish in 90 seconds. The tasks are performed separately and simultaneously. |
| Della Sala DT (Della Sala et al., 1995) | Pen and paper version of Baddeley’s digit recall and tracking task and its variant (Baddeley et al., 1986). |
| Psychological Refractory paradigm (PRP) (Pashler, 1994; Welford, 1952) | Participants perform two speeded choice-response tasks, e.g., an auditory and visual task or two visual tasks, etc at different stimulus onset asynchronies (SOA, the time between the presentation of the two task). Numerous SOAs may be used in a task, i.e., SOA of 0ms, 100ms, 200ms, etc. |
| Dual- task Stroop paradigm (Ward et al., 2021) | Participants perform the traditional Stroop task as a baseline, followed by two dual-task Stroop variants, a color-dual and a lexical-dual. In the color-dual, participants must complete the traditional Stroop while simultaneously counting the number of times they view a stimulus of a certain color regardless of lexical content. In the lexical-dual, participants must complete the traditional Stroop while simultaneously counting the number of times they view a stimulus of a particular word regardless of the color. |
| The Color and Letter dual-task (Laguë-Beauvais et al., 2015) | This task consisted of blocks of single-trials, single-mixed (SM) trials and dual-mixed (DM) trials. The SP blocks consisted of 20 trials of either the Color or Letter task of 40 trials. In the Color task participants had to determine whether the colour of an X presented above the central point of the computer screen was yellow (A) or green (S) on a QWERTY keyboard with their left middle and index fingers. In the Letter task, participants had to identify the letters B (K) or C (L) presented below the central point of the computer screen by typing on a QWERTY keyboard with their right index and middle fingers.  In the SM trials are single-task trials (only the Color or the Letter task is performed) combined with DM trials, i.e., both single-tasks are present on the screen, one above the another, but the participant is instructed to response to only of the task.  In the DM trials, participants are required to response to both tasks on the screen. In the Priority block condition, participants were instructed to prioritize the Letter task over the Color Task, and in Equal block condition, give equal priority to both tasks. Half the participants completed the Priority condition first and Equal condition second, while the other half completed the Equal condition first and the Priority condition second. |
| Test for Everyday Attention (TEA) dual-task telephone search subtest (Robertson et al., 1994, 2001) | Participants are required to perform a visual scanning task where they searched for a specific telephone code with a matching symbol, and an auditory task, where they counted the number of low frequency tones heard in an audio of combined high and low frequency tones. The tasks are normally performed separately and simultaneously. |
| Visual and auditory processing paradigm (Dannhauser et al., 2005) | Two stimuli, a visual and auditory, are presented in alternating ON and OFF periods. The visual stimulus consisted of a square black and white chequerboard pattern that fills up the entire screen. The squares were reversed at three frequencies (2, 4, 8 Hz) for fixed periods of 16s (ON) alternating with 16s of cross-hair fixation (OFF), and randomized within each set of three consecutive stimulation-fixation cycles. The auditory stimulus consisted of a male voice reading a list of nouns presented at three randomized word rates (30, 60, 90 words/min) for fixed periods of 24s (ON), alternating with 24 s of silence (OFF). |
| Visual stimulus and cognitive DT Makizako et al (2013) | In the visual stimulus, participants are instructed to push a button on the presentation of a bright red light, and in the cognitive test, count backward to 1, where the starting point for counting was selected randomly (from the numbers 100, 90, 80, 70, 60, 50, 40, 30 and 20) by the examiner. |
| DT word span task (Beni et al., 1998) | Participants must verbally read the words presented aloud and to press the animal key whenever they read an animal name. There are three trials for each span size from two to eight. |
| ***Inhibition*** | |
| Antisaccade task (Hallett, 1978; Roberts et al., 1994) | In Sylvain-Roy et al. (2015)’s version a visual cue was presented on either the left or right of the screen followed immediately by a target arrow, on the opposite side. The participants were required to indicate the direction the arrow pointed.  In Crawford et al. (2017)’s version, participants are required to gaze in the opposite direction to a presented red dot. In the first modified version, a memory-guided antisaccade task, participants were presented with a randomly placed red dot as the target, along with four adjacently placed green dots as distractors. Firstly, they were instructed to gaze at the target in the first condition, then secondly, they had to gaze at the location of the previously presented target on a blank screen. In the second version of the task, a go/no-go antisaccade task was used. The presentation of a centrally placed red cross denoted a ‘no-go’ response, while a green cross required a ‘go’ response.  In Chehrehnegar et al. (2022)’s version, two different paradigms are performed, a gap and an overlap. In the gap, where at the start of the task a central fixation cross is presented for 1000 or 1500 ms randomly before turning red and disappears for 200 ms. The target stimuli then appear for 2000 ms. In the overlap, the fixation cross is displayed for 200 ms during the target presentation, and the target is then presented for an additional 2000 ms. |
| Flanker task (Eriksen & Eriken, 1974) | Participants are required to respond to a centrally placed stimulus "flanked" by concurrently presented irrelevant stimuli (that can be congruent or incongruent with the central stimuli), e.g., <<< **<** <<< or <<< **>** <<<. |
| Modified flanker task (Van’t Ent, 2002) | Standard task with two addition conditions, a PRO and ANTI. In the PRO condition, responses correspond to the target arrow, in the ANTI, responses do not correspond to target arrow, i.e., the responses used in the PRO condition were reversed in the ANTI. To distinguish between the conditions, different colors were used for the target arrow. |
| Global-local task (Hübner & Malinowski, 2002) | Participants are presented with element letters forming a large letter (e.g., a large “Y” formed from small “V”s). In the global task, participants must identify the global elements (i.e., “Y”) while suppressing the response induced by the local letters. In the local task, participants must identify the local elements (i.e., the small “V”s) while suppressing the response induced by the global letter. |
| Go/no-go task (Newman & Kosson, 1986) | Participants are required to respond to the appearance of a specific stimulus (‘go’ condition) but withhold responses on the presentation of a different stimulus (‘no-go’ condition). |
| Emotional go/no-go task (Waring et al., 2019) | The go/no-go task with the use of various facial expressions, i.e., happy, sad, as stimuli. |
| Hayling Sentence Completion Test (HSCT) (Burgess & Shallice, 1997) | Participants are required to complete a high cloze sentence with a missing last word. In part A, the initiation section, the congruent condition of the test, a related, expected word should be provided. In part B, the inhibition section, the incongruent condition, an unrelated, unexpected word should be provided. |
| Emotional HSCT (Dupart et al., 2018) | Analog of the HSCT using emotionally charged sentences and comparing the words the participants produced as either emotionally neutral, positive, or negative. |
| Stroop task (Stroop, 1935) | Participants must complete three sections each consisting of 100 items, part 1) a word naming (congruent) where the words **RED**, **GREEN**, and **BLUE** printed in black ink, part 2) an ink color naming (congruent) where XXXX was written in the colors green, blue, and red, i.e., **XXXX**, **XXXX** and **XXXX**, and part 3) the naming of the ink color of the word (incongruent) section, where the words in part 1 are printed in the colors of the items in part 2, i.e., **RED**, **GREEN**, or **BLUE**. Participants perform these tasks as quickly as possible, usually within a specific timeframe, e.g., within 45 seconds per section. |
| Emotional Stroop (Agustí et al., 2017; Kamboureli & Economou, 2021; Meléndez et al., 2020; Satorres et al., 2020) | Participant are presented with images of happy or sad faces with a word ‘happy’ or ‘sad’ superimposed. In the congruent condition the face and word represent the same expression, i.e., happy, and in the incongruent, they are not. Participants complete ‘face’ and ‘word’ blocks where they have to respond with the facial expression or word, respectively. Performance between the congruent and incongruent conditions is compared. |
| Number Stroop (Salthouse & Meinz, 1995) | Participants must count the number of one to four centrally presented digits while ignoring the numerical value of the digit. In congruent trials, the number of digits corresponds to the digits displayed. In incongruent trials, the number of digits does not correspond to the digits displayed. In neutral trials, unrelated symbols were displayed (e.g., $$). |
| Modified Stroop (Bohnen et al., 1992) | Stroop task with an added fourth condition where participants were required to switch between naming the color of the ink and naming of ink color of the word, i.e., the incongruent condition. |
| Delis–Kaplan Executive Function System Color-Word Interference test (D-KES CWIT) (Delis et al., 2001) | Stroop task with an added a fourth condition, where participants instead switch between word naming and naming of the ink color of the word. |
| Math Stroop (Zamarian et al., 2007) | Participants must complete three conditions arithmetic. In the first condition, participants complete pure blocks of either addition or multiplication problems. In the second condition, participants complete a mixed block consisting of both addition and multiplication problems. In the third block, participants must solve addition problems as multiplication and vice versa. |
| Interference and Reverse Stroop (Amieva et al., 2004) | Briefer version of the Stroop, consisting of cards with the names, **BLUE**, **RED**, **YELLOW**, **GREEN** printed in a contrasting ink color. In the Interference version, participants must identify the word, and in the Reverse version, participants must identify the ink color. |
| Victoria Stroop (Spreen & Strauss, 1998) | This is a briefer version of the traditional Stroop task, consisting of three stimulus cards comprised of 24 items, where participants are required to quickly name either the, 1) color of dots (Dot condition-Card, 1), 2) color of the ink of the neutral words printed (Word condition-Card 2), and 3) color of the ink in which the words (names) are printed (Interference condition-Card 3). |
| Nonverbal Stroop task (Pettigrew & Martin, 2014) | Task comprises three conditions, a neutral condition, where participants were presented with a stimulus in the centre of a computer screen, e.g., left-pointing arrow. In the congruent condition, the stimulus is on the same side the arrow is pointing, e.g., left-pointing arrow on the left side of the screen. In incongruent condition, the stimulus is on the opposite size the arrow is pointing, e.g., a left-pointing arrow on the right side of the screen. The participants were required to respond with the direction the arrow was pointing, right or left. |
| Picture-word interference task (Lupker, 1979; Schriefers et al., 1990) | This task involves the completion of two conditions, an interference condition, where a picture is superimposed with a distractor word from the same semantic category, and a non-interference condition, where a picture is superimposed with a distractor word from a different semantic category. Participants were required to respond with what was seen in the picture, while ignoring the word. |
| N-2 repetition paradigm (Mayr & Keele, 2000) | Participants are presented with groups of four different stimuli (i.e., rectangles with different dimension - orientation, fill or size). One stimulus differs in a dimension from the remaining three stimuli. Following a cue. Participants must indicate which of the four stimuli is different. |
| Negative priming (Tipper, 1985) | Participants are required to respond to a stimulus that was previously presented as a distractor in trial (n), so becoming the target. In the studies reviewed the incongruent condition of the Stroop task was used, where what was ignored in the previous trial was attended to in the present trial. |
| Negative compatibility task (Eimer & Schlaghecken, 1998; Schlaghecken et al., 2012) | Participants are first presented with a centrally positioned prime (a left or right arrow or a symbol in neutral trials), then a blank screen followed by a mask period consisting of an overlap of all the exemplars of the prime for a period. Followed by a blank screen for a time period, and then the target arrow. Participants must indicate the direction of the target arrow. In congruent trials, the prime arrow and target arrow are in the same direction. In incongruent trials, the prime arrow and target arrow are in different directions. |
| Positive compatibility task (Eimer & Schlaghecken, 1998; Schlaghecken et al., 2012) | Same as the negative compatibility task but the presentations of the prime, mask and target arrow occur immediately after each other. |
| Random number generation task (Audiffren et al., 2009) | Participants are required to produce a number between 1 and 9 verbally every time a computer-generated tone is heard, approximately every second, such that a string of numbers is generated randomly. 100 responses are recorded, usually within 100 seconds. The total adjacency score (%), i.e., the distribution of adjacent digits (in ascending or descending series) from the ordinal sequence of alternatives (i.e., 1–2; or 8–7–6) is measured. Successful performance requires the efficiency of two EFs, the correct inhibition of overlearned schemas (i.e., counting) and correct updating of working memory (WM). |
| Simon task (Simon, 1969) | Similar to the nonverbal Stroop minus the neutral condition, where in response to the color of the shape of a stimulus, i.e., a red or green circle or square, participants had to respond left or right. |
| Stop-signal task (Logan et al., 1984; B. R. Williams et al., 1999) | Participants must perform a specific task as quickly as they can following the presentation of a ‘go’ signal and stop following a ‘stop’ signal during the duration of a trial. |
| ***Shifting*** | |
| Behavioral Assessment of the Dysexecutive Syndrome rule shift cards task (Wilson et al., 1996) | The task consists of 21 nonpictorial playing cards. In part 1, participants are required to respond with “Yes” to a red card and “No” to a black card. In the second part, a new rule is provided, respond “Yes” if the presented card is the same color as the previous turned card and “No” if the color is different. Therefore, participants have to modify their responses, inhibiting their original response set and shift their thought process. |
| Design fluency test (Harter et al., 1999; Jones-Gotman & Milner, 1977) | Participants are required to complete three test conditions to create different designs in ‘n’ number of squares by using four straight lines to connect. The first condition requires connecting filled unnumbered dots, 2) unfilled dots, and 3) the shifting condition, alternate between connecting filled and unfilled dots, all within 60 seconds for each condition. |
| Dimension-switching task (Albinet et al., 2012; Monsell & Mizon, 2006; Rogers & Monsell, 1995) | Participants are presented with the word, LEFT or RIGHT, enclosed in a left or right arrow, presented above or below the center of a white screen. Depending on the location of the presented stimulus, participants are required to respond with the direction either printed in text (the word) or the direction of the arrow. Participants completed word and arrow only task blocks, as well as blocks of pseudo-randomly mixed word and arrow trial. |
| Trail making test (Reitan, 1992; Reitan & Wolfson, 1986) | This test encompasses two parts, in part A, the participant is required to connect 25 numbered (1, 2, 3, etc) dots or circles, in sequential order, and in part B, alternate between letters and numbers in ascending order (1, A, 2, B, etc). |
| Alternating trail making version (Schmitter-Edgecombe & Sanders, 2009) | Analogous of the traditional trail making test part B. |
| Letter-number TMT (Pa et al., 2010) | Entails completing a letter only, and number only, and switching condition. In the first two conditions, participants must connect the letters or numbers in serial order. In the switching, they must alternate between numbers and letters in sequential order. |
| Modified TMT (Heuer et al., 2013; Kramer et al., 2006) | Entails serially alternating between connecting numbers and days of the week. |
| Modified TMT part B test (Chen et al., 2013) | Entails alternating connecting lines between numbers and weekday circles, within 60 seconds. |
| Oral trail making test (Bastug et al., 2013) | In part A, participants are instructed to verbally count from 1 to 25, and in part B, to alternate between counting numbers and letters as seen in the paper version, 1-A-2-B, etc |
| Color trails test (D’Elia et al., 1996) | Analogous of the trial making test, was utilized by two studies, Huang et al (2017) and McGuinness et al (2010), in AD participants. It requires participants to complete two parts, in part 1, the participants connect circles numbered 1 to 25 in ascending order, and part 2, connect the numbers 1 to 25 in order, but alternate between two colors (i.e., 1-pink-2-blue-3-pink-, etc). |
| Left–right shifting task (Belleville et al., 2008) | Participants are required to identify one of two digits presented on the left side of a screen in the first block, and on the right side of a screen in the second block. In the shifting block, the target number is randomly placed on either side of the screen and indicated by a visual cue. |
| More-odd shifting task (Salthouse et al., 1998; Zheng et al., 2012) | Participants complete three task conditions, 1) respond either “greater” or “less” if a red number presented on a screen is larger or smaller then a five, respectively, 2) respond with “odd” or “even” when the number is colored green, and 3) a combination of the conditions 1) and 2) in one block, where the participant is cued to the task to perform. |
| Number-letter task (Rogers & Monsell, 1995) | Participants are required to classify whether a number-letter pair presented in one of four boxes in the center of a computer screen are either odd or even, or a vowel or consonant. More precisely, whether the number is odd or even when the pair is seen in one of the top two boxes, during the number task, or if the letter is a vowel or consonant when the pair is seen in one of the bottom two boxes, during the letter task. |
| Plus-minus task (Jersild, 1927; Miyake et al., 2000; Spector & Biederman, 1976) | Participants are required to complete three conditions, 1) to add a specific number to every number presented, 2) subtracts a specific number from every number presented, and 3) alternate between adding and subtracting a specific number. |
| S-R compatibility switching task (Albinet et al., 2012; Monsell & Mizon, 2006; Rogers & Monsell, 1995) | Participants are presented with a screen containing a white frame. At the start of each trial, the white frame changes color to red or green. After a duration of 250ms or 1750ms, a left or right pointing arrow is presented at a random location within the frame. Participants are required to respond by pressing a button located either on the side indicated by the arrow, when the frame is green, or the opposite side, when the red frame. Participants complete single blocks of one frame color, and then mixed blocks with both frame color occurring pseudo-randomly. |
| Stroop switching card test (Belghali et al., 2022) | Participants are presented with four different cards (red, blue, green, and yellow), placed in front of them and are given 36 cards with text (red, blue, green, and yellow) in the four colors on either black or gray backgrounds. Participants must say aloud quickly the ink color of the text if the background on the card is gray, or the written text when the background is black. Then place the cards onto one of the four colored cards placed in front of them corresponding to the color named. |
| Task switching paradigm (Rogers & Monsell, 1995) | Participants are required to perform two conditions, a repetition condition, where participants complete the same task repeatedly in a block (two different tasks are completed, i.e., an audio and a visual task), and a shifting condition, where the completion of the two repetition tasks presented pseudo randomly within the same block is required. |
| Visual elevator (Robertson et al., 2001) | Part of the TEA, participants are required to count upwards or downwards as they follow a series of visually presented numbers corresponding to floors in an elevator. The task demands the participants shift the direction of counting. |
| Wisconsin card sorting task (Berg, 1948; Nelson, 1976) | Participants are presented with a number of stimulus cards with sets of symbols that vary in color, shape, and number (e.g., 3 green triangles or 2 yellow squares). They are instructed to categorize them according to a particular dimension (i.e., color, shape, or number). The category rule changes every time 10 (out of a maximum of 128) response cards have been sorted correctly, but the participants are unaware of this pattern. |
| ***Updating*** | |
| (Alpha)bet span task (Belleville et al., 1998; Craik et al., 2018) | Participants must either repeat a list of words in the same serial order presented to them or mentally rearrange them into alphabetical order. |
| Backward digit recall span test (WAIS-R or WAIS-III) (Egeland, 2015; Griffin & Heffernan, 1983; Wechsler, 2012) | Participants must immediately recall a list of digits previously presented in reverse order. The span length ranges from two to eight, and each length is completed twice. |
| Backward spatial span (Wechsler, 1987) | Participants are required to recall various sequence spans presented on a screen in reverse order. |
| Keep track task (Yntema, 1963) | Participants are required to keep track of 15 words presented in sequential order and remember the last (most recent) word from one of ‘n’ categories presented, e.g., colors or animals. They must respond with the last word at the end of the trial. In Sylvain-Roy et al. (2015)’s version, participants read a series of semantically correct or anomalous sentences, and judge each, i.e., with ‘yes’ or ‘no’, for semantic plausibility, in addition to remembering the last word of the sentence. They must recall all the last words verbally at the end of each series, which varies from two to five sentences of four blocks per series length. |
| Letter-number sequencing (Egeland, 2015; Wechsler, 2012) | Participants must recall a sequence of previously presented randomly mixed letters and numbers in sequential order, i.e., letters alphabetically ordered first (A, B, C, etc), and then numbers in ascending numerical order (1, 2, 3, etc). |
| Letter updating task (Sylvain-Roy et al., 2015) | Participants orally recall the last consonant seen in a series of visually presented consonants. The number of consonants to be recalled is determined individually for the participant prior to the start of the actual task, minus one item, during a practice session. The participants are presented with a series of four different lengths, 1) the participant’s span minus one, 2) the participant’s span plus one, 3) the participant’s span plus three, and 4) the participant’s span plus five items, randomly. |
| N-back task (Jaeggi et al., 2010; Kirchner, 1958) | Participants respond with the position of a stimulus presented on a screen ‘n’ position(s) prior. In the non-spatial version, participants are required to recognize a stimulus presented at ‘n’ screen positions prior. In the spatial version, the position on the screen the stimulus is presented is required. For example, at 0-back trial, the position of the stimulus at 0 position (the present) screen is required. During 1-back, the position a screen prior to the present screen is required, and so on. The higher the n-back position, the greater the WM demand. Thus, a 3-back task will consist of the completion of the 0-, 1-, 2- and 3-back conditions. |
| Operation span (Turner & Engle, 1989) | A mathematical operation and an item (word or letter) are presented to the participant. They must verbally say if the operation is correct (i.e., true or false) and remember the item presented. At the end of each trial, the participant must recall the items in serial order. After three consecutive errors the task is terminated. Typically, the task consists of 15 trials with two to six operation-word pairs, 3 trials per length. |
| Random number generation task (Baddeley, 1998) | Previously described under inhibition. It also assesses updating ability by measuring the Redundancy score (%), which is based on the rate on which individual digits are utilized. A score of 0% suggests no redundancy, i.e., good randomness, and no repetitiveness of digits (e.g., 5, 1, 7, 9, 3, 6, 2, 4, 8), whilst a score of 100% equates to complete redundancy, i.e., repeated use of the same response choice, throughout (e.g., 1, 1, 1, 1, 1, 1, 1, 1, 1, 1). |
| Reading span (Daneman & A.Carpenter, 1980) | Typically involves participants verbally read sentences and remember the last word of each sentence in a set or block. They must recall all the last words verbally in the order of each set. The sentences get increasingly longer in a set until the participant fails three in a row. |
| Spatial running span task (Albinet et al., 2012; Boucard et al., 2012; Morris & Jones, 1990) | Participants are presented with an empty 4 x 4 matrix on a computer screen, where sequences of six, eight, ten, or twelve black dots are presented randomly in one of the 16 squares of the matrix, at a rate of a dot every two seconds. No location is repeated in the sequence, and the sequence length is never known. Participants are required to recall the last four dot locations at the end of each sequence in strict forward serial recall order using a computer mouse. They must complete twelve sequences, three for each length. |
| Tone-monitoring task (Larson et al., 1988; Miyake et al., 2000) | Participants are required to keep track of the number of a series of low, medium, and high pitch tones presented randomly. The participants are instructed to press an appropriate keyboard button when they heard three tones of the same pitch. |
| Verbal running span task (Albinet et al., 2012; Boucard et al., 2012; Morris & Jones, 1990) | Participants are presented with a list of six, eight, ten, and twelve consonants on a computer screen, every two seconds. Participants are instructed to recall the last four consonants at the end of each sequence, strict forward serial recall. The sequence length is never known. They must complete twelve sequences, three for each length. |
| Word backward span (Yeom et al., 1992) | Essentially the same as the backward digit span but with words instead of digits. Participants are read various increasing span lengths of words and required to immediately verbally recall the span of words in reverse order. |

# References

Agustí, A. I., Satorres, E., Pitarque, A., & Meléndez, J. C. (2017). An emotional Stroop task with faces and words. A comparison of young and older adults. *Consciousness and Cognition*, *53*(March), 99–104. https://doi.org/10.1016/j.concog.2017.06.010

Ahn, H.-J., Seo, S. W., Chin, J., Suh, M. K., Lee, B. H., Kim, S. T., Im, K., Lee, J.-M., Lee, J. H., Heilman, K. M., & Na, D. L. (2011). The cortical neuroanatomy of neuropsychological deficits in mild cognitive impairment and Alzheimer’s disease: A surface-based morphometric analysis. *Neuropsychologia*, *49*(14), 3931–3945. https://doi.org/10.1016/j.neuropsychologia.2011.10.010

Aisenberg, D., Cohen, N., Pick, H., Tressman, I., Rappaport, M., Shenberg, T., & Henik, A. (2015). Social priming improves cognitive control in elderly adults - Evidence from the Simon task. *PLoS ONE*, *10*(1), 1–17. https://doi.org/10.1371/journal.pone.0117151

Aisenberg, D., Sapir, A., D’Avossa, G., & Henik, A. (2014). Long trial durations normalise the interference effect and sequential updating during healthy aging. *Acta Psychologica*, *153*, 169–178. https://doi.org/10.1016/j.actpsy.2014.10.005

Albinet, C. T., Boucard, G., Bouquet, C. A., & Audiffren, M. (2012). Processing speed and executive functions in cognitive aging: How to disentangle their mutual relationship? *Brain and Cognition*, *79*(1), 1–11. https://doi.org/10.1016/j.bandc.2012.02.001

Alichniewicz, K. K., Brunner, F., Klünemann, H. H., & Greenlee, M. W. (2013). Neural correlates of saccadic inhibition in healthy elderly and patients with amnestic mild cognitive impairment. *Frontiers in Psychology*, *4*(JUL), 1–12. https://doi.org/10.3389/fpsyg.2013.00467

Amer, T., & Hasher, L. (2014). Conceptual Processing of Distractors by Older but Not Younger Adults. *Psychological Science*, *25*(12), 2252–2258. https://doi.org/10.1177/0956797614555725

Amieva, H., Lafont, S., Rouch-Leroyer, I., Rainville, C., Dartigues, J. F., Orgogozo, J. M., & Fabrigoule, C. (2004). Evidencing inhibitory deficits in Alzheimer’s disease through interference effects and shifting disabilities in the Stroop test. *Archives of Clinical Neuropsychology*, *19*(6), 791–803. https://doi.org/10.1016/j.acn.2003.09.006

Andrés, P., Guerrini, C., Phillips, L. H., & Perfect, T. J. (2008). Differential effects of aging on executive and automatic inhibition. *Developmental Neuropsychology*, *33*(2), 101–123. https://doi.org/10.1080/87565640701884212

Ashendorf, L., Jefferson, A. L., O’Connor, M. K., Chaisson, C., Green, R. C., & Stern, R. A. (2008). Trail Making Test errors in normal aging, mild cognitive impairment, and dementia. *Archives of Clinical Neuropsychology*, *23*(2), 129–137. https://doi.org/10.1016/j.acn.2007.11.005

Audiffren, M., Tomporowski, P. D., & Zagrodnik, J. (2009). Acute aerobic exercise and information processing: Modulation of executive control in a Random Number Generation task. *Acta Psychologica*, *132*(1), 85–95. https://doi.org/10.1016/j.actpsy.2009.06.008

Aurtenetxe, S., García-Pacios, J., Río, D. del, López, M. E., Pineda-Pardo, J. A., Marcos, A., Losada, M. L. D., López-Frutos, J. M., & Maestú, F. (2016). Interference impacts working memory in mild cognitive impairment. *Frontiers in Neuroscience*, *10*(OCT). https://doi.org/10.3389/fnins.2016.00443

Baddeley, A. (1998). Random generation and the executive control of working memory. *The Quarterly Journal of Experimental Psychology: Section A*, *51*(4), 819–852.

Baddeley, A., Baddeley, H. A., Bucks, R. S., & Wilcock, G. K. (2001). Attentional control in Alzheimer’s disease. *Brain*, *124*(8), 1492–1508. https://doi.org/10.1093/brain/124.8.1492

Baddeley, A., Logie, R. H., Bressi, S., Della Sala, S., & Spinnler, H. (1986). Dementia and Working Memory. *The Quarterly Journal of Experimental Psychology Section A*, *38*(4), 603–618. https://doi.org/10.1080/14640748608401616

Ballesteros, S., Mayas, J., & Reales, J. M. (2013). Cognitive function in normal aging and in older adults with mild cognitive impairment. *Psicothema*, *25*(1), 18–24. https://doi.org/10.7334/psicothema2012.181

Bastug, G., Ozel-Kizil, E. T., Sakarya, A., Altintas, O., Kirici, S., & Altunoz, U. (2013). Oral trail making task as a discriminative tool for different levels of cognitive impairment and normal aging. *Archives of Clinical Neuropsychology*, *28*(5), 411–417. https://doi.org/10.1093/arclin/act035

Baudic, S., Barba, G. D., Thibaudet, M. C., Smagghe, A., Remy, P., & Traykov, L. (2006). Executive function deficits in early Alzheimer’s disease and their relations with episodic memory. *Archives of Clinical Neuropsychology*, *21*(1), 15–21. https://doi.org/10.1016/j.acn.2005.07.002

Bélanger, S., & Belleville, S. (2009). Semantic Inhibition Impairment in Mild Cognitive Impairment: A Distinctive Feature of Upcoming Cognitive Decline? *Neuropsychology*, *23*(5), 592–606. https://doi.org/10.1037/a0016152

Bélanger, S., Belleville, S., & Gauthier, S. (2010). Inhibition impairments in Alzheimer’s disease, mild cognitive impairment and healthy aging: Effect of congruency proportion in a Stroop task. *Neuropsychologia*, *48*(2), 581–590. https://doi.org/10.1016/j.neuropsychologia.2009.10.021

Belghali, M., Statsenko, Y., & Laver, V. (2022). Stroop switching card test: brief screening of executive functions across the lifespan. *Aging, Neuropsychology, and Cognition*, *29*(1), 14–33. https://doi.org/10.1080/13825585.2020.1844865

Belleville, S., Bherer, L., Lepage, É., Chertkow, H., & Gauthier, S. (2008). Task switching capacities in persons with Alzheimer’s disease and mild cognitive impairment. *Neuropsychologia*, *46*(8), 2225–2233. https://doi.org/10.1016/j.neuropsychologia.2008.02.012

Belleville, S., Chertkow, H., Davis, M. B., Hospital, J. G., & Gauthier, S. (2007). Working Memory and Control of Attention in Persons With Alzheimer’s Disease and Mild Cognitive Impairment. *Neuropsychology*, *21*(4), 458–469. https://doi.org/10.1037/0894-4105.21.4.458

Belleville, S., Rouleau, N., & Caza, N. (1998). Effect of normal aging on the manipulation of information in working memory. *Memory & Cognition*, *26*(3), 572–583. https://doi.org/10.3758/BF03201163

Belleville, S., Rouleau, N., Linden, M. Van Der, Collette, F., Neuropsychologie, S. De, & Belleville, S. (2003). Effect of Manipulation and Irrelevant noise on Working Memory Capacity of Patients with Alzheimer’s Dementia. *Neuropsychology*, *17*, 69–81.

Belleville, S., Rouleau, N., & Van der Linden, M. (2006). Use of the Hayling task to measure inhibition of prepotent responses in normal aging and Alzheimer’s disease. *Brain and Cognition*, *62*(2), 113–119. https://doi.org/10.1016/j.bandc.2006.04.006

Beni, R. De, Palladino, P., Pazzaglia, F., & Cornoldi, C. (1998). Increases in intrusion errors and working memory deficit of poor comprehenders. *The Quarterly Journal of Experimental Psychology Section A Human Experimental Psychology*, *51*(2), 305–320.

Berg, E. A. (1948). A simple objective technique for measuring flexibility in thinking. *The Journal of General Psychology*, *39*(1), 15–22.

Berger, N., Richards, A., & Davelaar, E. J. (2017). When emotions matter: Focusing on emotion improves working memory updating in older adults. *Frontiers in Psychology*, *8*(SEP), 1–13. https://doi.org/10.3389/fpsyg.2017.01565

Bherer, L., Kramer, A. F., Peterson, M. S., Colcombe, S., Erickson, K., & Becic, E. (2006). Testing the limits of cognitive plasticity in older adults : Application to attentional control. *Acta Psychologica*, *123*, 261–278. https://doi.org/10.1016/j.actpsy.2006.01.005

Bisiacchi, P. S., Borella, E., Bergamaschi, S., Carretti, B., & Mondini, S. (2008). Interplay between memory and executive functions in normal and pathological aging. *Journal of Clinical and Experimental Neuropsychology*, *30*(6), 723–733. https://doi.org/10.1080/13803390701689587

Bohnen, N., Jolles, J., & Twijnstra, A. (1992). Modification of the Stroop Color Word Test improves differentiation between patients with mild head injury and matched controls. *Clinical Neuropsychologist*, *6*(2), 178–184. https://doi.org/10.1080/13854049208401854

Borella, E., Carretti, B., Mitolo, M., Zavagnin, M., Caffarra, P., Mammarella, N., Fairfield, B., Gamboz, N., & Piras, F. (2017). Characterizing cognitive inhibitory deficits in mild cognitive impairment. *Psychiatry Research*, *251*, 342–348. https://doi.org/10.1016/j.psychres.2016.12.037

Borkowska, A., Drozdz, W., Jurkowski, P., & Rybakowski, J. K. (2009). The Wisconsin Card Sorting Test and the N-back test in mild cognitive impairment and elderly depression. *The World Journal of Biological Psychiatry*, *10*(4), 870–876. https://doi.org/10.3109/15622970701557985

Borsa, V. M., Della Rosa, P. A., Catricalà, E., Canini, M., Iadanza, A., Falini, A., Abutalebi, J., & Iannaccone, S. (2018). Interference and conflict monitoring in individuals with amnestic mild cognitive impairment: A structural study of the anterior cingulate cortex. *Journal of Neuropsychology*, *12*(1), 23–40. https://doi.org/10.1111/jnp.12105

Boucard, G. K., Albinet, C. T., Bugaiska, A., Bouquet, C. A., Clarys, D., & Audiffren, M. (2012). Impact of physical activity on executive functions in aging: A selective effect on inhibition among old adults. *Journal of Sport and Exercise Psychology*, *34*(6), 808–827. https://doi.org/10.1123/jsep.34.6.808

Brambati, S. M., Belleville, S., & Kergoat, M.-J. (2009). Single and Multiple Domain Amnestic MCI: two sides of the same coin? *Dementia and Geriatric Cognitive Disorders*, *28*(6), 541–549. https://doi.org/10.1159/000255240

Brown, P. J., Devanand, D. P., Liu, X., & Caccappolo, E. (2011). Functional impairment in elderly patients with mild cognitive impairment and mild Alzheimer disease. *Archives of General Psychiatry*, *68*(6), 617–626. https://doi.org/10.1001/archgenpsychiatry.2011.57

Burca, M., Chausse, P., Ferrand, L., Parris, B. A., & Augustinova, M. (2022). Some further clarifications on age-related differences in the Stroop task: New evidence from the two-to-one Stroop paradigm. *Psychonomic Bulletin and Review*, *29*(2), 492–500. https://doi.org/10.3758/s13423-021-02011-x

Burgess, P. W., & Shallice, T. (1997). The Hayling and Brixton Tests. In *Bury St Edmunds: Thames Valley Test Company.*

Caillaud, M., Hudon, C., Boller, B., Brambati, S., Duchesne, S., Lorrain, D., Gagnon, J. F., Maltezos, S., Mellah, S., Phillips, N., & Belleville, S. (2020). Evidence of a relation between hippocampal volume, white matter hyperintensities, and cognition in subjective cognitive decline and mild cognitive impairment. *Journals of Gerontology - Series B Psychological Sciences and Social Sciences*, *75*(7), 1382–1392. https://doi.org/10.1093/geronb/gbz120

Calderon, J., Perry, R. J., Erzinclioglu, S. W., Berrios, G. E., Dening, T. R., & Hodges, J. R. (2001). Perception, attention, and working memory are disproportionately impaired in dementia with Lewy bodies compared with Alzheimer’s disease. *Journal of Neurology Neurosurgery and Psychiatry*, *70*(2), 157–164. https://doi.org/10.1136/jnnp.70.2.157

Cangöz, B., Demirci, S., & Uluç, S. (2013). Trail making test: Predictive validity study on Turkish patients with Alzheimer dementia. *Turk Geriatri Dergisi*, *16*(1), 69–76. https://www.scopus.com/inward/record.uri?eid=2-s2.0-84875635212&partnerID=40&md5=41406d3bf89e21a7e31000848c21bb82

Cervera-Crespo, T., González-Alvarez, J., & Rosell-Clarí, V. (2019). Semantic inhibition and dementia severity in Alzheimer’s disease. *Psicothema*, *31*(3), 305–310. https://doi.org/10.7334/psicothema2019.40

Chang, Y. L., Jacobson, M. W., Fennema-Notestine, C., Hagler, D. J., Jennings, R. G., Dale, A. M., & McEvoy, L. K. (2010). Level of executive function influences verbal memory in amnestic mild cognitive impairment and predicts prefrontal and posterior cingulate thickness. *Cerebral Cortex*, *20*(6), 1305–1313. https://doi.org/10.1093/cercor/bhp192

Chee, M. W. L., Goh, J. O. S., Venkatraman, V., Jiat, C. T., Gutchess, A., Sutton, B., Hebrank, A., Leshikar, E., & Park, D. (2006). Age-related changes in object processing and contextual binding revealed using fMR adaptation. *Journal of Cognitive Neuroscience*, *18*(4), 495–507. https://doi.org/10.1162/jocn.2006.18.4.495

Chehrehnegar, N., Shati, M., Esmaeili, M., & Foroughan, M. (2022). Executive function deficits in mild cognitive impairment: evidence from saccade tasks. *Aging and Mental Health*, *26*(5), 1001–1009. https://doi.org/10.1080/13607863.2021.1913471

Chen, N.-C., Chang, C.-C., Lin, K.-N., Huang, C.-W., Chang, W.-N., Chang, Y.-T., Chen, C., Yeh, Y.-C., & Wang, P.-N. (2013). Patterns of executive dysfunction in amnestic mild cognitive impairment. *International Psychogeriatrics*, *25*(07), 1181–1189. https://doi.org/10.1017/S1041610213000392

Chu, N. C. W., Sturnieks, D. L., Lord, S. R., & Menant, J. C. (2022). Visuospatial working memory and obstacle crossing in young and older people. *Experimental Brain Research*, *240*(11), 2871–2883. https://doi.org/10.1007/s00221-022-06458-9

Cid-Fernández, S., Lindín, M., & Díaz, F. (2014). Effects of amnestic mild cognitive impairment on N2 and P3 Go/NoGo ERP Components. *Journal of Alzheimer’s Disease*, *38*(2), 295–306. https://doi.org/10.3233/JAD-130677

Clarys, D., Bugaiska, A., Tapia, G., & Baudouin, A. (2009). Ageing, remembering, and executive function. *Memory*, *17*(2), 158–168. https://doi.org/10.1080/09658210802188301

Clarys, D., Isingrini, M., & Gana, K. (2002). Mediators of age-related differences in recollective experience in recognition memory. *Acta Psychologica*, *109*(3), 315–329. https://doi.org/10.1016/S0001-6918(01)00064-6

Clément, F., Gauthier, S., & Belleville, S. (2013). Executive functions in mild cognitive impairment: Emergence and breakdown of neural plasticity. *Cortex*, *49*(5), 1268–1279. https://doi.org/10.1016/j.cortex.2012.06.004

Colcombe, S. J., Kramer, A. F., Erickson, K. I., & Scalf, P. (2005). The implications of cortical recruitment and brain morphology for individual differences in inhibitory function in aging humans. *Psychology and Aging*, *20*(3), 363–375. https://doi.org/10.1037/0882-7974.20.3.363

Collette, F., Van der Linden, M., Delrue, G., & Salmon, E. (2002). Frontal hypometabolism does not explain inhibitory dysfunction in Alzheimer disease. *Alzheimer Disease and Associated Disorders*, *16*(4), 228–238. https://doi.org/10.1097/00002093-200210000-00004

Compton, B. J., & Logan, G. D. (1991). The transition from algorithm to retrieval in memory-based theories of automaticity. *Memory & Cognition*, *19*(2), 151–158. https://doi.org/10.3758/BF03197111

Coubard, O. A., Ferrufino, L., Boura, M., Gripon, A., Renaud, M., & Bherer, L. (2011). Attentional Control in Normal Aging and Alzheimer’s Disease. *Neuropsychology*, *25*(3), 353–367. https://doi.org/10.1037/a0022058

Coxon, J. P., Goble, D. J., Leunissen, I., Van Impe, A., Wenderoth, N., & Swinnen, S. P. (2016). Functional Brain Activation Associated with Inhibitory Control Deficits in Older Adults. *Cerebral Cortex*, *26*(1), 12–22. https://doi.org/10.1093/cercor/bhu165

Craik, F. I. M., Bialystok, E., Gillingham, S., & Stuss, D. T. (2018). Alpha span: A measure of working memory. *Canadian Journal of Experimental Psychology/Revue Canadienne de Psychologie Expérimentale*, *72*(3), 141–152.

Crawford, T. J., Higham, S., Mayes, J., Dale, M., Shaunak, S., & Lekwuwa, G. (2013). The role of working memory and attentional disengagement on inhibitory control: Effects of aging and Alzheimer’s disease. *Age*, *35*(5), 1637–1650. https://doi.org/10.1007/s11357-012-9466-y

Crawford, T. J., Higham, S., Renvoize, T., Patel, J., Dale, M., Suriya, A., & Tetley, S. (2005). Inhibitory control of saccadic eye movements and cognitive impairment in Alzheimer’s disease. *Biological Psychiatry*, *57*(9), 1052–1060. https://doi.org/10.1016/j.biopsych.2005.01.017

Crawford, T. J., Smith, E. S., & Berry, D. M. (2017). Eye gaze and aging: Selective and combined effects of working memory and inhibitory control. *Frontiers in Human Neuroscience*, *11*(November), 1–10. https://doi.org/10.3389/fnhum.2017.00563

D’Elia, L. F., Satz, P., Uchiyama, C. L., & White, T. (1996). *Color Trails Test*. Professional manual, Psychological Assessment Resources.

Daffner, K. R., Chong, H., Sun, X., Tarbi, E. C., Riis, J. L., Mcginnis, S. M., & Holcomb, P. J. (2011). Mechanisms underlying age- and performance-related differences in working memory. *Journal of Cognitive Neuroscience*, *23*(6), 1298–1314. https://doi.org/10.1162/jocn.2010.21540

Damoiseaux, J. S., Beckmann, C. F., Arigita, E. J. S., Barkhof, F., Scheltens, P., Stam, C. J., Smith, S. M., & Rombouts, S. A. R. B. (2008). Reduced resting-state brain activity in the “default network” in normal aging. *Cerebral Cortex*, *18*(8), 1856–1864. https://doi.org/10.1093/cercor/bhm207

Daneman, M., & A.Carpenter, P. (1980). Individual differences in working memory and aging. *Journal of Verbal Learning and Verbal Behaviour*, *19*, 450–466. https://doi.org/10.4324/9781315879840-7

Dannhauser, T. M., Walker, Z., Stevens, T., Lee, L., Seal, M., & Shergill, S. S. (2005). The functional anatomy of divided attention in amnestic mild cognitive impairment. *Brain*, *128*(6), 1418–1427. https://doi.org/10.1093/brain/awh413

Delis, D. C., Kaplan, E., & Kramer, J. H. (2001). *Delis-Kaplan executive function system*.

Della Sala, S., Baddeley, A., Papagno, C., & Spinnler, H. (1995). Dual-Task Paradigm: A Means To Examine the Central Executive. *Annals of the New York Academy of Sciences*, *769*(1), 161–172. https://doi.org/10.1111/j.1749-6632.1995.tb38137.x

Della Sala, S., Cocchini, G., Logie, R. H., Allerhand, M., & MacPherson, S. E. (2010). Dual task during encoding, maintenance, and retrieval in Alzheimer’s disease. *Journal of Alzheimer’s Disease*, *19*(2), 503–515. https://doi.org/10.3233/JAD-2010-1244

Doi, T., Shimada, H., Makizako, H., Yoshida, D., Shimokata, H., Ito, K., Washimi, Y., Endo, H., & Suzuki, T. (2013). Characteristics of cognitive function in early and late stages of amnestic mild cognitive impairment. *Geriatrics & Gerontology International*, *13*(1), 83–89. https://doi.org/10.1111/j.1447-0594.2012.00865.x

Duong, A., Whitehead, V., Hanratty, K., & Chertkow, H. (2006). The nature of lexico-semantic processing deficits in mild cognitive impairment. *Neuropsychologia*, *44*(10), 1928–1935. https://doi.org/10.1016/j.neuropsychologia.2006.01.034

Dupart, M., Auzou, N., & Mathey, S. (2018). Emotional valence impacts lexical activation and inhibition differently in aging: an emotional Hayling task investigation. *Experimental Aging Research*, *44*(3), 206–220. https://doi.org/10.1080/0361073X.2018.1449587

Dwolatzky, T., Whitehead, V., Doniger, G. M., Simon, E. S., Schweiger, A., Jaffe, D., & Chertkow, H. (2003). Validity of a novel computerized cognitive battery for mild cognitive impairment. *BMC Geriatrics*, *12*, 1–12.

Ebert, P. L., & Anderson, N. D. (2009). Proactive and retroactive interference in young adults, healthy older adults, and older adults with amnestic mild cognitive impairment. *Journal of the International Neuropsychological Society*, *15*(1), 83–93. https://doi.org/10.1017/S1355617708090115

Egeland, J. (2015). Measuring working memory with digit span and the letter-number sequencing subtests from the WAIS-IV: Too low manipulation load and risk for underestimating modality effects. *Applied Neuropsychology:Adult*, *22*(6), 445–451. https://doi.org/10.1080/23279095.2014.992069

Eimer, M., & Schlaghecken, F. (1998). Effects of Masked Stimuli on Motor Activation: Behavioral and Electrophysiological Evidence. *Journal of Experimental Psychology: Human Perception and Performance*, *24*(6), 1737–1747. https://doi.org/10.1037/0096-1523.24.6.1737

El Haj, M., Antoine, P., & Kapogiannis, D. (2015). Flexibility Decline Contributes to Similarity of Past and Future Thinking in Alzheimer’s Disease. *Hippocampus*, *25*(11), 1447–1455. https://doi.org/10.1002/hipo.22465.Flexibility

El Haj, M., Larøi, F., Gély-Nargeot, M. C., & Raffard, S. (2015). Inhibitory deterioration may contribute to hallucinations in Alzheimer’s disease. *Cognitive Neuropsychiatry*, *20*(4), 281–295. https://doi.org/10.1080/13546805.2015.1023392

Emrani, S., Libon, D. J., Lamar, M., Price, C. C., Jefferson, A. L., Gifford, K. A., Hohman, T. J., Nation, D. A., Delano-Wood, L., Jak, A., Bangen, K. J., Bondi, M. W., Brickman, A. M., Manly, J., Swenson, R., & Au, R. (2018). Assessing Working Memory in Mild Cognitive Impairment with Serial Order Recall. *Journal of Alzheimer’s Disease*, *61*(3), 917–928. https://doi.org/10.3233/JAD-170555

Endrass, T., Schreiber, M., & Kathmann, N. (2012). Speeding up older adults: Age-effects on error processing in speed and accuracy conditions. *Biological Psychology*, *89*(2), 426–432. https://doi.org/10.1016/j.biopsycho.2011.12.005

Eriksen, B. A., & Eriken, C. W. (1974). Effects of noise letters upon selective identification of letters. In *Perceptions & Psychophysics* (Vol. 16, Issue 1, pp. 143–149). https://doi.org/10.4992/jjpsy.56.125

Espinosa, A., Boada, M., Vinyes, G., Valero, S., Martínez-lage, P., Peña-casanova, J., James, T., Wilson, B. A., & Tárraga, L. (2009). Ecological Assessment of executive functions in mild cognitive impairment and mild Alzheimer’s Disease. *Journal of the International Neuropsychological Society*, *15*(5), 751–757. https://doi.org/10.1017/S135561770999035X.Ecological

Etienne, V., Marin-Lamellet, C., & Laurent, B. (2013). Mental flexibility impairment in drivers with early Alzheimer’s disease: A simulator-based study. *IATSS Research*, *37*(1), 16–20. https://doi.org/10.1016/j.iatssr.2013.06.005

Ferreira, J. V., de Araujo, N. B., de Oliveira, F., Plácido, J., Sant’ Anna, P., Monteiro-Junior, Sobral, R., Marinho, V., Deslandes, A., & Laks, J. (2019). Dual task in healthy elderly, depressive and Alzheimer’s disease patients. *Jornal Brasileiro de Psiquiatria*, *68*(4), 200–207. https://doi.org/10.1590/0047-2085000000247

Foley, J. A., Kaschel, R., & Della Sala, S. (2013). Dual tasking in Alzheimer’s disease and depression. *Zeitschrift Fur Neuropsychologie*, *24*(1), 25–33. https://doi.org/10.1024/1016-264X/a000089

Foley, J. A., Kaschel, R., Logie, R. H., & Della Sala, S. (2011). Dual-task performance in Alzheimers disease, mild cognitive impairment, and normal ageing. *Archives of Clinical Neuropsychology*, *26*(4), 340–348. https://doi.org/10.1093/arclin/acr032

Ford, J. H., Rubin, D. C., & Giovanello, K. S. (2014). Effects of task instruction on autobiographical memory specificity in young and older adults. *Memory*, *22*(6), 722–736. https://doi.org/10.1080/09658211.2013.820325

Gagnon, L. G., & Belleville, S. (2011). Working Memory in Mild Cognitive Impairment and Alzheimer’s Disease: Contribution of Forgetting and Predictive Value of Complex Span Tasks. *Neuropsychology*, *25*(2), 226–236. https://doi.org/10.1037/a0020919

Gamboz, N., Borella, E., & Brandimonte, M. A. (2009). The role of switching, inhibition and working memory in older adults’ performance in the Wisconsin Card Sorting Test. *Aging, Neuropsychology, and Cognition*, *16*(3), 260–284. https://doi.org/10.1080/13825580802573045

Garcia-Alvarez, L., Gomar, J. J., Sousa, A., Garcia-Portilla, M. P., & Goldberg, T. E. (2019). Breadth and depth of working memory and executive function compromises in mild cognitive impairment and their relationships to frontal lobe morphometry and functional competence. *Alzheimer’s and Dementia: Diagnosis, Assessment and Disease Monitoring*, *11*, 170–179. https://doi.org/10.1016/j.dadm.2018.12.010

Gold, B. T., Powell, D. K., Xuan, L., Jicha, G. A., & Smith, C. D. (2010). Age-related slowing of task switching is associated with decreased integrity of frontoparietal white matter. *Neurobiology of Aging*, *31*(3), 512–522. https://doi.org/10.1016/j.neurobiolaging.2008.04.005

Griffin, P. T., & Heffernan, A. (1983). Digit span, forward and backward: Separate and unequal components of the WAIS digit span. *Perception and Motor Skillsceptual and Motor Skills*, *56*(1), 335–338. https://doi.org/10.2466%2Fpms.1983.56.1.335

Grundman, M., Petersen, R. C., Ferris, S. H., Thomas, R. G., Aisen, P. S., Bennett, D. A., Foster, N. L., Jack, C. R., Galasko, D. R., Doody, R., Kaye, J., Sano, M., Mohs, R., Gauthier, S., Kim, H. T., Jin, S., Schultz, A. N., Schafer, K., Mulnard, R., … Thal, L. J. (2004). Mild Cognitive Impairment Can Be Distinguished from Alzheimer Disease and Normal Aging for Clinical Trials. *Archives of Neurology*, *61*(1), 59–66. https://doi.org/10.1001/archneur.61.1.59

Guerdoux, E., Dressaire, D., Martin, S., Adam, S., & Brouillet, D. (2012). Habit and recollection in healthy aging, mild cognitive impairment, and alzheimer’s disease. *Neuropsychology*, *26*(4), 517–533. https://doi.org/10.1037/a0028718

Guild, E. B., Vasquez, B. P., Maione, A. M., Mah, L., Ween, J., & Anderson, N. D. (2014). Dynamic working memory performance in individuals with single-domain amnestic mild cognitive impairment. *Journal of Clinical and Experimental Neuropsychology*, *36*(7), 751–760. https://doi.org/10.1080/13803395.2014.941790

Hallett, P. E. (1978). Primary and secondary saccades to goals defined by instructions. *Vision Research*, *18*(10), 1279–1296.

Harter, S. L., Hart, C. C., & Harter, G. W. (1999). Expanded scoring criteria for the design fluency test: Reliability and validity in neuropsychological and college samples. *Archives of Clinical Neuropsychology*, *14*(5), 419–432. https://doi.org/10.1016/S0887-6177(98)00033-X

Hartman, M., Bolton, E., & Fehnel, S. E. (2001). Accounting for age differences on the Wisconsin Card Sorting Test: Decreased working memory, not inflexibility. *Psychology and Aging*, *16*(3), 385–399. https://doi.org/10.1037/0882-7974.16.3.385

Heuer, H. W., Mirsky, J. B., Kong, E. L., Dickerson, B. C., Miller, B. L., Kramer, J. H., & Boxer, A. L. (2013). Antisaccade task reflects cortical involvement in mild cognitive impairment. *Neurology*, *81*(14), 1235–1243. https://doi.org/10.1212/WNL.0b013e3182a6cbfe

Hillman, C. H., Kramer, A. F., Belopolsky, A. V, & Smith, D. P. (2006). A cross-sectional examination of age and physical activity on performance and event-related brain potentials in a task switching paradigm. *International Journal of Psychophysiology*, *59*(1), 30–39. https://doi.org/10.1016/j.ijpsycho.2005.04.009

Holden, J. G., Cosnard, A., Laurens, B., Asselineau, J., Biotti, D., Cubizolle, S., Dupouy, S., Formaglio, M., Koric, L., Seassau, M., Tilikete, C., Vighetto, A., & Tison, F. (2018). Prodromal Alzheimer’s Disease Demonstrates Increased Errors at a Simple and Automated Anti-Saccade Task. *Journal of Alzheimer’s Disease*, *65*(4), 1209–1223. https://doi.org/10.3233/JAD-180082

Hsieh, S., Liang, Y. C., & Tsai, Y. C. (2012). Do age-related changes contribute to the flanker effect? *Clinical Neurophysiology*, *123*(5), 960–972. https://doi.org/10.1016/j.clinph.2011.09.013

Hsieh, S., Wu, M., & Tang, C. H. (2016). Adaptive strategies for the elderly in inhibiting irrelevant and conflict no-go trials while performing the Go/No-Go task. *Frontiers in Aging Neuroscience*, *7*(JAN), 1–14. https://doi.org/10.3389/fnagi.2015.00243

Huang, S. F., Liu, C. K., Chang, C.-C., & Su, C. Y. (2017). Sensitivity and specificity of executive function tests for Alzheimer’s disease. *Applied Neuropsychology:Adult*, *24*(6), 493–504. https://doi.org/10.1080/23279095.2016.1204301

Hübner, R., & Malinowski, P. (2002). The effect of response competition on functional hemispheric asymmetries for global/local processing. *Perception and Psychophysics*, *64*(8), 1290–1300. https://doi.org/10.3758/BF03194772

Huff, M. J., Balota, D. A., Minear, M., Aschenbrenner, A. J., & Duchek, J. M. (2015). Dissociative global and local task-switching costs across younger adults, middle-aged adults, older adults, and very mild Alzheimer’s disease individuals. *Psychology and Aging*, *30*(4), 727–739. https://doi.org/10.1037/pag0000057

Hutchison, K. A., Balota, D. A., & Ducheck, J. M. (2010). The utility of stroop task switching as a marker for early-stage alzheimer’s disease. *Psychology and Aging*, *25*(3), 545–559. https://doi.org/10.1037/a0018498

Jaeggi, S. M., Buschkuehl, M., Perrig, W. J., & Meier, B. (2010). The concurrent validity of the N-back task as a working memory measure. *Memory*, *18*(4), 394–412. https://doi.org/10.1080/09658211003702171

Jennings, J. M., Dagenbach, D., Engle, C. M., & Funke, L. J. (2007). Age-related changes and the attention network task: An examination of alerting, orienting, and executive function. *Aging, Neuropsychology, and Cognition*, *14*(4), 353–369. https://doi.org/10.1080/13825580600788837

Jersild, A. T. (1927). Mental set and shift. *Archives of Psychology*, *89*, 5–82.

Johns, E. K., Phillips, N. A., Belleville, S., Goupil, D., Babins, L., Kelner, N., Ska, B., Gilbert, B., Massoud, F., De Boysson, C., Duncan, H. D., & Chertkow, H. (2012). The profile of executive functioning in amnestic mild cognitive impairment: Disproportionate deficits in inhibitory control. *Journal of the International Neuropsychological Society*, *18*, 541–555. https://doi.org/10.1017/S1355617712000069

Jones-Gotman, M., & Milner, B. (1977). Design fluency: The invention of nonsense drawings after focal cortical lesions. *Neuropsychologia*, *15*(4–5), 653–674. https://doi.org/10.1016/0028-3932(77)90070-7

Kamboureli, C., & Economou, A. (2021). Trait anxiety and interference in the emotional Stroop task in young and old adults. *Current Psychology*. https://doi.org/10.1007/s12144-021-02199-0

Kaschel, R., Logie, R. H., Kazén, M., & Della Sala, S. (2009). Alzheimer’s disease, but not ageing or depression, affects dual-tasking. *Journal of Neurology*, *256*(11), 1860–1868. https://doi.org/10.1007/s00415-009-5210-7

Kato, K., Nakamura, A., Kato, T., Kuratsubo, I., Yamagishi, M., Iwata, K., & Ito, K. (2016). Age-Related Changes in Attentional Control Using an N-Back Working Memory Paradigm. *Experimental Aging Research*, *42*(4), 390–402. https://doi.org/10.1080/0361073X.2016.1191867

Kaufmann, L., Ischebeck, A., Weiss, E., Koppelstaetter, F., Siedentopf, C., Vogel, S. E., Gotwald, T., Marksteiner, J., & Wood, G. (2008). An fMRI study of the numerical Stroop task in individuals with and without minimal cognitive impairment. *Cortex*, *44*(9), 1248–1255. https://doi.org/10.1016/j.cortex.2007.11.009

Kawai, N., Kubo-Kawai, N., Kubo, K., Terazawa, T., & Masataka, N. (2012). Distinct aging effects for two types of inhibition in older adults: A near-infrared spectroscopy study on the Simon task and the flanker task. *NeuroReport*, *23*(14), 819–824. https://doi.org/10.1097/WNR.0b013e3283578032

Keightley, M. L., Winocur, G., Burianova, H., Hongwanishkul, D., & Grady, C. L. (2006). Age effects on social cognition: Faces tell a different story. *Psychology and Aging*, *21*(3), 558–572. https://doi.org/10.1037/0882-7974.21.3.558

Kessels, R. P. C., Molleman, P. W., & Oosterman, J. M. (2011). Assessment of working-memory deficits in patients with mild cognitive impairment and Alzheimer’s dementia using Wechsler’s Working Memory Index. *Aging Clinical and Experimental Research*, *23*(5–6), 487–490. https://www.scopus.com/inward/record.uri?eid=2-s2.0-84865277704&partnerID=40&md5=028737952bd214a233df1062a9c3669f

Kessels, R. P. C., Overbeek, A., & Bouman, Z. (2015). Assessment of verbal and visuospatial working memory in mild cognitive impairment and Alzheimer’s dementia. *Dementia & Neuropsychologia*, *9*(3), 301–305. https://doi.org/10.1590/1980-57642015dn93000014

Kirchner, W. K. (1958). Age differences in short-term retention of rapidly changing information. *Journal of Experimental Psychology*, *55*(4), 352.

Kramer, J. H., Jurik, J., Sha, S. J., Rankin, K. P., Rosen, H. J., Johnson, J. K., & Miller, B. L. (2003). Distinctive Neuropsychological Patterns in Frontotemporal Dementia, Semantic Dementia, and Alzheimer Disease. *Cognitive and Behavioral Neurology*, *16*(4), 211–218. https://doi.org/10.1097/00146965-200312000-00002

Kramer, J. H., Nelson, A., Johnson, J. K., Yaffe, K., Glenn, S., Rosen, H. J., & Miller, B. L. (2006). Multiple cognitive deficits in amnestic mild cognitive impairment. *Dementia and Geriatric Cognitive Disorders*, *22*(4), 306–311. https://doi.org/10.1159/000095303

Kramer, J. H., Quitania, L., Dean, D., Neuhaus, J., Rosen, H. J., Halabi, C., Weiner, M. W., Magnotta, V. A., Delis, D. C., & Miller, B. L. (2007). Magnetic resonance imaging correlates of set shifting. *Journal of the International Neuropsychological Society*, *13*(3), 386–392. https://doi.org/10.1017/S1355617707070567

Kubo-Kawai, N., & Kawai, N. (2010). Elimination of the enhanced Simon effect for older adults in a three-choice situation: Ageing and the Simon effect in a go/no-go Simon task. *Quarterly Journal of Experimental Psychology*, *63*(3), 452–464. https://doi.org/10.1080/17470210902990829

Laguë-Beauvais, M., Brunet, J., Gagnon, L., Lesage, F., & Bherer, L. (2013). A fNIRS investigation of switching and inhibition during the modified Stroop task in younger and older adults. *NeuroImage*, *64*(1), 485–495. https://doi.org/10.1016/j.neuroimage.2012.09.042

Laguë-Beauvais, M., Fraser, S. A., Desjardins-Crépeau, L., Castonguay, N., Desjardins, M., Lesage, F., & Bherer, L. (2015). Shedding light on the effect of priority instructions during dual-task performance in younger and older adults: A fNIRS study. *Brain and Cognition*, *98*, 1–14. https://doi.org/10.1016/j.bandc.2015.05.001

Lambon Ralph, M. A., Patterson, K., Graham, N., Dawson, K., & Hodges, J. R. (2003). Homogeneity and heterogeneity in mild cognitive impairment and Alzheimer’s disease: A cross-sectional and longitudinal study of 55 cases. *Brain*, *126*(11), 2350–2362. https://doi.org/10.1093/brain/awg236

Langenecker, S. A., Briceno, E. M., Hamid, N. M., & Nielson, K. A. (2007). An evaluation of distinct volumetric and functional MRI contributions toward understanding age and task performance: A study in the basal ganglia. *Brain Research*, *1135*(1), 58–68. https://doi.org/10.1016/j.brainres.2006.11.068

Langenecker, S. A., & Nielson, K. A. (2003). Frontal recruitment during response inhibition in older adults replicated with fMRI. *NeuroImage*, *20*(2), 1384–1392. https://doi.org/10.1016/S1053-8119(03)00372-0

Langenecker, S. A., Nielson, K. A., & Rao, S. M. (2004). fMRI of healthy older adults during Stroop interference. In *NeuroImage* (Vol. 21, Issue 1). https://doi.org/10.1016/j.neuroimage.2003.08.027

Larson, G. E., Merritt, C. R., & Williams, S. E. (1988). Information processing and intelligence: Some implications of task complexity. *Intelligence*, *12*(2), 131–147. https://doi.org/10.1016/0160-2896(88)90012-8

Lee, M. S., Lee, S. H., Moon, E. O., Moon, Y. J., Kim, S., Kim, S. H., & Jung, I. K. (2013). Neuropsychological correlates of the P300 in patients with Alzheimer’s disease. *Progress in Neuro-Psychopharmacology and Biological Psychiatry*, *40*(1), 62–69. https://doi.org/10.1016/j.pnpbp.2012.08.009

Levinoff, E. J., Li, K. Z. H., Murtha, S., & Chertkow, H. (2004). Selective attention impairments in Alzheimer’s disease: Evidence for dissociable components. *Neuropsychology*, *18*(3), 580–588. https://doi.org/10.1037/0894-4105.18.3.580

Levinoff, E. J., Verret, L., Akerib, V., Phillips, N. A., Babins, L., Kelner, N., & Chertkow, H. (2006). Cognitive estimation impairment in Alzheimer disease and mild cognitive impairment. *Neuropsychology*, *20*(1), 123–132. https://doi.org/10.1037/0894-4105.20.1.123

Li, B. Y., Tang, H. D., & Chen, S. Di. (2016). Retrieval deficiency in brain activity of working memory in amnesic mild cognitive impairment patients: A Brain Event-Related Potentials Study. *Frontiers in Aging Neuroscience*, *8*(MAR), 1–10. https://doi.org/10.3389/fnagi.2016.00054

Li, C., Zheng, J., Wang, J., & Gui, L. (2011). Comparison between Alzheimer’s disease and subcortical vascular dementia: Attentional cortex study in functional magnetic resonance imaging. *Journal of International Medical Research*, *39*(4), 1413–1419. https://doi.org/10.1177/147323001103900428

Li, C., Zheng, J., Wang, J., Gui, L., & Li, C. (2009). An fMRI Stroop Task Study of Prefrontal Cortical Function in Normal Aging, Mild Cognitive Impairment, and Alzheimers Disease. *Current Alzheimer Research*, *6*(6), 525–530. https://doi.org/10.2174/156720509790147142

Liao, W., Zhang, X., Shu, H., Wang, Z., Liu, D., & Zhang, Z. J. (2017). The characteristic of cognitive dysfunction in remitted late life depression and amnestic mild cognitive impairment. *Psychiatry Research*, *251*, 168–175. https://doi.org/10.1016/j.psychres.2017.01.024

Loewenstein, D. A., Acevedo, A., Agron, J., Issacson, R., Strauman, S., Crocco, E., Barker, W. W., & Duara, R. (2006). Cognitive profiles in Alzheimer’s disease and in mild cognitive impairment of different etiologies. *Dementia and Geriatric Cognitive Disorders*, *21*(5–6), 309–315. https://doi.org/10.1159/000091522

Logan, G. D. (1988). Toward an instance theory of automatization. *Psychological Review*, *95*(4), 492–527. https://doi.org/10.1037//0033-295x.95.4.492

Logan, G. D., Cowan, W. B., & Davis, K. A. (1984). On the ability to inhibit simple and choice reaction time responses: A model and a method. *Journal of Experimental Psychology: Human Perception and Performance*, *10*(2), 276–291. https://doi.org/10.1037/0096-1523.10.2.276

Logie, R. H., Cocchini, G., Della Sala, S., & Baddeley, A. D. (2004). Is there a specific executive capacity for dual task coordination? Evidence from Alzheimer’s disease. *Neuropsychology*, *18*(3), 504–513. https://doi.org/10.1037/0894-4105.18.3.504

Lonie, J. A., Tierney, K. M., Herrmann, L. L., Donaghey, C., O’Carroll, R. E., Lee, A., & Ebmeier, K. P. (2009). Dual task performance in early Alzheimer’s disease, amnestic mild cognitive impairment and depression. In *Psychological Medicine* (Vol. 39, Issue 1). https://doi.org/10.1017/S0033291708003346

Lopez, O. L., Becker, J. T., Jagust, W. J., Fitzpatrick, A., Carlson, M. C., DeKosky, S. T., Breitner, J., Lyketsos, C. G., Jones, B., Kawas, C., & Kuller, L. H. (2006). Neuropsychological characteristics of mild cognitive impairment subgroups. *Journal of Neurology, Neurosurgery and Psychiatry*, *77*(2), 159–165. https://doi.org/10.1136/jnnp.2004.045567

Luks, T. L., Oliveira, M., Possin, K. L., Bird, A., Miller, B. L., Weiner, M. W., & Kramer, J. H. (2010). Atrophy in two attention networks is associated with performance on a Flanker task in neurodegenerative disease. *Neuropsychologia*, *48*(1), 165–170. https://doi.org/10.1016/j.neuropsychologia.2009.09.001

Lupker, S. J. (1979). The semantic nature of response competition in the picture-word interference task. *Memory & Cognition*, *7*(6), 485–495. https://doi.org/10.3758/BF03198265

MacPherson, S. E., Della Sala, S., & Logie, R. H. (2004). Dual-task interference of encoding and retrieval processes in healthy and impaired working memory. *Cortex; a Journal Devoted to the Study of the Nervous System and Behavior*, *40*(1), 183–184. https://doi.org/10.1016/S0010-9452(08)70943-2

MacPherson, S. E., Della Sala, S., Logie, R. H., & Wilcock, G. K. (2007). Specific AD impairments in concurrent performance of two memory tasks. *Cortex*, *43*(7), 858–865. https://doi.org/10.1016/S0010-9452(08)70685-3

Makizako, H., Doi, T., Shimada, H., Yoshida, D., Takayama, Y., & Suzuki, T. (2013). Relationship between dual-task performance and neurocognitive measures in older adults with mild cognitive impairment. *Geriatrics & Gerontology International*, *13*(2), 314–321. https://doi.org/10.1111/j.1447-0594.2012.00898.x

Mandzia, J. L., McAndrews, M. P., Grady, C., Graham, S. J., & Black, S. E. (2009). Neural correlates of incidental memory in mild cognitive impairment: An fMRI study. *Neurobiology of Aging*, *30*(5), 717–730. https://doi.org/10.1016/j.neurobiolaging.2007.08.024

Maquestiaux, F., Laguë-Beauvais, M., Ruthruff, E., Hartley, A., & Bherer, L. (2010). Learning to Bypass the Central Bottleneck: Declining Automaticity With Advancing Age. *Psychology and Aging*, *25*(1), 177–192. https://doi.org/10.1037/a0017122

Martyr, A., Boycheva, E., & Kudlicka, A. (2017). Assessing inhibitory control in early-stage Alzheimer’s and Parkinson’s disease using the Hayling Sentence Completion Test. *Journal of Neuropsychology*, 1–15. https://doi.org/10.1111/jnp.12129

Matías-Guiu, J. A., Cabrera-martín, M. N., Valles-salgado, M., Rognoni, T., & Galán, L. (2018). Inhibition impairment in frontotemporal dementia, amyotrophic lateral sclerosis, and Alzheimer’ s disease: clinical assessment and metabolic correlates. *Brain Imaging and Behavior*, *13*, 651–659. https://doi.org/10.1007/s11682-018-9891-3

Mayas, J., Fuentes, L. J., & Ballesteros, S. (2012). Stroop interference and negative priming (NP) suppression in normal aging. *Archives of Gerontology and Geriatrics*, *54*(2), 333–338. https://doi.org/10.1016/j.archger.2010.12.012

Mayr, U., & Keele, S. W. (2000). Changing internal constraints on action: The role of backward inhibition. *Journal of Experimental Psychology: General*, *129*(1), 4–26. https://doi.org/10.1037/0096-3445.129.1.4

McCabe, J., & Hartman, M. (2003). Examining the locus of age effects on complex span tasks. *Psychology and Aging*, *18*(3), 562–572. https://doi.org/10.1037/0882-7974.18.3.562

McCabe, J., & Hartman, M. (2008). Working memory for item and temporal information in younger and older adults. *Aging, Neuropsychology, and Cognition*, *15*(5), 574–600. https://doi.org/10.1080/13825580801956217

McGuinness, B., Barrett, S. L., Craig, D., & Lawson, J. (2010). Attention Deficits in Alzheimer’ s Disease and Vascular Dementia. *Journal of Neurology, Neurosurgery and Psychiatry*, *81*(2), 157–159. https://doi.org/10.1136/jnnp.2008.164483

Mehrotra, C. M., & Wagner, L. S. (2018). Psychology and Aging. *Aging and Diversity*, 102–160. https://doi.org/10.4324/9781315628097-3

Meléndez, J. C., Satorres, E., & Oliva, I. (2020). Comparing the Effect of Interference on an Emotional Stroop Task in Older Adults with and without Alzheimer’s Disease. *Journal of Alzheimer’s Disease*, *73*(4), 1445–1453. https://doi.org/10.3233/JAD-190989

Missonnier, P., Herrmann, F. R., Rodriguez, C., Deiber, M. P., Millet, P., Fazio-Costa, L., Gold, G., & Giannakopoulos, P. (2011). Age-related differences on event-related potentials and brain rhythm oscillations during working memory activation. *Journal of Neural Transmission*, *118*(6), 945–955. https://doi.org/10.1007/s00702-011-0600-2

Miyake, A., Friedman, N. P., Emerson, M. J., Witzki, A. H., Howerter, A., & Wager, T. D. (2000). The Unity and Diversity of Executive Functions and Their Contributions to Complex “Frontal Lobe” Tasks: A Latent Variable Analysis. *Cognitive Psychology*, *41*(1), 49–100. https://doi.org/10.1006/cogp.1999.0734

Monsell, S., & Mizon, G. A. (2006). Can the task-cuing paradigm measure an endogenous task-set reconfiguration process? *Journal of Experimental Psychology: Human Perception and Performance*, *32*(3), 493–516. https://doi.org/10.1037/0096-1523.32.3.493

Morris, N., & Jones, D. M. (1990). Memory updating in working memory: The role of the central executive. *British Journal of Psychology*, *81*, 111–121. https://doi.org/10.1421/34848

Morrone, I., Declercq, C., Novella, J. L., & Besche, C. (2010). Aging and inhibition processes: The case of metaphor treatment. *Psychology and Aging*, *25*(3), 697–701. https://doi.org/10.1037/a0019578

Muangpaisan, W., Intalapaporn, S., & Assantachai, P. (2010). Digit Span and Verbal Fluency tests in patients with Mild Cognitive Impairment and Normal Subjects in Thai-community. *Journal of the Medical Association of Thailand*, *93*(2), 224–230.

Mudar, R. A., Chiang, H. S., Eroh, J., Nguyen, L. T., Maguire, M. J., Spence, J. S., Kung, F., Kraut, M. A., & Hart, J. (2016). The effects of amnestic mild cognitive impairment on Go/NoGo semantic categorization task performance and event-related potentials. *Journal of Alzheimer’s Disease*, *50*(2), 577–590. https://doi.org/10.3233/JAD-150586

Müller, L. D., Guhn, A., Zeller, J. B. M., Biehl, S. C., Dresler, T., Hahn, T., Fallgatter, A. J., Polak, T., Deckert, J., & Herrmann, M. J. (2014). Neural correlates of a standardized version of the trail making test in young and elderly adults: A functional near-infrared spectroscopy study. *Neuropsychologia*, *56*(1), 271–279. https://doi.org/10.1016/j.neuropsychologia.2014.01.019

Nagahama, Y., Okina, T., Suzuki, N., Matsuzaki, S., Yamauchi, H., Nabatame, H., & Matsuda, M. (2003). Factor structure of a modified version of the Wisconsin Card Sorting Test: an analysis of executive deficit in Alzheimer’s disease and mild cognitive impairment. *Dementia and Geriatric Cognitive Disorders*, *16*, 103–112.

Nagel, I. E., Preuschhof, C., Li, S. C., Nyberg, L., Bäckman, L., Lindenberger, U., & Heekeren, H. R. (2011). Load modulation of BOLD response and connectivity predicts working memory performance in younger and older adults. *Journal of Cognitive Neuroscience*, *23*(8), 2030–2045. https://doi.org/10.1162/jocn.2010.21560

Nelson, H. E. (1976). A modified card sorting test sensitive to frontal lobe defects. *Cortex*, *12*(4), 313–324.

Newman, J. P., & Kosson, D. S. (1986). Passive Avoidance Learning in Psychopathic and Nonpsychopathic Offenders. *Journal of Abnormal Psychology*, *95*(3), 252–256. https://doi.org/10.1037/0021-843X.95.3.252

Nguyen, L. T., Mudar, R. A., Chiang, H. S., Schneider, J. M., Maguire, M. J., Kraut, M. A., & Hart, J. (2017). Theta and alpha alterations in amnestic mild cognitive impairment in semantic Go/NoGo tasks. *Frontiers in Aging Neuroscience*, *9*(160). https://doi.org/10.3389/fnagi.2017.00160

Nielson, K. A., Langenecker, S. A., & Garavan, H. (2002). Differences in the functional neuroanatomy of inhibitory control across the adult life span. *Psychology and Aging*, *17*(1), 56–71. https://doi.org/10.1037/0882-7974.17.1.56

Nielson, K. A., Langenecker, S. A., Ross, T. J., Garavan, H., Rao, S. M., & Stein, E. A. (2004). Comparability of functional MRI response in young and old during inhibition. *NeuroReport*, *15*(1), 129–133. https://doi.org/10.1097/00001756-200401190-00025

Noiret, N., Carvalho, N., Laurent, É., Chopard, G., Binetruy, M., Nicolier, M., Monnin, J., Magnin, E., & Vandel, P. (2018). Saccadic eye movements and attentional control in Alzheimer’s disease. *Archives of Clinical Neuropsychology*, *33*(1), 1–13. https://doi.org/10.1093/arclin/acx044

Nordlund, A., Rolstad, S., Hellström, P., Sjögren, M., Hansen, S., & Wallin, A. (2005). The Goteborg MCI study: Mild cognitive impairment is a heterogeneous condition. *Journal of Neurology, Neurosurgery and Psychiatry*, *76*(11), 1485–1490. https://doi.org/10.1136/jnnp.2004.050385

Oosterman, J. M., Boeschoten, M. S., Eling, P. A. T., Kessels, R. P. C., & Maes, J. H. R. (2014). Simple and complex rule induction performance in young and older adults: Contribution of episodic memory and working memory. *Journal of the International Neuropsychological Society*, *20*(3), 333–341. https://doi.org/10.1017/S1355617713001446

Opwonya, J., Wang, C., Jang, K. M., Lee, K., Kim, J. Il, & Kim, J. U. (2022). Inhibitory Control of Saccadic Eye Movements and Cognitive Impairment in Mild Cognitive Impairment. *Frontiers in Aging Neuroscience*, *14*(April), 1–12. https://doi.org/10.3389/fnagi.2022.871432

Pa, J., Possin, K. L., Wilson, S. M., Quitania, L. C., Kramer, J. H., Boxer, A. L., Weiner, M. W., & Johnson, J. K. (2010). Gray matter correlates of set-shifting among neurodegenerative disease, mild cognitive impairment, and healthy older adults. *Journal of the International Neuropsychological Society*, *16*(4), 640–650. https://doi.org/10.1017/S1355617710000408

Pashler, H. (1994). Dual-Task Interference in Simple Tasks: Data and Theory. *Psychological Bulletin*, *116*(2), 220–244. https://doi.org/10.1037/0033-2909.116.2.220

Peltsch, A., Hemraj, A., Garcia, A., & Munoz, D. P. (2014). Saccade deficits in amnestic mild cognitive impairment resemble mild Alzheimer’s disease. *European Journal of Neuroscience*, *39*(11), 2000–2013. https://doi.org/10.1111/ejn.12617

Peng, Y., Zhu, Q., Wang, B., & Ren, J. (2020). A cross-sectional study on interference control: Age affects reactive control but not proactive control. *PeerJ*, *2020*(1), 1–14. https://doi.org/10.7717/peerj.8365

Pereiro, A. X., Juncos-Rabadán, O., & Facal, D. (2014). Attentional control in amnestic MCI subtypes: Insights from a simon task. *Neuropsychology*, *28*(2), 261–272. https://doi.org/10.1037/neu0000047

Perry, R. J., Watson, P., & Hodges, J. R. (2000). The nature and staging of attention dysfunction in early (minimal and mild) Alzheimer’s disease: Relationship to episodic and semantic memory impairment. *Neuropsychologia*, *38*(3), 252–271. https://doi.org/10.1016/S0028-3932(99)00079-2

Peters, F., Villeneuve, S., & Belleville, S. (2014). Predicting progression to dementia in elderly subjects with mild cognitive impairment using both cognitive and neuroimaging predictors. *Journal of Alzheimer’s Disease*, *38*(2), 307–318. https://doi.org/10.3233/JAD-130842

Pettigrew, C., & Martin, R. C. (2014). Cognitive declines in healthy aging: Evidence from multiple aspects of interference resolution. *Psychology and Aging*, *29*(2), 187–204. https://doi.org/10.1037/a0036085

Pitarque, A., Meléndez, J. C., Sales, A., Mayordomo, T., Satorres, E., Escudero, J., & Algarabel, S. (2016). The effects of healthy aging, amnestic mild cognitive impairment, and Alzheimer’s disease on recollection, familiarity and false recognition, estimated by an associative process-dissociation recognition procedure. *Neuropsychologia*, *91*, 29–35. https://doi.org/10.1016/j.neuropsychologia.2016.07.010

Price, J. L., McKeel, D. W., Buckles, V. D., Roe, C. M., Xiong, C., Grundman, M., Hansen, L. A., Petersen, R. C., Parisi, J. E., Dickson, D. W., Smith, C. D., Davis, D. G., Schmitt, F. A., Markesbery, W. R., Kaye, J., Kurlan, R., Hulette, C., Kurland, B. F., Higdon, R., … Morris, J. C. (2009). Neuropathology of nondemented aging: Presumptive evidence for preclinical Alzheimer disease. *Neurobiology of Aging*, *30*(7), 1026–1036. https://doi.org/10.1016/j.neurobiolaging.2009.04.002

Price, S. E., Kinsella, G. J., Ong, B., Mullaly, E., Phillips, M., Pangnadasa-Fox, L., Perre, D., & Storey, E. (2010). Learning and memory in amnestic mild cognitive impairment: Contribution of working memory. *Journal of the International Neuropsychological Society*, *16*(2), 342–351. https://doi.org/10.1017/S1355617709991391

Puente, A. N., Faraco, C., Terry, D. P., Brown, C., & Miller, L. S. (2014). Minimal functional brain differences between older adults with and without mild cognitive impairment during the Stroop. *Aging, Neuropsychology, and Cognition*, *21*(3), 346–369. https://doi.org/10.1080/13825585.2013.824065

Ramsden, C. M., Kinsella, G. J., Ong, B., & Storey, E. (2008). Performance of Everyday Actions in Mild Alzheimer’s Disease. *Neuropsychology*, *22*(1), 17–26. https://doi.org/10.1037/0894-4105.22.1.17

Redondo, M. T., Beltrán-Brotóns, J. L., Reales, J. M., & Ballesteros, S. (2016). Executive functions in patients with Alzheimer’s disease, type 2 diabetes mellitus patients and cognitively healthy older adults. *Experimental Gerontology*, *83*, 47–55. https://doi.org/10.1016/j.exger.2016.07.013

Reitan, R. M. (1992). Trail Making Test: Manual for administration and scoring. In *Reitan Neuropsychology Laboratory*.

Reitan, R. M., & Wolfson, D. (1986). Review of The Halstead-Reitan Neuropsychological Test Battery: Theory and Clinical Interpretation. In *PsycCRITIQUES* (Vol. 31, Issue 4, pp. 309–309). https://doi.org/10.1037/024717

Ren, Y., Ren, Y., Yang, W., Tang, X., Wu, F., Wu, Q., Takahashi, S., Ejima, Y., & Wu, J. (2018). Comparison for younger and older adults: Stimulus temporal asynchrony modulates audiovisual integration. *International Journal of Psychophysiology*, *124*(March 2017), 1–11. https://doi.org/10.1016/j.ijpsycho.2017.12.004

Ren, Y., Yang, W., Nakahashi, K., Takahashi, S., & Wu, J. (2017). Audiovisual Integration Delayed by Stimulus Onset Asynchrony Between Auditory and Visual Stimuli in Older Adults. *Perception*, *46*(2), 205–218. https://doi.org/10.1177/0301006616673850

Rey-Mermet, A., Gade, M., & Oberauer, K. (2018). Should we stop thinking about inhibition? Searching for individual and age differences in inhibition ability. *Journal of Experimental Psychology: Learning Memory and Cognition*, *44*(4), 501–526. https://doi.org/10.1037/xlm0000450

Rhodes, M. G., & Kelley, C. M. (2005). Executive processes, memory accuracy, and memory monitoring: An aging and individual difference analysis. *Journal of Memory and Language*, *52*(4), 578–594. https://doi.org/10.1016/j.jml.2005.01.014

Roberts, R. J., Hager, L. D., & Heron, C. (1994). Prefrontal Cognitive Processes: Working Memory and Inhibition in the Antisaccade Task. *Journal of Experimental Psychology: General*, *123*(4), 374–393. https://doi.org/10.1037/0096-3445.123.4.374

Robertson, I. H., Ward, T., Ridgeway, V., & Nimmo-Smith, I. (1994). The Test of Everyday Attention: TEA. *October*, *4*(1), 51–55. https://www.researchgate.net/publication/267552527

Robertson, I. H., Ward, T., Ridgeway, V., & Nimmo-Smith, I. (2001). The Test of Everyday Attention. Manual Atlantic Fellows for Equity in Brain Health View project Mathematical Statistics View project. *Test Reviews*, *4*(1), 51–55. https://www.researchgate.net/publication/267552527

Rogers, R. D., & Monsell, S. (1995). Costs of a Predictable Switch Between Simple Cognitive Tasks. *Journal of Experimental Psychology: General*, *124*(2), 207–231. https://doi.org/10.1037/0096-3445.124.2.207

Rose, N. S., Myerson, J., Sommers, M. S., & Hale, S. (2009). Are there age differences in the Executive Component of Working Memory? Evidence from Domain-General Interference Effects. *Aging, Neuropsychology, and Cognition*, *16*(6), 633–653. https://doi.org/10.1080/13825580902825238

Salat, D. H., Kaye, J. A., & Janowsky, J. S. (2002). Greater orbital prefrontal volume selectively predicts worse working memory performance in older adults. *Cerebral Cortex*, *12*(5), 494–505. https://doi.org/10.1093/cercor/12.5.494

Salthouse, T. A. (2010). Is flanker-based inhibition related to age? Identifying specific influences of individual differences on neurocognitive variables. *Brain and Cognition*, *73*(1), 51–61. https://doi.org/10.1016/j.bandc.2010.02.003

Salthouse, T. A., Fristoe, N., McGuthry, K. E., & Hambrick, D. Z. (1998). Relation of task switching to speed, age, and fluid intelligence. *Psychology and Aging*, *13*(3), 445–461. https://doi.org/10.1037/0882-7974.13.3.445

Salthouse, T. A., & Meinz, E. J. (1995). Aging, inhibition, working memory, and speed. *Journals of Gerontology - Series B Psychological Sciences and Social Sciences*, *50 B*(6), P297–P306. https://doi.org/10.1093/geronb/50B.6.P297

Satorres, E., Oliva, I., Escudero, J., & Meléndez, J. C. (2020). Conflict monitoring on an emotional Stroop task. Comparison of healthy older adults and patients with major neurocognitive disorders due to probable AD. *Journal of Clinical and Experimental Neuropsychology*, *42*(5), 485–494. https://doi.org/10.1080/13803395.2020.1761946

Schlaghecken, F., Birak, K. S., & Maylor, E. A. (2012). Age-related deficits in efficiency of low-level lateral inhibition. *Frontiers in Human Neuroscience*, *6*(APRIL 2012), 1–9. https://doi.org/10.3389/fnhum.2012.00102

Schmitter-Edgecombe, M., & Sanders, C. (2009). Task switching in mild cognitive impairment: Switch and nonswitch costs. *Journal of the International Neuropsychological Society*, *15*(1), 103–111. https://doi.org/10.1017/S1355617708090140

Schriefers, H., Meyer, A. S., & Levelt, W. J. M. (1990). Exploring the time course of lexical access in language production: Picture-word interference studies. *Journal of Memory and Language*, *29*(1), 86–102. https://doi.org/10.1016/0749-596X(90)90011-N

Schroeder, P. J. (2014). The effects of age on processing and storage in working memory span tasks and reading comprehension. *Experimental Aging Research*, *40*(3), 308–331. https://doi.org/10.1080/0361073X.2014.896666

Sebastian, M. V., Menor, J., & Elosua, M. R. (2006). Attentional dysfunction of the central executive in AD: Evidence from dual task and perseveration errors. *Cortex*, *42*(7), 1015–1020. https://doi.org/10.1016/S0010-9452(08)70207-7

Servant, M., & Evans, N. J. (2020). A diffusion model analysis of the effects of aging in the flanker task. *Psychology and Aging*, *35*(6), 831–849. https://doi.org/10.1037/pag0000546

Shafiq-Antonacci, R., Maruff, P., Masters, C., & Currie, J. (2003). Spectrum of saccade system function in Alzheimer disease. *Archives of Neurology*, *60*(9), 1272–1278. https://doi.org/10.1001/archneur.60.9.1272

Silveri, M. C., Reali, G., Jenner, C., & Puopolo, M. (2007). Attention and memory in the preclinical stage of dementia. *Journal of Geriatric Psychiatry and Neurology*, *20*(2), 67–75. https://doi.org/10.1177/0891988706297469

Simon, J. R. (1969). Reactions toward the source of stimulation. *Journal of Experimental Psychology*, *81*(1), 174–176. https://doi.org/10.1037/h0027448

Sinai, M., Phillips, N. A., Chertkow, H., & Kabani, N. J. (2010). Task Switching Performance Reveals Heterogeneity Amongst Patients With Mild Cognitive Impairment. *Neuropsychology*, *24*(6), 757–774. https://doi.org/10.1037/a0020314

Skinner, E. I., & Fernandes, M. A. (2008). Interfering with remembering and knowing: Effects of divided attention at retrieval. *Acta Psychologica*, *127*(2), 211–221. https://doi.org/10.1016/j.actpsy.2007.05.001

Smits, L. L., Van Harten, A. C., Pijnenburg, Y. A. L., Koedam, E. L. G. E., Bouwman, F. H., Sistermans, N., Reuling, I. E. W., Prins, N. D., Lemstra, A. W., Scheltens, P., & Van Der Flier, W. M. (2015). Trajectories of cognitive decline in different types of dementia. *Psychological Medicine*, *45*(5), 1051–1059. https://doi.org/10.1017/S0033291714002153

Souchay, C., & Isingrini, M. (2004). Age related differences in metacognitive control: Role of executive functioning. *Brain and Cognition*, *56*(1), 89–99. https://doi.org/10.1016/j.bandc.2004.06.002

Spector, A., & Biederman, I. (1976). Mental set and mental shift revisited. *The American Journal of Psychology*, *89*(4), 669–679.

Spreen, O., & Strauss, E. (1998). *A compendium of neuropsychological tests: Administration, norms, and commentary*.

Stokholm, J., Vogel, A., Gade, A., & Waldemar, G. (2006). Heterogeneity in executive impairment in patients with very mild Alzheimer’s disease. *Dementia and Geriatric Cognitive Disorders*, *22*(1), 54–59. https://doi.org/10.1159/000093262

Strobach, T., Frensch, P., Müller, H. J., & Schubert, T. (2012a). Testing the Limits of Optimizing Dual-Task Performance in Younger and Older Adults. *Frontiers in Human Neuroscience*, *6*. https://doi.org/10.3389/fnhum.2012.00039

Strobach, T., Frensch, P., Müller, H., & Schubert, T. (2012b). Age- and practice-related influences on dual-task costs and compensation mechanisms under optimal conditions of dual-task performance. *Aging, Neuropsychology, and Cognition*, *19*(1–2), 222–247. https://doi.org/10.1080/13825585.2011.630973

Stroop, J. R. (1935). Studies of interference in serial verbal reactions. *Journal of Experimental Psychology*, *18*(6), 643–662. https://doi.org/10.1037/h0054651

Sung, J. E., Kim, J. H., Jeong, J. H., & Kang, H. (2012). Working memory capacity and its relation to Stroop interference and facilitation effects in individuals with mild cognitive impairment. *American Journal of Speech-Language Pathology*, *21*(2). https://doi.org/10.1044/1058-0360(2012/11-0101)

Sylvain-Roy, S., Lungu, O., & Belleville, S. (2015). Normal Aging of the Attentional Control Functions That Underlie Working Memory. *The Journals of Gerontology Series B: Psychological Sciences and Social Sciences*, *70*(5), 698–708. https://doi.org/10.1093/geronb/gbt166

Taconnat, L., Raz, N., Toczé, C., Bouazzaoui, B., Sauzéon, H., Fay, S., & Isingrini, M. (2009). Ageing and organisation strategies in free recall: The role of cognitive flexibility. *European Journal of Cognitive Psychology*, *21*(2–3), 347–365. https://doi.org/10.1080/09541440802296413

Tipper, S. P. (1985). The Negative Priming Effect. Inhibitory Priming By Ignored Objects. *The Quarterly Journal of Experimental Psychology*, *37A*, 571–590.

Tournier, I., Postal, V., & Mathey, S. (2014). Investigation of age-related differences in an adapted Hayling task. *Archives of Gerontology and Geriatrics*, *59*, 599–606. https://doi.org/10.1016/j.archger.2014.07.016

Traykov, L., Baudic, S., Thibaudet, M. C., Rigaud, A. S., Smagghe, A., & Boller, F. (2002). Neuropsychological deficit in early subcortical vascular dementia: Comparison to Alzheimer’s disease. *Dementia and Geriatric Cognitive Disorders*, *14*(1), 26–32. https://doi.org/10.1159/000058330

Traykov, L., Raoux, N., Latour, F., Gallo, L., Baudic, S., Bayle, C., Wenisch, E., Remy, P., & Hanon, O. (2007). Executive functions deficit in mild cognitive impairment. *Cognitive and Behavioral Neurology*, *20*(4), 219–224. https://doi.org/10.1097/WNN.0b013e31815e6254

Tsai, C. L., Pai, M. C., Ukropec, J., & Ukropcová, B. (2016). The Role of Physical Fitness in the Neurocognitive Performance of Task Switching in Older Persons with Mild Cognitive Impairment. *Journal of Alzheimer’s Disease*, *53*(1), 143–159. https://doi.org/10.3233/JAD-151093

Tse, C. S., Balota, D. A., Yap, M. J., Duchek, J. M., & McCabe, D. P. (2010). Effects of healthy aging and early stage dementia of the alzheimer’s type on components of response time distributions in three attention tasks. *Neuropsychology*, *24*(3), 300–315. https://doi.org/10.1037/a0018274

Turner, M. L., & Engle, R. W. (1989). Is working memory capacity task dependent? *Journal of Memory and Language*, *28*(2), 127–154. https://doi.org/10.1016/0749-596X(89)90040-5

Vallesi, A., Hasher, L., & Stuss, D. T. (2010). Age-Related Differences in Transfer Costs: Evidence From Go/Nogo Tasks. *Psychology and Aging*, *25*(4), 963–967. https://doi.org/10.1037/a0020300

Van’t Ent, D. (2002). Perceptual and motor contributions to performance and {ERP} components after incorrect motor activation in a flanker reaction task. *Clinical Neurophysiology*, *113*, 270–283.

Van Dam, N. T., Sano, M., Mitsis, E. M., Grossman, H. T., Gu, X., Park, Y., Hof, P. R., & Fan, J. (2013). Functional Neural Correlates of Attentional Deficits in Amnestic Mild Cognitive Impairment. *PLoS ONE*, *8*(1), 1–12. https://doi.org/10.1371/journal.pone.0054035

Vaughan, L., Basak, C., Hartman, M., & Verhaeghen, P. (2008). Aging and working memory inside and outside the focus of attention: Dissociations of availability and accessibility. *Aging, Neuropsychology, and Cognition*, *15*(6), 703–724. https://doi.org/10.1080/13825580802061645

Velichkovsky, B. B., Tatarinov, D. V., Roshchina, I. F., & Selezneva, N. D. (2020). Increased local switch costs in mild cognitive impairment. *Experimental Gerontology*, *135*(January), 110934. https://doi.org/10.1016/j.exger.2020.110934

Wang, B., Li, P., Li, D., Niu, Y., Yan, T., Li, T., Cao, R., Yan, P., Guo, Y., Yang, W., Ren, Y., Li, X., Wang, F., Yan, T., Wu, J., Zhang, H., & Xiang, J. (2018). Increased functional brain network efficiency during audiovisual temporal asynchrony integration task in aging. *Frontiers in Aging Neuroscience*, *10*(OCT), 1–15. https://doi.org/10.3389/fnagi.2018.00316

Wang, P., Zhang, X., Liu, Y., Liu, S., Zhou, B., Zhang, Z., Yao, H., Zhang, X., & Jiang, T. (2013). Perceptual and response interference in Alzheimer’s disease and mild cognitive impairment. *Clinical Neurophysiology*, *124*(12), 2389–2396. https://doi.org/10.1016/j.clinph.2013.05.014

Wang, Z., & Su, Y. (2013). Age-related differences in the performance of theory of mind in older adults: A dissociation of cognitive and affective components. *Psychology and Aging*, *28*(1), 284–291. https://doi.org/10.1037/a0030876

Ward, N., Hussey, E., Alzahabi, R., Gaspar, J. G., & Kramer, A. F. (2021). Age-related effects on a novel dual-task Stroop paradigm. *PLoS ONE*, *16*(3 March), 1–15. https://doi.org/10.1371/journal.pone.0247923

Waring, J. D., Greif, T. R., & Lenze, E. J. (2019). Emotional response inhibition is greater in older than younger adults. *Frontiers in Psychology*, *10*(APR), 1–13. https://doi.org/10.3389/fpsyg.2019.00961

Wechsler, D. (1987). *Manual for the Wechsler Memory Scale-Revised*. The Psychological Corporation.

Wechsler, D. (2012). *Manual for the Wechsler Adult Intelligence Scale – Fourth Edition*. Psychological Corp.

Welford, A. T. (1952). The psychological refractory period and the timing of high- speed performance - A review and a theory. *British Journal of Social Psycholology*, *43*, 2–19. https://doi.org/10.1111/j.2044-8295.1952.tb00322.x

Williams, B. R., Ponesse, J. S., Schachar, R. J., Logan, G. D., & Tannock, R. (1999). Development of inhibitory control across the life span. *Developmental Psychology*, *35*(1), 205–213. https://doi.org/10.1037/0012-1649.35.1.205

Williams, S. E., Lenze, E. J., & Waring, J. D. (2020). Positive information facilitates response inhibition in older adults only when emotion is task-relevant. *Cognition and Emotion*, 1632–1645. https://doi.org/10.1080/02699931.2020.1793303

Wilson, B. A., Alderman, N., Burgess, P. W., Emslie, H., & Evans, J. J. (1996). *BADS: Behavioural assessment of the dysexecutive syndrome*. Thames Valley Test Company.

Wylie, S. A., Ridderinkhof, K. R., Eckerle, M. K., & Manning, C. A. (2007). Inefficient response inhibition in individuals with mild cognitive impairment. *Neuropsychologia*, *45*(7), 1408–1419. https://doi.org/10.1016/j.neuropsychologia.2006.11.003

Yeom, T. H., Park, Y. S., Oh, K. J., Kim, J. K., & Lee, Y. H. (1992). *A manual for K-WAIS*. Korean Guidance.

Yntema, D. B. (1963). Keeping track of several things at once. *Human Factors*, *5*, 7–17.

Yordanova, J., Gajewski, P. D., Getzmann, S., Kirov, R., Falkenstein, M., & Kolev, V. (2021). Neural Correlates of Aging-Related Differences in Pro-active Control in a Dual Task. *Frontiers in Aging Neuroscience*, *13*(September), 1–12. https://doi.org/10.3389/fnagi.2021.682499

Yuan, B., Xie, C., & Zhang, Z. (2016). Mediation of Episodic Memory Performance by The Executive Function Network in Patients with Amnestic Mild Cognitive Impairment: A Resting-State Functional MRI Study. *Alzheimer’s & Dementia*, *12*(40), P32–P32. https://doi.org/10.1016/j.jalz.2016.06.044

Yun, J.-Y., Lee, D. Y., Seo, E. H., Choo, I. H., Park, S. Y., Kim, S. G., & Woo, J. I. (2011). Neural Correlates of Stroop Performance in Alzheimer’s Disease: A FDG-PET Study. *Dementia and Geriatric Cognitive Disorders Extra*, *1*(1), 190–201. https://doi.org/10.1159/000329517

Zamarian, L., Semenza, C., Domahs, F., Benke, T., & Delazer, M. (2007). Alzheimer’s disease and mild cognitive impairment: Effects of shifting and interference in simple arithmetic. *Journal of the Neurological Sciences*, *263*(1–2), 79–88. https://doi.org/10.1016/j.jns.2007.06.005

Zhang, Y., Han, B., Verhaeghen, P., & Nilsson, L. G. (2007). Executive functioning in older adults with mild cognitive impairment: MCI has effects on planning, but not on inhibition. *Aging, Neuropsychology, and Cognition*, *14*(6), 557–570. https://doi.org/10.1080/13825580600788118

Zheng, D., Dong, X., Sun, H., Xu, Y., Ma, Y., & Wang, X. (2012). The overall impairment of core executive function components in patients with amnestic mild cognitive impairment: A cross-sectional study. *BMC Neurology*, *12*(138). https://doi.org/10.1186/1471-2377-12-138

Zheng, D., Sun, H., Dong, X., Liu, B., Xu, Y., Chen, S., Song, L., Zhang, H., & Wang, X. (2014). Executive dysfunction and gray matter atrophy in amnestic mild cognitive impairment. *Neurobiology of Aging*, *35*(3), 548–555. https://doi.org/10.1016/j.neurobiolaging.2013.09.007

Zhou, A., & Jia, J. (2009). Different cognitive profiles between mild cognitive impairment due to cerebral small vessel disease and mild cognitive impairment of Alzheimer’s disease origin. *Journal of the International Neuropsychological Society*, *15*(6), 898–905. https://doi.org/10.1017/S1355617709990816
